# Toughening elastomer via sequentially activated multi-pathway energy dissipation

**DOI:** 10.1038/s41467-026-74148-z

**Published:** 2026-07-01

**Authors:** Xue Li, Chunlin Xiao, Haruki Izutsu, Osamu Urakawa, Tadashi Inoue, Yuichiro Kobayashi, Hiroyasu Yamaguchi

**Affiliations:** 1 Department of Macromolecular Science, Graduate School of Science, The University of Osaka, Toyonaka, Osaka Japan; 2 Forefront Research Center, Graduate School of Science, The University of Osaka, Toyonaka, Osaka Japan; 3 Innovative Catalysis Science Division, Institute for Open and Transdisciplinary Research Initiatives (ICS-OTRI); The University of Osaka, Suita, Osaka Japan

**Keywords:** Polymers, Interlocked molecules, Polymers

## Abstract

Auxiliary energy dissipation pathways have been incorporated into elastomer design to enhance the toughness. However, most strategies involving multiple pathways rely on non-synergistic dissipation that ceases to function once structural evolution occurs. Herein, we report a strategy to toughen elastomer via sequentially activated multi-pathway energy dissipation, simultaneously integrating mechanically interlocked networks (MINs), scissile mechanophore, and woven networks within a single polymer. A rotaxane crosslinker is applied to construct MINs in polyurethane as the primary energy dissipation pathway. After the sliding motion of the rotaxane crosslinkers reaches its maximal extent, the truxinate mechanophore embedded in rotaxane’s macrocycle undergoes sacrificial scission as the secondary pathway. Subsequently, the linear chain produced by cleavage of the rotaxane’s macrocycle inherently entangles with the axle chain, resulting in woven networks as the tertiary energy dissipation pathway. This strategy substantially enhances the toughness and clarifies how mechanophore activation interacts with supramolecular architecture in polymeric materials.

## Abstract (web summary)

Auxiliary energy dissipation pathways can enhance toughness of elastomers but most strategies rely on non-synergistic dissipation that ceases to function once structural evolution occurs. Here, the authors report a toughening strategy via sequentially activated multipathway energy dissipation, simultaneously integrating mechanically interlocked networks, mechanophore, and woven networks within a single polymer.

## Introduction

Polymeric materials have long been employed in diverse fields, attracting sustained interest from researchers. Elastomers, as one of the polymer classes with the longest history of utilization and study, have consistently attracted attention for their outstanding toughness, promoting wide applications across diverse fields^1–3^. Over the past decades, many molecular-level strategies have been developed to enhance the toughness of polymeric materials^4–7^. In recent years, mechanically interlocked networks (MINs) have been incorporated into polymeric materials owing to the high degrees of conformational freedom and mobility derived from the mechanical bonds^8–11^. For example, rotaxane- and catenane-crosslinked elastomers exhibit remarkable energy dissipation capacity^12–17^. The incorporation of scissile mechanophore into polymer backbones has also been developed, where sacrificial scission under external stress enables energy dissipation, resulting in the increase of both toughness and durability^18–25^. In addition, woven networks have been shown to effectively improve the toughness and puncture resistance of elastomers^26–32^.

However, some limitations remain in both the structural design and materials characterization. For rotaxane-crosslinked polymers, once macrocycle sliding along the axle is arrested by the stoppers, energy dissipation approaches its limit, leaving the network vulnerable unless additional pathways are available^33^. Moreover, local stress concentration in supramolecular motifs may even activate otherwise unreactive chemical bonds, inducing undesirable covalent scission and leading to rapid network collapse^34,35^. In addition, although mechanophores have been widely incorporated in polymer backbone to enhance the mechanical performance, studies on their force-induced response in supramolecular polymer systems have so far been mostly confined to the molecular level^34–37^. How the structural evolution of mechanophores interacts with supramolecular architecture and thereby affects mechanical performance remains largely unexplored. On the other hand, applying the mechanophore as a crosslinker is also in the face of losing topology consistency of the polymer network during the mechanical response^38^. Furthermore, woven polymer networks (WPNs), in which structurally well-defined chain entanglements are introduced through molecular design, dissipating energy by chain slippage and rearrangement of tension, are generally limited in both their stress resistance and the programmability of dissipation pathways^26,39,40^. Moreover, efforts to integrate multiple pathways, including MINs, mechanophores, and woven networks, remain scarce, and the toughening mechanisms when multiple (three) distinct types of energy dissipation pathways coexist remain inadequately elucidated. Although MINs and scissile mechanophores have been successfully combined within a single crosslinker for elastomers, the resulting toughening effect is inferior to that achieved by either component alone, indicating the absence of a strategy to enable their synergistic toughening^33^.

**Fig. 1:**
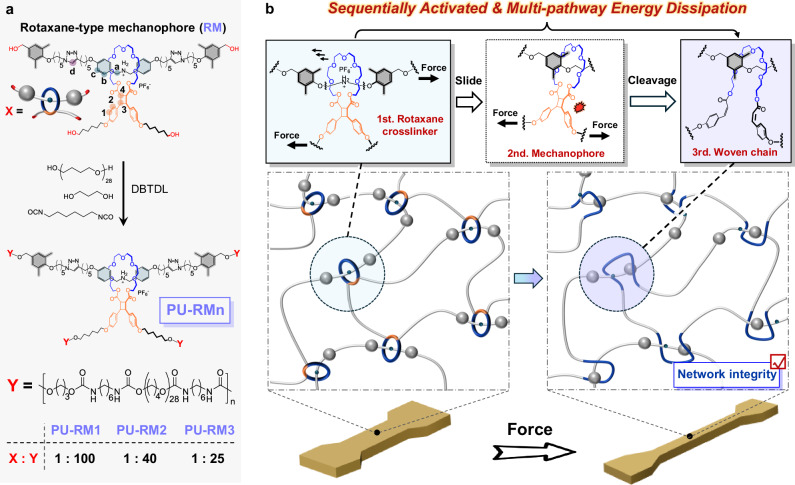
Design and preparation of PU-RMn with sequentially activated multi-pathway energy dissipation. **a** Preparation of PU-RMn. **b** Evolution of the network structure under stress: the slide of polymer-chain-tethered macrocycle along the axle chain serves as the primary energy dissipation pathway; the sacrificial scission of mechanophore serves as the secondary energy dissipation pathway; the inherently converted woven networks serves as the tertiary energy dissipation pathway.

In this work, we report a strategy that integrates these three energy dissipation pathways into elastomers, achieving synergistic toughening of MINs and mechanophores by enabling their sequential activation under external stress (MINs, mechanophores, and WPNs in sequence), thereby enhancing toughness (Fig. 1). Specifically, inspired by the introduction of cyclobutane cores into crown ether backbones, we designed and synthesized a rotaxane crosslinker, of which macrocycle is cyclized by truxinate as the scissile mechanophore^22,33,41,42^. MINs were constructed by incorporating the rotaxane crosslinker into the polycondensation for polyurethane preparation. Under external stress, the system first dissipates energy through the sliding of the polymer-chain-tethered macrocycle along the axle of the rotaxane crosslinker. Once the slide reaches its limit, where the macrocycle is blocked by the axle stopper, the truxinate moiety on the macrocycle undergoes selective scission with increased stress, acting as a secondary energy dissipation pathway. Subsequently, the linear chain produced by cleavage of the rotaxane’s macrocycle inherently entangle with the axle chain (Supplementary Fig. 7)^29,30,43^. Therefore, MINs gradually convert into woven networks as rotaxane crosslinkers rupture, preserving network integrity and serving as the tertiary energy dissipation pathway. To the best of our knowledge, this work represents the first example of a single polymeric material simultaneously integrating mechanically interlocked networks, a scissile mechanophore, and woven networks through rational molecular design. We further elucidate the correlation between rotaxane sliding, mechanochemical scission, and the resulting macroscopic mechanical behavior by synergistic analysis based on mechanical, rheological, and spectroscopic characterizations.

## Results

### Design, synthesis, and structural characterization

First, the macrocycle-type mechanophore (MCM) was synthesized by ring closure through an intramolecular [2 + 2] photocycloaddition of cinnamate moieties. In the resulting macrocycle, the glycol fragment serves to recognize the axle (compound G) containing a dibenzylammonium cation (DBA^+^), and the truxinate moiety arising from the photoreaction acts as the mechanochemically cleavable site. Next, the rotaxane-type mechanophore (RM) was obtained in one pot via a copper-catalyzed azide–alkyne cycloaddition (CuAAC), where axle and MCM first form a *pseudo*-rotaxane complex that subsequently clicks with stoppers to afford the rotaxane. RM was then used as a crosslinker and introduced into polyurethane through polycondensation, producing crosslinked polymers PU-RMn (*n* = 1-3), where the molar percentage of RM unit in PU-RM1, PU-RM2, and PU-RM3 is 1.0%, 2.5%, and 4.0%, respectively.

**Fig. 2:**
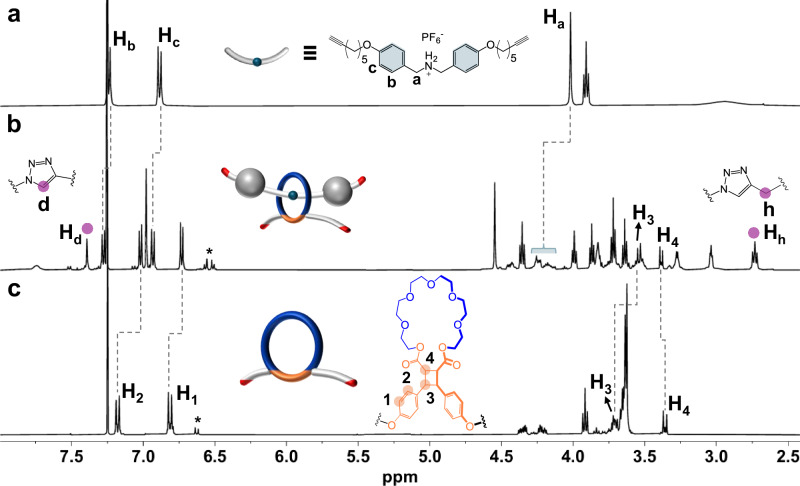
Structural characterization of G, RM and MCM. ^1^H NMR spectra of compound G **a** (500 MHz, CHCl_3_-*d*, 298 K), RM **b** (500 MHz, CHCl_3_-*d*, 298 K), and MCM (**c**) (400 MHz, CHCl_3_-*d*, 298 K), asterisk refers to the macrocycle with different cyclobutane stereochemistry (see in Supplementary Figs. 8–12). * represents isomer.

The ^1^H, ^13^C, HSQC, HMBC, and COSY measurements were conducted to analyze the structure of RM. According to ^1^H NMR spectra, after rotaxanation, the aromatic protons H_1_ and H_2_ of macrocycle MCM moved upfield, whereas the aromatic protons H_c_ adjacent to the DBA^+^ showed a significant downfield shift due to the complexation between axle’s cation and the wheel’s glycol fragment (Fig. 2). In addition, the appearance of the 1,2,3-triazole proton (H_d_) also confirmed the success of stoppering reaction. The proton signals of RM were clearly assigned relying on the HSQC, HMBC, and COSY spectra (Supplementary Figs. 9–12). ESI-MS of RM showed an m/z value identical to the calculated value. Therefore, these results confirm the successful synthesis of RM as the rotaxane-type mechanophore.

**Fig. 3:**
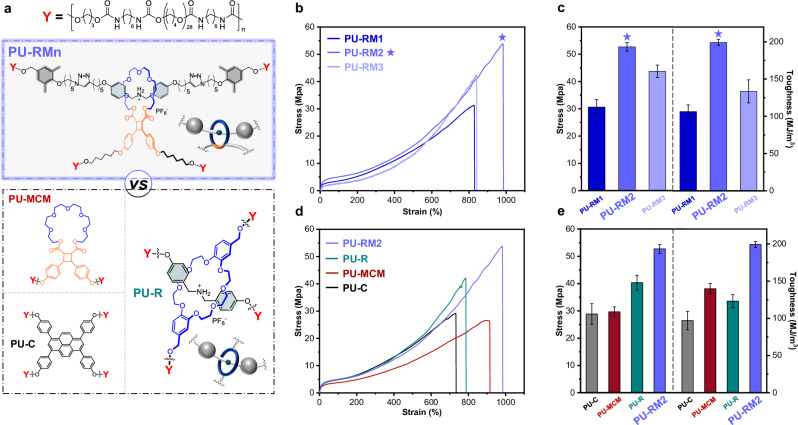
Mechanical properties of PU-RMn and controls. **a** Structures of PU-RMn and controls (PU-C, PU-MCM, and PU-R). **b** Stress–strain curves of PU-RMn. **c** Fracture stress and toughness of PU-RMn calculated from their stress-strain curves. **d** Stress–strain curves of PU-RM2 and controls. **e** Fracture stress and toughness of PU-RMn and controls calculated from their stress-strain curves. Values in (**c** and **e**) represent the mean and the standard deviation calculated from the mechanical property data obtained from three independent measurements.

With RM in hand, PU-RMn was obtained in high yield by polycondensation. Fourier transform infrared spectroscopy (FTIR) measurements showed the disappearance of the characteristic isocyanate band at 2270 cm⁻¹ along with the emergence of new carbonyl absorptions at around 1683 and 1722 cm⁻¹, confirming the occurrence of polycondensation (Supplementary Fig. 13). Additionally, in the ^1^H NMR spectrum (Supplementary Fig. 14), all proton signals of RM broadened after polymerization because of the restricted chain mobility, indicating successful incorporation of RM units into the polymer backbone. Linear polyurethane incorporated with MCM (PU-MCM), covalently crosslinked polyurethane (PU-C), and mechanophore-free rotaxane-crosslinked polyurethane (PU-R) were used as the control polymer samples, of which structures were also characterized by FTIR and ^1^H NMR measurements (Fig. 3a and Supplementary Figs. 15–18). ^1^H NMR spectra confirmed the accurate introduction and a similar molar ratio of key units in three polymers (compounds C in PU-C, MCM in PU-MCM, R in PU-R and RM in PU-RM2, respectively, see in Supplementary information). Equilibrium swelling measurements in THF showed a similar swelling ratio of the three crosslinked samples, indicating comparable effective network densities among them (Supplementary Fig. 19). Subsequently, differential scanning calorimetry (DSC) measurements were conducted to analyze the network construction of three polyurethane samples (Supplementary Fig. 20). PU-MCM with a linear structure showed the lowest glass transition (*T*_g_). The *T*_g_ values of PU-R and PU-RM were determined to be lower than that of PU-C, which may be due to the higher chain mobility of rotaxane-crosslinked polymer endowed by MINs^44,45^.

### Mechanical properties of PU-RMn and controls

To investigate the effect of multiple-pathway energy dissipation on the mechanical performance of the elastomer, a series of mechanical performance tests was carried out. First, the tensile tests showed PU-RM2 exhibited the highest fracture stress (53 MPa) and toughness (200 MJ/m³), indicating the best mechanical properties among PU-RMn and indicating an efficient energy-dissipation effect of the incorporated structure (Fig. 3b, c). Accordingly, PU-RM2 was selected for mechanistic investigation in the subsequent studies.

Subsequently, the mechanical performance of the three controls was evaluated by tensile tests and compared with PU-RM2 (Fig. 3d, e). In PU-MCM, cleavage of the macrocycle-type mechanophore dissipates energy and releases hidden length^20–25,46–48^, yet the absence of rotaxane crosslinks, leads to earlier tensile failure than PU-RM2. In PU-C, rigid covalent crosslinks provide resistance to deformation, but the lack of mobility imparted by rotaxane crosslinks and the sacrificial scission of mechanophore leads to reduced elasticity. Rotaxane-crosslinked PU-R exhibits higher tensile strength but reduced elongation compared to linear PU-MCM, consistent with the presence of a network structure. Although the mobility of rotaxane crosslinking nodes improves deformability relative to purely covalent networks, rotaxane sliding alone does not restore the elongation to the level of the linear sample, which can release hidden length. It is notable as well that the toughness of linear PU-MCM is slightly higher than that of rotaxane-crosslinked PU-R. This result indicates the significant contribution of covalent bond scission to the enhancement of elastomer toughness^49^. Meanwhile, Otsuka and co-workers^33^ demonstrated that when the sizes of the stopper and the macrocycle in rotaxane crosslinkers are properly matched, force-induced dethreading of the axle can provide an energy dissipation capacity comparable to that of selective mechanophore scission. Moreover, when mechanophores are incorporated as linkers bridging two non-dethreadable rotaxane units to form a bis-[2]rotaxane crosslinker, rotaxane sliding does not effectively synergize with mechanophore scission, and the resulting toughness is even lower than that of systems in which either dissipation pathway is present alone. In our work, PU-RM2 exhibits significantly higher toughness than both PU-R and PU-MCM (by 63% and 43%, respectively), indicating that both rotaxane sliding and selective mechanophore scission synergistically contribute to the substantial enhancement in toughness under mechanical stress. These results suggest that the mechanical bond can serve a dual role in systems where rotaxane and mechanophore motifs coexist. When the stopper size permits force-induced dethreading, the rotaxane acts as a mechanochemical protecting group for mechanophores. When the mechanophore is incorporated into the macrocyclic framework, and dethreading is inhibited due to oversized stoppers, a sequential dissipation process involving rotaxane sliding followed by mechanophore activation enables the synergistic contribution of both energy dissipation pathways to elastomer toughening. In addition, due to the absence of a control sample containing only the woven network, the specific contribution of the woven network converting from MINs, to the mechanical performance of PU-RM2 remains to be elucidated. Although the role of entanglements in fracture properties is expected to be different from that in elasticity and rheology^26^, we demonstrate that the woven network contributes to maintaining network integrity and dissipating energy in PU-RM2 by rheological analysis of different materials (vide infra).

### Sequential activation of multiple energy dissipation pathways

To more deeply investigate the multi-pathway energy dissipation in PU-RM2, we performed a series of mechanical, rheological, and spectroscopic tests. Synergistic analyses based on cyclic tensile tests, dynamic mechanical analysis (DMA), and FTIR measurements revealed that the three pathways are activated sequentially under stress.

**Fig. 4:**
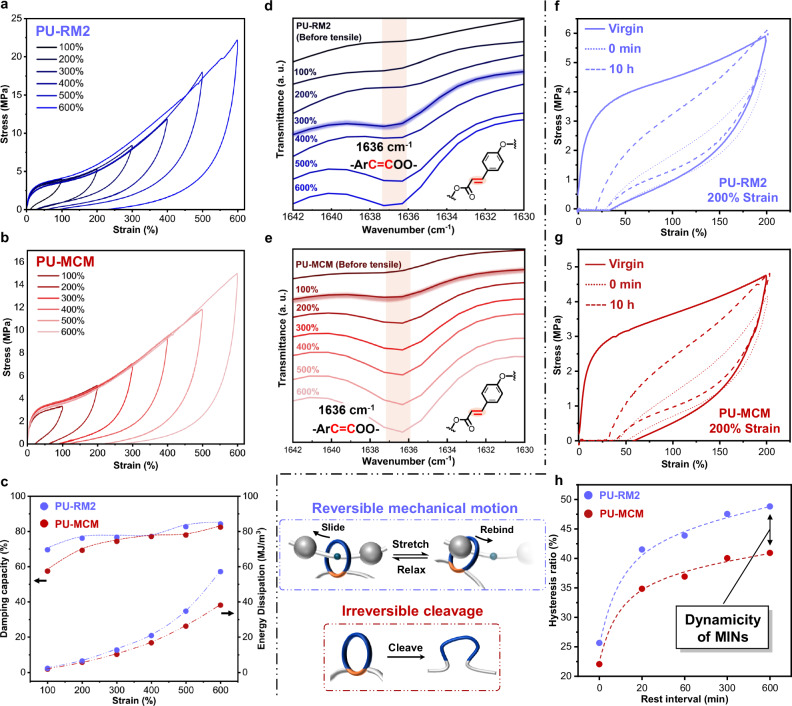
Sequential activation from slide motion of rotaxane crosslinkers to sacrificial mechanochemical scission. **a** Cyclic tensile curves of PU-RM2 recorded at room temperature with increased maximum strain. **b** Cyclic tensile curves of PU-MCM recorded at room temperature with increased maximum strain. **c** Damping capacity (the ratio of dissipated energy to total input energy) and hysteresis area of PU-RM2 and PU-MCM for each cycle in the cyclic tensile tests. **d** Partial FTIR spectra of PU-RM2 after tensile with a stain interval. **e** Partial FTIR spectra of PU-MCM after tensile with stain interval. **f**, **g** Cyclic tensile test curves of PU-RM2, and PU-MCM loaded at a strain of 200% with rest intervals from 0 to 600 min, respectively. (**h**) Hysteresis ratio for each cycle of the tensile tests in (**f**) and (**g**).

First, cyclic tensile tests for PU-RM2 and PU-MCM over a strain range of 100–600 % were conducted (Fig. 4a–c and Supplementary Figs. 23 and 24). The damping capacity, defined as the ratio of energy dissipation to incoming energy, was high for both PU-RM2 and PU-MCM. Moreover, PU-RM2 exhibited energy dissipation comparable to PU-MCM at low strain but increased more rapidly with increasing strain, suggesting concentrated activation of dissipation pathways for PU-RM2 at high strains.

Subsequently, FTIR measurements of PU-RM2 and PU-MCM samples after cyclic tensile tests were conducted to investigate the molecular structure evolution during stretch. In the spectra collected before loading, PU-RM2 showed no detectable C=C stretching band assigned to cinnamate moieties at 1636 cm⁻¹, indicating an intact rotaxane structure in PU-RM2 (Fig. 4d and Supplementary Fig. 28)^50^. With exerting stress during tensile, the C=C band remained essentially absent when the strain is lower than 300%, but emerged and increased sharply at higher strains. This result indicates, at low load, the rotaxane crosslinker in PU-RM2 remains intact, and energy dissipation arises primarily from macrocycle sliding along the axle. As the tensile proceeds into the high load and the slide motion of rotaxane reaches its maximum, the truxinate group in macrocycle sacrificially cleaves to cinnamate structure via force-induced retro-[2 + 2] cycloaddition, thereby taking over energy dissipation as the secondary pathway^51,52^. In contrast, stretching PU-MCM led to emergence of C=C stretching band at the beginning stage of tensile, with gradually increased intensity (Fig. 4e). Without mechanical motion of rotaxane serving as the first energy dissipation pathway, the macrocycle-type mechanophore (MCM) in PU-MCM undergoes early mechanochemical scission upon stress loading. These results prove that the PU-RM2 crosslinked by rotaxane-type mechanophore exhibits sequential activation of energy dissipation pathways (MINs versus mechanochemical scission): sliding of components in MINs at low load, followed by sacrificial scission of the mechanophore at higher load.

To further investigate the effect of MINs’ dynamicity on mechanical performances, recovery experiments using cyclic tensile tests at a strain of 200% were conducted (Fig. 4f and g). The results showed PU-RM2 exhibited superior recovery compared with PU-MCM. This arises because, at this low load (200%), irreversible mechanochemical scission in PU-RM2 is deferred by the mechanical motion of the rotaxane, thereby dissipating energy through a reversible structural evolution (e.g., sliding). These results further indicate that, prior to activation of the RM mechanophore, the dynamicity of the rotaxane already contributes to energy dissipation and renders the recovery property. Additionally, the tensile experiments with different deformation rates (from 30 to 400 mm min^-1^) revealed the distinct energy dissipation behavior of PU-RM2 and PU-MCM. The energy dissipation of PU-MCM at 600% strain shows nearly no dependency on deformation rate, indicating that the mechanophore activation is primarily governed by the strain amplitude when it serves as the only energy dissipation pathway (Supplementary Figs. 31 and 32). In contrast, the energy dissipation of PU-RM2 increases as the deformation rate rises from 30 to 200 mm min⁻¹, and then decreases at higher deformation rates (Supplementary Figs. 33 and 34). This non-monotonic trend originated from the coupling between rotaxane’s dynamicity and activation of mechanophore: at low deformation rates, with sufficient relaxation, the rotaxane motifs undergo reversible rearrangements, which allows the rebind of macrocycle to the station on axle. Therefore, the activation of mechanophore was prevented, resulting in limited energy dissipation. As the deformation rate increases to an intermediate regime (200 mm min⁻¹), stress can no longer be fully released through rotaxane rebinding, and thereby effectively drives the sequential activation of rotaxane sliding followed by mechanophore scission, leading to a maximum energy dissipation. At high deformation rates, the rapid loading bypasses rotaxane sliding that should have been the first dissipation pathway, resulting in direct activation of mechanophore or even failure of local segments in a more quasi-brittle manner. This non-monotonic rate dependence highlights a time-scale matching window where the topological energy dissipation architecture of PU-RM2 is most effectively utilized.

**Fig. 5:**
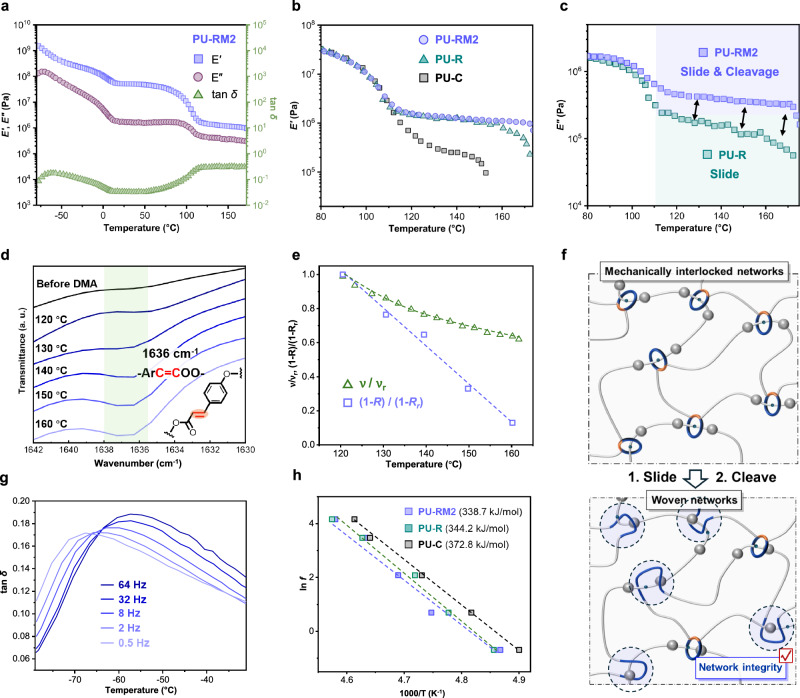
Preservation of network integrity by inherently formed woven networks. **a** Dynamic mechanical analysis of PU-RM2 by temperature sweep. **b** Storage modulus (*E*′) curves of PU-RM2, PU-R, and PU-C in the temperature range from 80 °C to 170 °C. **c** Loss modulus (*E*″) curves of PU-RM2 and PU-R in the temperature range from 80 °C to 170 °C. **d** Partial FTIR spectra of PU-RM2 measured at different temperatures after DMA (120, 130, 140, 150, and 160 °C, respectively). **e** Plot of *ν*/*ν*_*r*_ or (1-*R*)/(1-*R*_*r*_) versus DMA temperature, where *ν* refers to the network density, *ν*_*r*_ refers to network density at 120 °C in DMA, *R* refers to rupture ratio of cyclobutane (see Supplementary Figs. 38–42), *R*_*r*_ refers to rupture ratio of cyclobutane at 120 °C in DMA. **f** Schematic presentation of the formation of woven networks along with the rupture of rotaxane crosslink. **g** Temperature sweeps of PU-RM2 under different frequencies using DMA. The transition occurs at approximately −80 to −30 °C. **h** Arrhenius plots of transitions for PU-RM2, PU-R, and PU-C based on the frequency-dependent shifts of the tan *δ* peaks, respectively.

DMA was conducted to further investigate the role of woven networks converted from rupture of rotaxane crosslinkers. According to DMA results, significant differences in mechanical behaviors of PU-RM2 and controls are observed, especially after dissociation of hydrogen bonds in polyurethane backbones (Fig. 5a and Supplementary Figs. 35–37). The linear PU-MCM, which lacks a crosslinked network, lost resistance to chain flow and showed a steep drop in both storage modulus (*E*′) and loss modulus (*E*″) between 70–100 °C (Supplementary Fig. 35). FTIR measurement of PU-MCM after DMA showed no appearance of cinnamate bands, indicating that the macrocycle-type mechanophores are not activated and that failure arises from hydrogen-bond dissociation at elevated temperature (Supplementary Fig. 35). For PU-C, heat-triggered dissociation of hydrogen bond likewise causes sharp decreases in *E*′ and *E*″, yet the existence of covalent crosslink networks results in a more gradual decline in the elastic plateau (115–145 °C) (Supplementary Fig. 36). Within this plateau, *E*′ and *E*″ gradually decreased with increasing temperature, likely due to the earlier rupture of shorter PU chains in PU-C compared with those in the PU containing MINs^30^. In contrast, the moduli of the rotaxane-crosslinked PU-R and PU-RM2 enter an elastic plateau region, where the decrease becomes much more gradual as the temperature increases to 110 °C. In this region, the *E*′ values of PU-R and PU-RM2 are comparable and remain higher than those of PU-C. This result indicates that the mobility of the rotaxane crosslinking nodes effectively dissipates energy, thereby preventing premature rupture in chemical structures. In addition, the decrease in *E*′ at around 160°C indicates the loss of network stability in PU-R due to the limited energy dissipation capacity of the MINs under such conditions. In contrast, PU-RM2 shows no obvious loss of network elasticity until 175 °C (Fig. 5b). The loss modulus *E*″ of PU-RM2 is remarkably higher than that of PU-R between 110 °C and 175 °C (Fig. 5c). These results suggest that although both MIN-containing samples possess comparable network elasticity, PU-RM2 exhibits prolonged network integrity and more effective energy dissipation within the elastic plateau region. This enhanced dissipation and network stability may arise from sacrificial mechanophore cleavage and the subsequent formation of woven networks, as supported by FTIR measurements. In this plateau region, FTIR of PU-RM2 detects sustained cleavage of the truxinate moiety, indicating the gradual break of MINs (Fig. 5d). The rupture ratio of the truxinate mechanophore in the rotaxane crosslinker was estimated by FTIR by using the methylene (CH₂) scissoring deformation band as an internal standard, which shows a rupture ratio (*R*) of 15% at the beginning (*R*_120 °C_), 45% in the middle (*R*_140 °C_), and 89% near the terminal of plateau (*R*_160 °C_) (Fig. 5d and Supplementary Figs. 38–42). From the classical rubber elasticity equation (${E}^{{\prime} }=3\nu {k}_{{{\rm{B}}}}T$), normalizing the storage modulus (*E*′) by the value at a reference temperature of 120 °C gives

1 $$\frac{E^{\prime} {T}^{-1}}{{E^{\prime} }_{r}{T}_{r}^{-1}}=\frac{\nu }{{\nu }_{r}}$$

*ν*/*ν*_r_ reflects the decrease in network density since 120°C, and should therefore match the normalized value of the remaining crosslinker content, namely *ν*/*νr*=(1-*R*)/(1-*Rr*). However, DMA and FTIR results show a remarkable tendency of *ν*/*ν*_*r*_ > (1-*R*)/(1-*R*_*r*_) with increasing temperature, and a significant gap remains even when 89% of the rotaxane crosslinkers have ruptured at 160 °C (Fig. 5e). This indicates that the structures concurrently formed with the failure of rotaxane crosslinking could compensate for the *E*′. We attribute this contribution to woven networks that are sequentially activated upon rotaxane scission: once the rotaxane crosslinks fail, the threaded and interlaced chain segments persist as topological junctions to form the woven networks, thereby preserving the topology consistency and network integrity (Fig. 5f)^29,39,43^.

Furthermore, multi-frequency temperature sweep tests of the PU-RM2 (Fig. 5g) and the controls were carried out in the temperatures ranging from -80 to -30 °C to explore the activation energy associated with the glass transition in the polymer networks. The activation energy of the glass transition for crosslinked samples PU-C, PU-R, and PU-RM2 were calculated to be 372.8, 344.2, and 338.7 kJ/mol, respectively (Fig. 5h). The ordering PU-C > PU-RM2 ≈ PU-MCM reflects their network architectures: PU-C has the most rigid covalent crosslinks; PU-R and PU-RM2 possesses MINs with higher chain mobility^8–10,44,45^.

In concert, mechanical tests and spectroscopic measurements synergistically reveal the establishment of a sequentially activated, and multi-pathway energy dissipation mechanism in elastomer preparation via the utilization of a mechanophore-bearing rotaxane crosslinker.

## Discussion

We propose a sequentially activated, multi-pathway (ternary) energy-dissipation mechanism and achieve its materialization in a polyurethane elastomer, demonstrating its toughening effect. Under external stress, the three pathways sequentially activate as: (1) For primary pathway, the synthesized rotaxane crosslinker bearing a truxinate mechanophore in the wheel is incorporated into the polymer to construct MINs, which dissipate energy via sliding of the rotaxane components. (2) For secondary pathway, as sliding reaches its maximum and the load increases, the mechanophore is activated to undergo sacrificial cleavage to take over the energy dissipation. (3) For tertiary pathway, rupture of the rotaxane crosslinks inherently construct MINs into woven networks that act as physical crosslinks, preserve network integrity, and continue to dissipate energy by chain slipping and tension arrangement. This sequential activation mechanism enables the engagement of each dissipation pathway, achieving synergistic toughening of rotaxane-based MINs and selective mechanophore scission. The toughness of the polyurethane incorporating this multi-pathway energy dissipation was markedly enhanced compared with systems featuring only a single energy dissipation pathway (either the scissile mechanophore, rotaxane crosslinking, or the covalent crosslinking). This work may provide insight for synergistically combining multiple energy dissipation pathways to toughen polymeric materials.

## Methods

### Materials

All solvents for synthesis and reactions were purchased from TCI. All other chemicals were purchased from TCI and Nacalai Tesque and used without further purification.

### Preparation of PU-RM and controls

**RM** (molar ratio 1.0 %, 2.5 %, 4.0%), Poly(tetrahydrofuran) (PTMG, *M*_*n*_ = 2000, 2.00 g, 1.00 mmol) dissolved in dry CH_2_Cl_2_ (25 mL). 1,6-Diisocyanatohexane (HDI, 0.36 g, 2.13 mmol) and Dibutyltin diacetate (DBTDA, 2.50 mg) in dry CH_2_Cl_2_ (5 mL) were added to the mixture under N_2_ atmosphere. The mixture was stirred at room temperature for 2 h. 1,3-Propanediol (POD, 76.0 mg, 1.00 mmol) dissolved in dry CH_2_Cl_2_ (2 mL) was added into the mixture followed by further stirring for 22 h. The reaction mixture was added to hexane to precipitate the polymer. The collected precipitate was dissolved in CH_2_Cl_2_ and poured into a Teflon mold and dried at 25 °C overnight to afford the **PU-RM1**-**3** film (yield from 86% to 95%). The controls were prepared through the same procedure and the monomer feedings for each sample were seen in Supplementary Information.

### Materials characterizations

The ^1^H, ^13^C NMR spectra were obtained using a JEOL JNM-ECS 400 and 500 MHz NMR spectrometer, and Agilent VNS 600 MHz NMR spectrometer. All ROESY spectra were obtained using an Agilent VNS 600 MHz NMR spectrometer. All NMR spectra were processed by MestReNova software. ESI-MS spectra were recorded on Bruker micrOTOF-QII spectrometers. The UV irradiation was performed by using PER-448, Techno Sigma Co., Ltd. The Fourier transform infrared (FTIR) spectra were measured using JASCO FTIR-6100 spectrometer. Tensile tests of the elastomers were performed using Autograph AG-X plus (Shimadzu Co.). Young’s modulus was calculated from initial slope of stress-strain curve at a range between 1% -10% strain. Cyclic tensile tests were performed using Autograph AG-X plus (Shimadzu Co.). Test pieces were continuously stretched and recovered without interval. Maximum strains were set to 100%, 200%, 300%, 400%, 500% and 600% at deformation rate of 300 mm/min. Glass transition temperatures (T_g_) of the polymers were measured by a DSC system (Hitachi High-Tech NEXTA DSC200) with N_2_ gas flow (30 mL/min.). Thermal transitions were measured in the temperature range of -150–0 °C heated 10 °C/min. (the first scanning). After the first scanning, the samples were cooled to -150 °C and reheated to 0 °C (the second scanning). Dynamic mechanical analysis (DMA): The viscoelastic properties were measured by DMA system using DVA-250 (IT-Keisoku Seigy Co., Ltd.).

## Supplementary information

Supplementary Information

Transparent Peer Review file

## Source data

Source Data

## Data Availability

All data are available from the corresponding author upon request. The data supporting the results in this study are available within the main text and the Supplementary Information. Source data are provided. Source data are provided with this paper.
