# Supplementary material for: Toughening elastomer via sequentially activated multi-pathway energy dissipation
Source: Nat Commun. 2026 Jul 1;17:5452. doi: 10.1038/s41467-026-74148-z (PMC13323342; doi:10.1038/s41467-026-74148-z)
Supplement: Supplementary file 1 — Supplementary Information [file 41467_2026_74148_MOESM1_ESM.pdf]

Supplementary Information for

Toughening Elastomer via Sequentially Activated Multi-Pathway Energy Dissipation

Xue Li[1], Chunlin Xiao[1*], Haruki Izutsu[1], Osamu Urakawa[1,2], Tadashi Inoue[1,2], Yuichiro Kobayashi[1,2,3*], Hiroyasu Yamaguchi[1,2,3*]

[1]Department of Macromolecular Science, Graduate School of Science, The University of Osaka, Toyonaka, Osaka 560-0043, Japan

[2]Forefront Research Center, Graduate School of Science, The University of Osaka, Toyonaka, Osaka 560-0043, Japan

[3]Innovative Catalysis Science Division, Institute for Open and Transdisciplinary Research Initiatives (ICS-OTRI); The University of Osaka, Suita, Osaka 565-0871, Japan

*Corresponding authors' Email: xiaoc20@chem.sci.osaka-u.ac.jp;

kobayashiy11@chem.sci.osaka-u.ac.jp; hiroyasu@chem.sci.osaka-u.ac.jp

Table of contents

1. Synthesis of compounds S3
2. Preparation of polymers S52
3. Structural analysis of RM S57
4. Structural characterization of polymers S60
5. Mechanical properties of PU-RMn and controls S65
6. Rupture ratio evaluation based on FTIR measurements S75
7. References S79

1. Synthesis of compounds

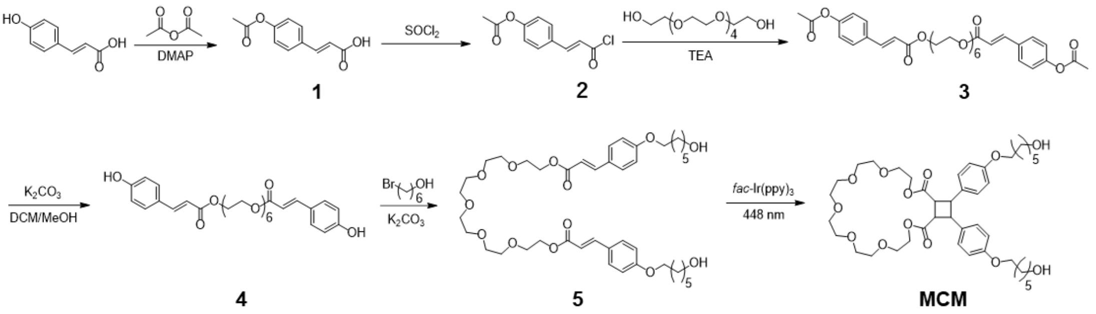

Supplementary Figure 1. Synthetic route of MCM.

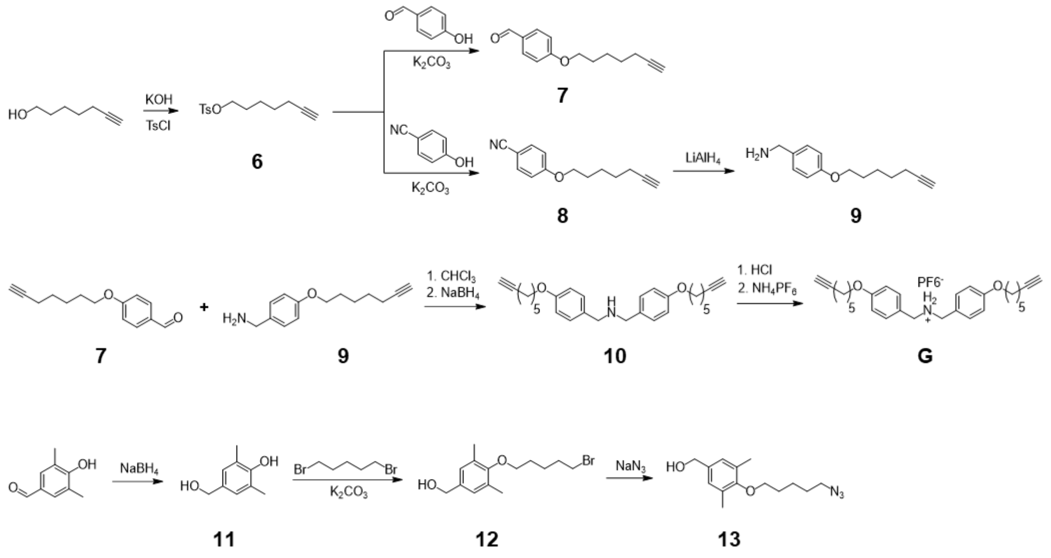

Supplementary Figure 2. Synthetic route of axle molecules.

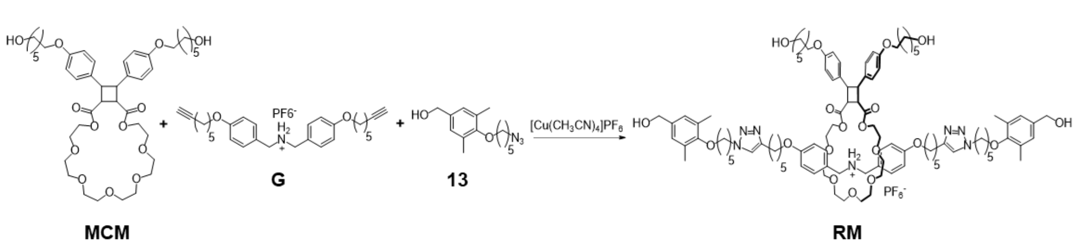

Supplementary Figure 3. Synthetic route of RM.

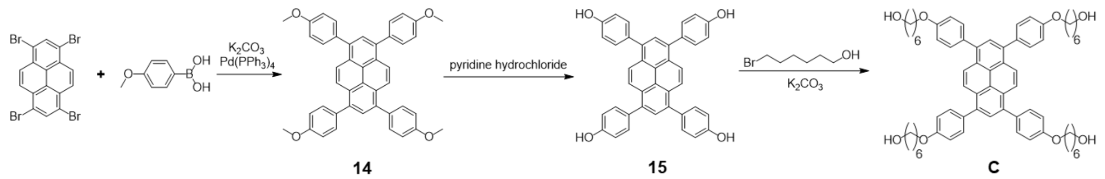

Supplementary Figure 4. Synthetic route of C.

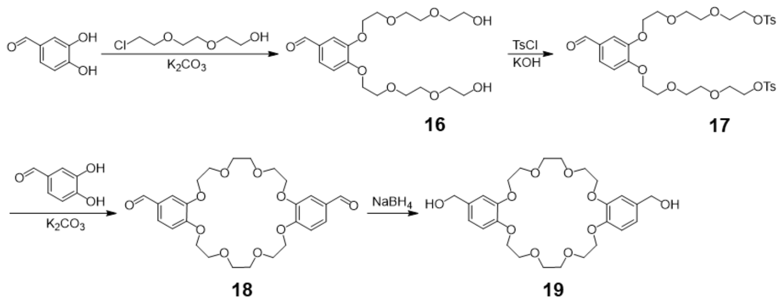

Supplementary Figure 5. Synthetic route of 19.

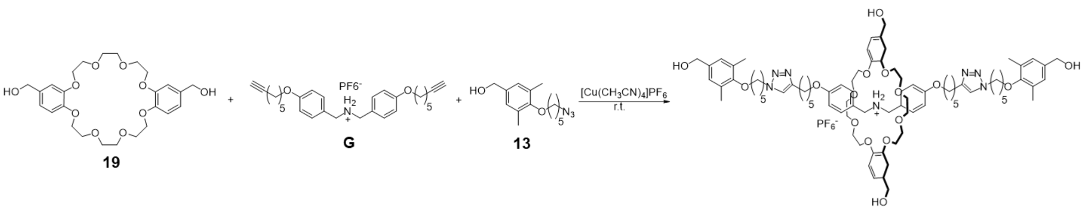

Supplementary Figure 6. Synthetic route of R.

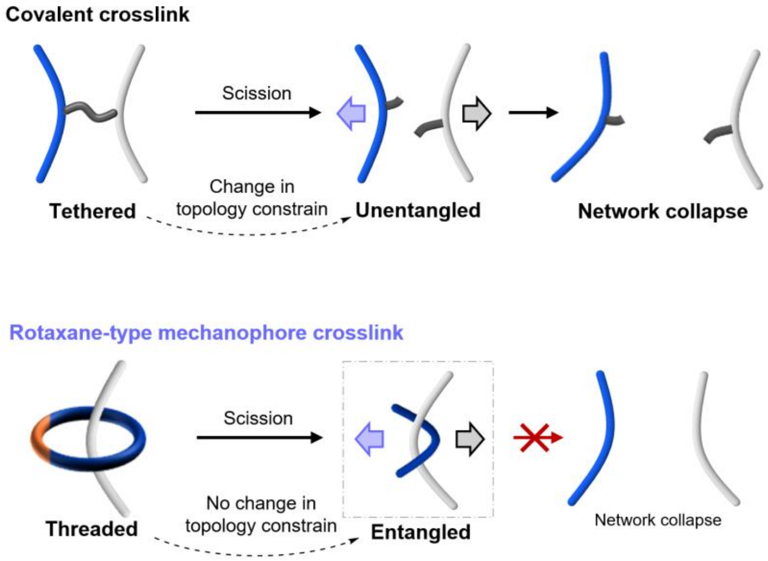

Supplementary Figure 7. Transformation from rotaxane structure to entangled chains.

Synthesis of 1

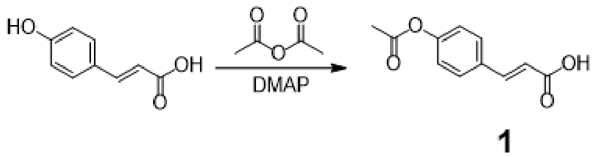

1

To a solution of DMAP (93.00 mg, 0.76 mmol) and acetic anhydride (4.28 mL, 46.26 mmol) in dry pyridine (10 mL) was added *trans*-*p*-coumaric acid (5.00 g, 30.46 mmol) in an ice bath. The ice bath was removed and the mixture was stirred at room temperature for 1 hour and then poured into crushed ice. A white solid formed when the solution was acidified (pH = 2) and was collected via suction filtration. 1 was obtained as a white solid (Yield: 6.05 g, 97%).^{1}H NMR (500 MHz, $CHCl_3$-*d*) δ 7.76 (d, *J* = 15.9 Hz, 1H), 7.56 (d, 2H), 7.14 (d, *J* = 8.6 Hz, 2H), 6.40 (d, *J* = 16.0 Hz, 1H), 2.31 (s, 3H).

Data was consistent with those reported in the literature [1].

^{1}H NMR (500 MHz, $CHCl_3$-*d*, 298 K) spectrum of 1

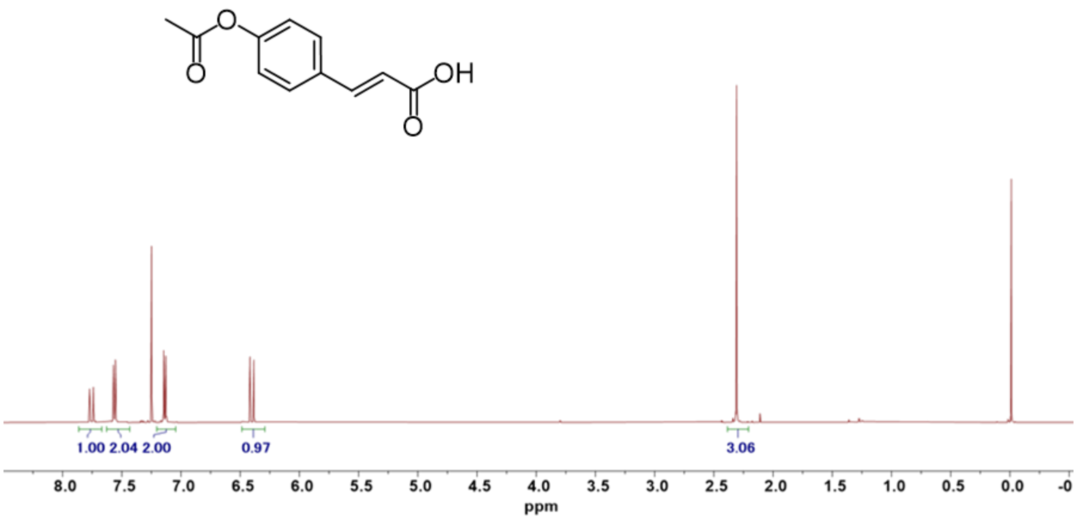

Synthesis of 2

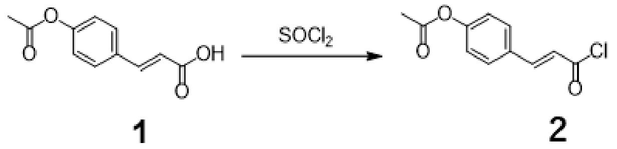

Thionyl chloride ($SOCl_2$) (4.57 mL, 63.02 mmol) and three drops of DMF were added into the dispersion of 1 (5.00 g, 24.24 mmol) in dry $CHCl_3$ (40 mL). The mixture was refluxed for 3 hours and then the solvent was evaporated under reduced pressure. 2 was obtained as a red solid (Yield: 5.01 g, 92%). ^{1}H NMR (500 MHz, $CHCl_3$-*d*) δ 7.80 (d, *J* = 15.5 Hz, 2H), 7.58 (d, *J* = 8.7 Hz, 4H), 7.17 (d, *J* = 8.7 Hz, 4H), 6.59 (d, *J* = 15.6 Hz, 2H), 2.31 (s, 6H).

Data was consistent with those reported in the literature [1].

^{1}H NMR (500 MHz, $CHCl_3$-*d*, 298 K) spectrum of 2

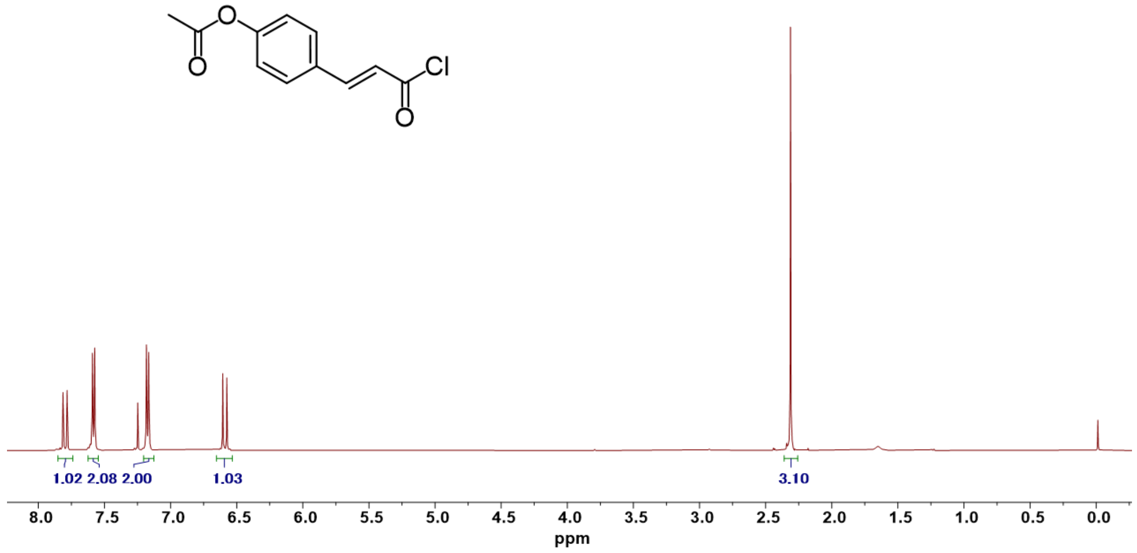

Synthesis of 3

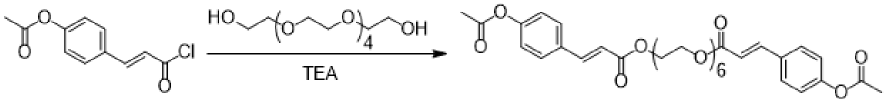

2

3

To a solution of hexaethylene glycol (3.00 g, 10.63 mmol) and TEA (3.47 mL, 24.44 mmol) in dry DCM (100 mL) was dropwise added the solution of 2 (5.49 g, 24.44 mmol) in dry DCM (50 mL). The mixture was stirred overnight and quenched by addition of 1 M HCl. The organic phase was washed with saturated $NaHCO_3$ solution (1x), brine (1x), dried over $MgSO_4$ and evaporated. The crude 3 was obtained directly used for next step.

Data was consistent with those reported in the literature [1].

Synthesis of 4

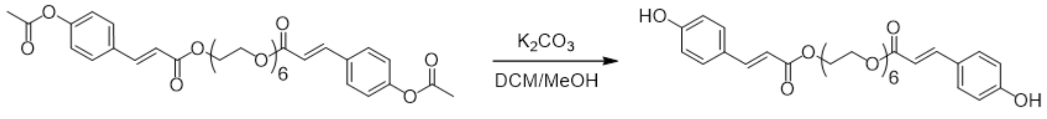

3 **4**

The crude 3(7.00 g, 10.63 mmol) was dissolved in 60 mL mixture solvent of DCM and MeOH (v/v = 1/1). K_2CO_3 (3.37 g, 23.38 mmol) was added into the solution and stirred for 30 minutes at room temperature. The reaction mixture was filtered, and the filtrate was collected and evaporated. The crude was purified via silica column chromatography (DCM/MeOH). 25 was obtained as a light-yellow oil (4.95 g, 81%).^{1}H NMR (500 MHz, $CHCl_3$-*d*) δ 7.54 (d, *J* = 15.9 Hz, 2H), 7.34 – 7.27 (d, 4H), 6.82 (d, *J* = 8.6 Hz, 4H), 6.14 (d, *J* = 15.9 Hz, 2H), 4.39 – 4.24 (m, 4H), 3.79 – 3.71 (m, 4H), 3.69 – 3.63 (m, 4H), 3.63 – 3.61 (m, 4H), 3.60 – 3.52 (m, 8H).

Data was consistent with those reported in the literature [1].

^{1}H NMR (500 MHz, $CHCl_3$-*d*, 298 K) spectrum of 4

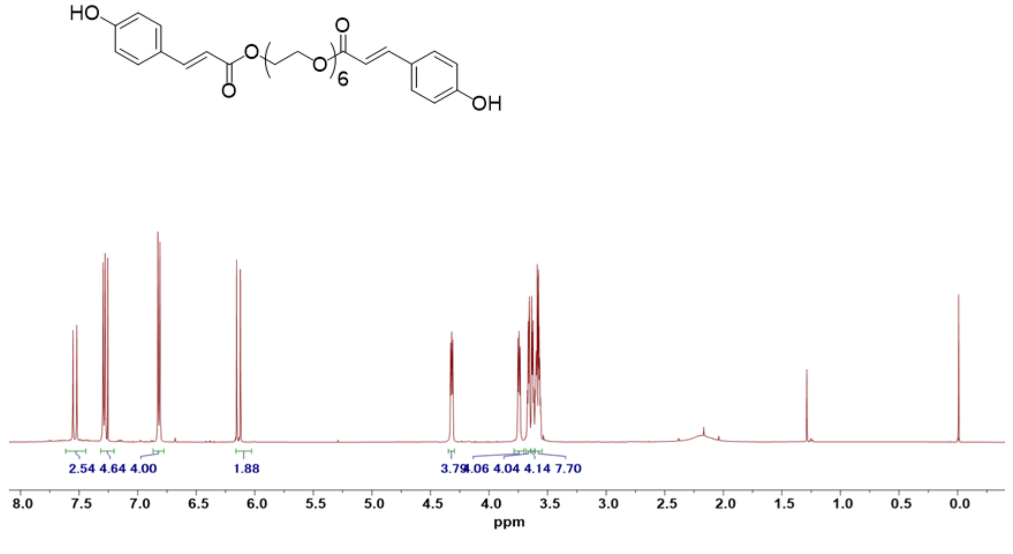

Synthesis of 5

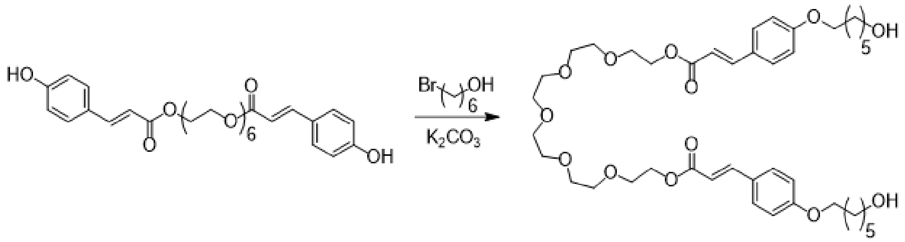

4 5

To a solution of 4 (2.43 g, 4.23 mmol) and 6-bromohexan-1-ol (1.57 g, 8.67 mmol) in DMF (30 mL) was added K_2CO_3 (2.92 g, 21.14 mmol) under N_2 atmosphere. After stirring at 70 °C overnight, the reaction mixture was poured into water and extracted with ethyl acetate. The organic phase was washed with water (3 x) and brine (1x), dried over $MgSO_4$ and evaporated. 5 was obtained as orange oil (Yield: 3.10 g, 94%). ^{1}H NMR (500 MHz, $CHCl_3$-*d*) δ 7.63 (d, *J* = 15.9 Hz, 2H), 7.44 (d, *J* = 8.8 Hz, 4H), 6.86 (d, *J* = 8.8 Hz, 4H), 6.32 (d, *J* = 16.0 Hz, 2H), 4.35 – 4.31 (m, 4H), 3.97 (t, *J* = 6.5 Hz, 4H), 3.76 – 3.73 (m, 4H), 3.68 – 3.62 (m, 20H), 1.83-1.74 (m, 4H), 1.63-1.54 (m, 4H), 1.52 – 1.39 (m, 8H).^{13}C NMR (126 MHz, $CHCl_3$-*d*) δ 167.38, 161.05, 144.88, 129.84, 127.02, 115.27, 114.90, 70.72, 70.69, 70.67, 69.40, 68.06, 63.60, 62.94, 32.73, 29.20, 25.93, 25.60. HR-MS (ESI): calcd for , $[C_{42}H_{63}O_{13}+H]^+$, m/z = 775.4264, found m/z =775.4263.

^{1}H NMR (500 MHz, $CHCl_3$-*d*, 298 K) spectrum of 5

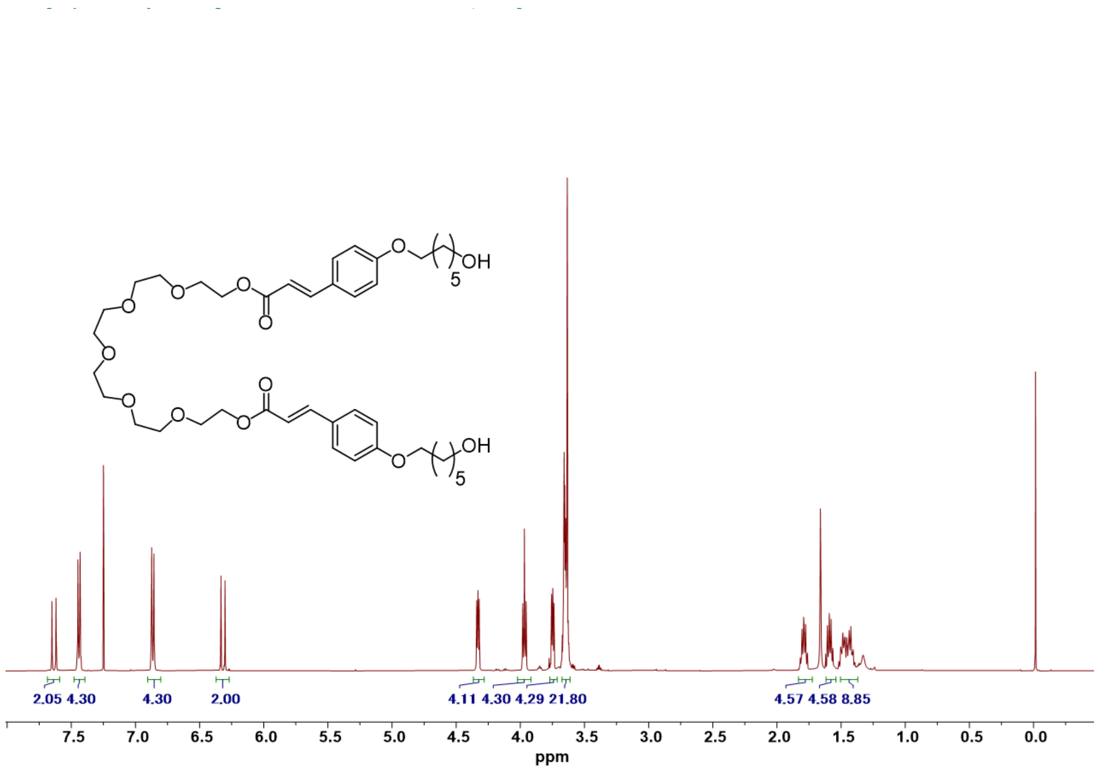

^{13}C NMR (126 MHz, $CHCl_3$-*d*, 298 K) spectrum of 5

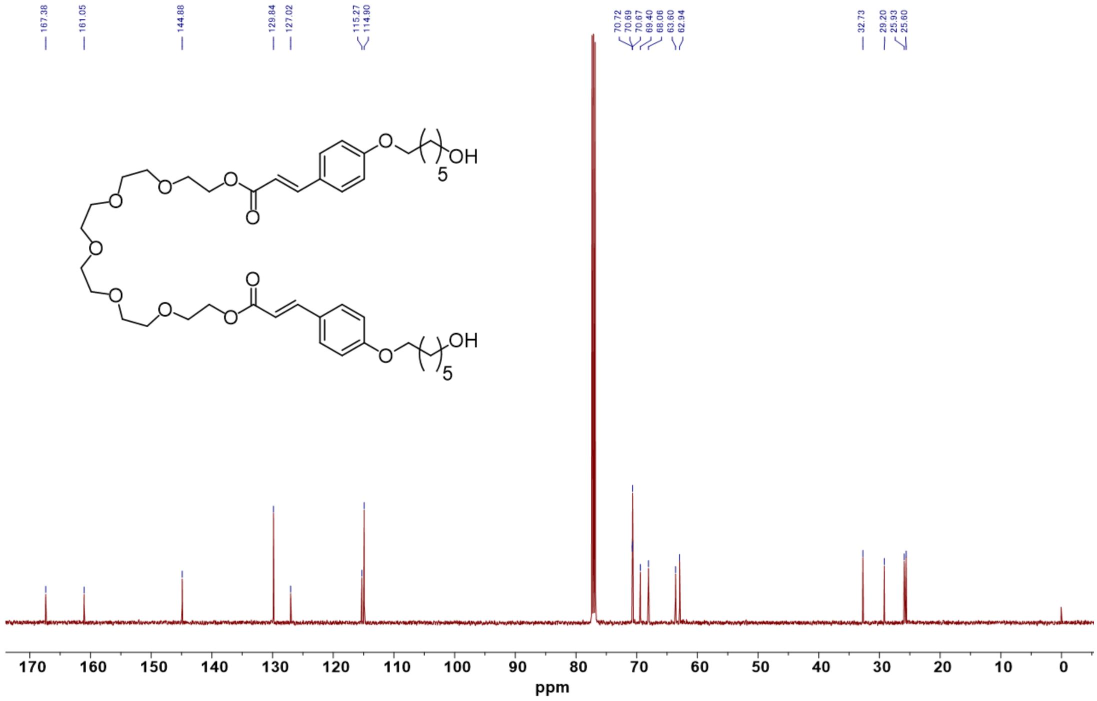

Synthesis of MCM

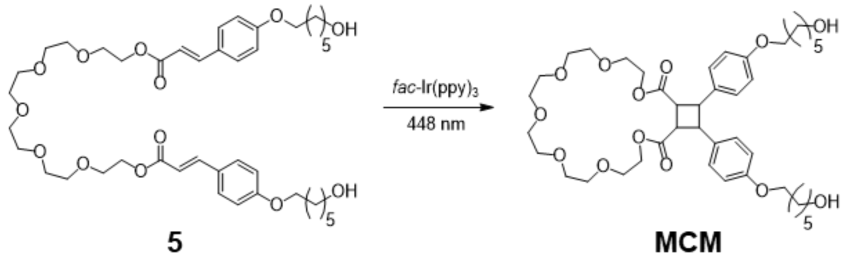

5 (3.50 g, 4.52 mmol) and tris(2-phenylpyridine)iridium (III) (*fac*-Ir(ppy)$_3$) (0.12 g, 0.18 mmol) was dissolved in 1,4-Dioxane (400 mL) and degassed by bubbling with N_2. The solution was irradiated with 448 nm UV light in a water bath for 3 days. After irradiation, the solution evaporated. The crude was purified via the silica column chromatography (CH_2Cl_2/ Methanol = 100/0 – 95/5, gradient elution). MCM was isolated as orange oil (Yield: 3.33 g, 95%).^{1}H NMR (400 MHz, $CHCl_3$-*d*) δ 7.18 (d, J = 8.7 Hz, 4H), 6.81 (d, J = 8.7 Hz, 4H), 4.38-4.17 (m, 4H), 3.91 (t, J = 6.5 Hz, 4H), 3.72 – 3.56 (m, 26H), 3.40 – 3.28 (m, 2H), 1.81 – 1.73 (m, 4H), 1.66 – 1.51 (m, 4H), 1.51 – 1.32 (m, 8H).^{13}C NMR (101 MHz, $CHCl_3$-*d*) δ 172.60, 158.22, 133.11, 128.03, 114.61, 71.03, 70.81, 69.19, 67.91, 64.58, 62.98, 46.61, 45.35, 32.76, 29.30, 25.97, 25.60. HR-MS (ESI): calcd for , $[C_{42}H_{63}O_{13}+H]^+$, m/z = 775.4264, found m/z =775.4267.

^1H NMR (400 MHz, $CHCl_3$-*d*, 298 K) spectrum of MCM

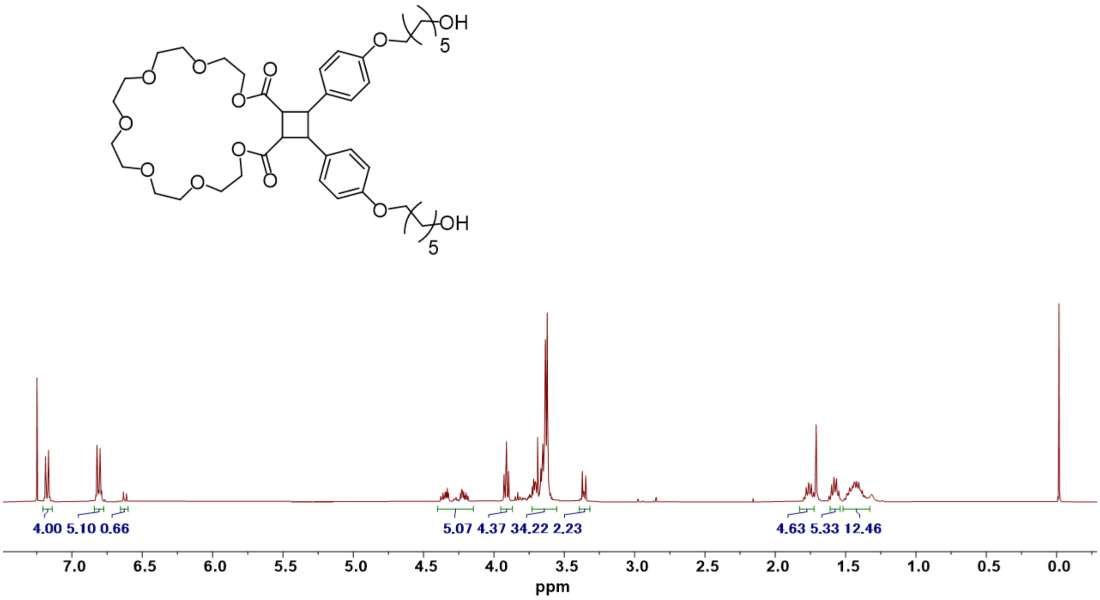

^{13}C NMR (101 MHz, $CHCl_3$-*d*, 298 K) spectrum of MCM

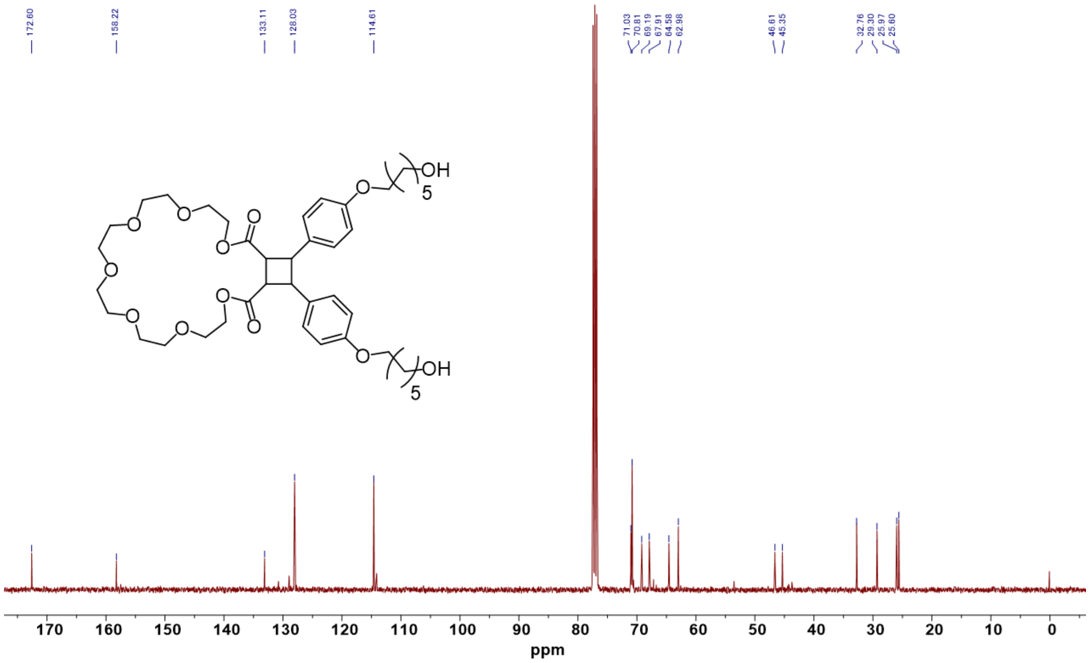

Synthesis of 6

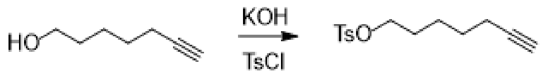

6

6-Heptyn-1-ol (4.00 g, 35.66 mmol) and p-toluenesulfonyl chloride (8.16 g, 42.8 mmol) were dissolved in diethyl ether (40 mL). KOH (11.2 g, 0.20 mol) was added portionwise to the solution under an ice bath, and the reaction was stirred for 3 h. Then the reaction mixture was poured into water and extracted with diethyl ether (2x). The organic phase was washed with water (2x) and brine (1x), dried over Na_2SO_4 and evaporated. The crude was purified via silica column chromatography (Hexane/Ethyl acetate). 6 was obtained as a colorless oil (Yield: 4.07 g, 86%).^{1}H NMR (500 MHz, $CHCl_3$-*d*) δ 7.77 (d, *J* = 8.3 Hz, 2H), 7.35 – 7.31 (d, *J* = 8.5 Hz, 2H), 4.01 (t, *J* = 6.5 Hz, 3H), 2.43 (s, 4H), 2.16-2.08 (m, 2H), 1.91 (t, *J* = 2.6 Hz, 1H), 1.68-1.60 (m, 2H), 1.49 – 1.36 (m, 4H).

Data was consistent with those reported in the literature [2].

^{1}H NMR (500 MHz, $CHCl_3$-*d*, 298 K) spectrum of 6

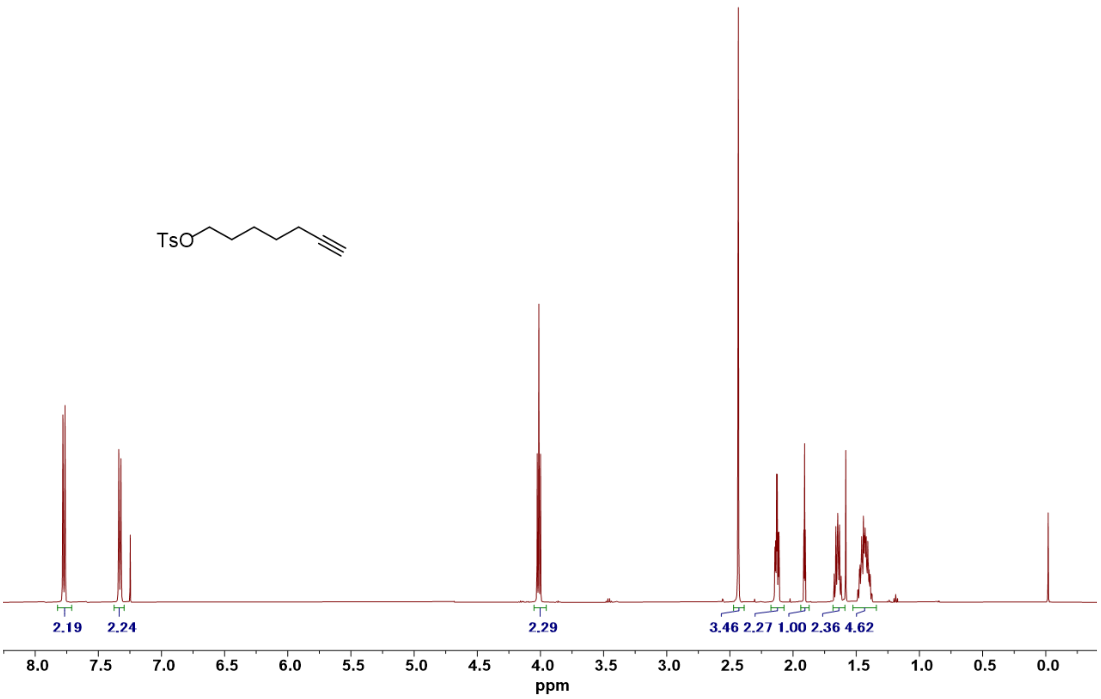

Synthesis of 7

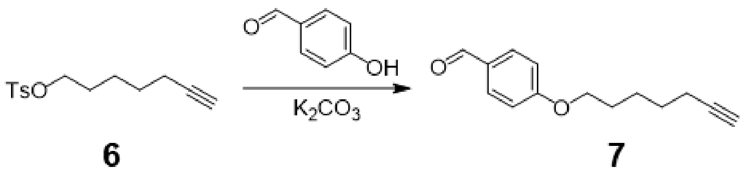

To a solution of 6 (3.00 g, 11.3 mmol) and 4-Hydroxybenzaldehyde (1.31 g, 10.7 mmol) in DMF (30 mL) was added K_2CO_3 (4.45 g, 32.2 mmol). After bubbling by N_2 for 10 minutes, the mixture was stirred at 70 °C overnight. The reaction mixture was poured into water and extracted with ethyl acetate. The organic phase was washed with water (2x) and brine (1x), dried over $MgSO_4$ and evaporated. 7 was obtained as a white solid (Yield: 2.16 g, 93%). ^{1}H NMR (500 MHz, $CHCl_3$-*d*) δ 9.86 (s, 1H), 7.81 (d, *J* = 8.8 Hz, 2H), 6.97 (d, *J* = 8.7 Hz, 2H), 4.03 (t, *J* = 6.4 Hz, 2H), 2.26 – 2.18 (m, 2H), 1.95 (t, *J* = 2.7 Hz, 1H), 1.87 – 1.78 (m, 2H), 1.63 – 1.54 (m, 4H). ^{13}C NMR (126 MHz, $CHCl_3$-*d*) δ 190.95, 164.22, 132.09, 129.86, 114.81, 84.31, 68.59, 68.22, 28.66, 28.19, 25.23, 18.43. HR-MS (ESI): calcd for , $[C_{14}H_{17}O_2+H]^+$, m/z =217.1224, found m/z = 217.1223.

^{1}H NMR (500 MHz, $CHCl_3$-*d*, 298 K) spectrum of 7

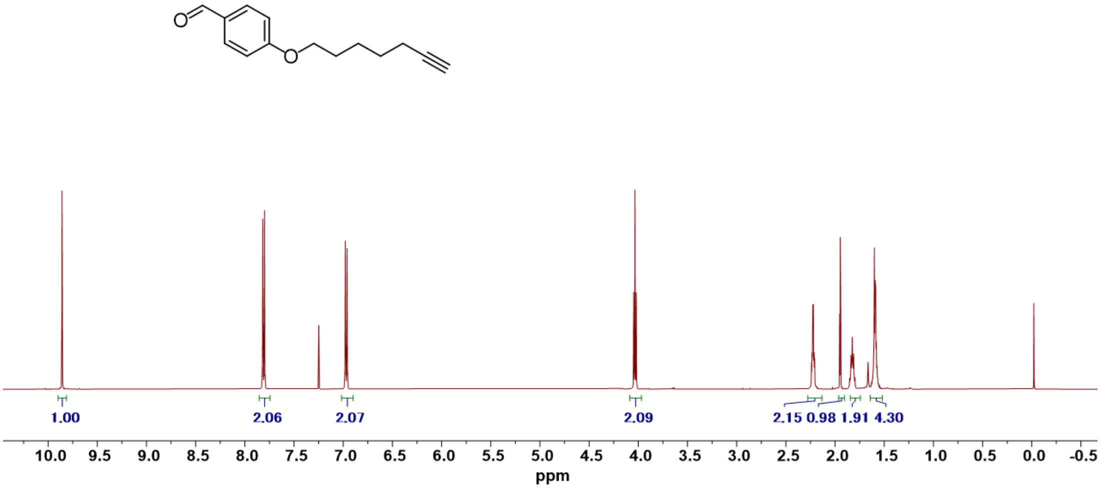

^{13}C NMR (126 MHz, $CHCl_3$-*d*, 298 K) spectrum of 7

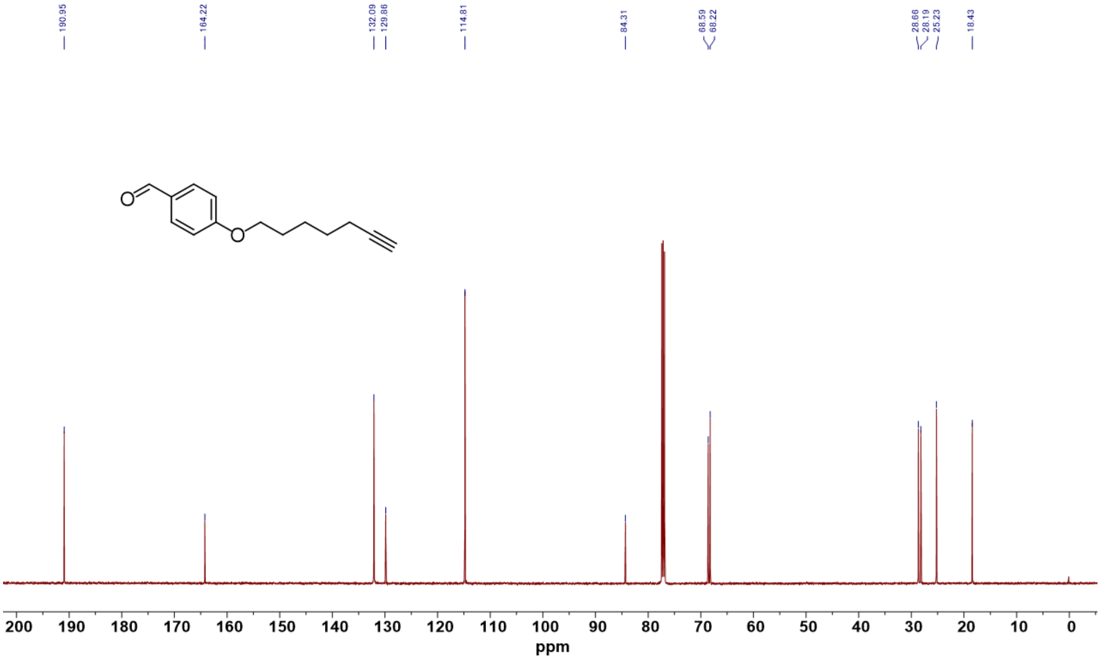

Synthesis of 8

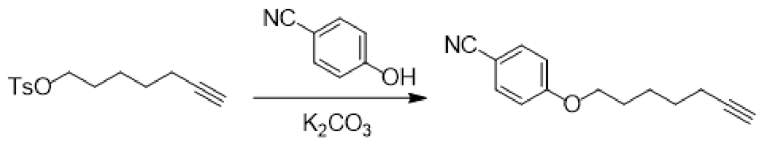

6 8

To a solution of 6 (3.15 g, 11.8 mmol) and 4-Hydroxybenzonitrile (1.34 g, 11.3 mmol) in DMF (30 mL) was added K_2CO_3 (4.67 g, 33.8 mmol). After bubbling by N_2 for 10 minutes, the mixture was stirred at 70 °C overnight. The reaction mixture was poured into water and extracted with ethyl acetate. The organic phase was washed with water (2x) and brine (1x), dried over $MgSO_4$ and evaporated. 8 was obtained as a white solid (Yield: 2.13 g, 89%). ^{1}H NMR (500 MHz, $CHCl_3$-*d*) δ 7.56 (d, *J* = 8.9 Hz, 2H), 6.91 (d, *J* = 8.9 Hz, 2H), 3.99 (t, *J* = 6.5 Hz, 2H), 2.22 (m, 2H), 2.26-2.17 (t, *J* = 2.6 Hz, 1H), 1.87 – 1.74 (m, 2H), 1.63-1.50 (m, 4H).^{13}C NMR (126 MHz, $CHCl_3$-*d*) δ 162.42, 134.07, 119.42, 115.23, 103.80, 84.28, 68.60, 68.21, 28.60, 28.16, 25.19, 18.42. HR-MS (ESI): calcd for , $[C_{14}H_{16}NO+H]^+$, m/z =214.1227, found m/z = 214.1223.

^{1}H NMR (500 MHz, $CHCl_3$-*d*, 298 K) spectrum of 8

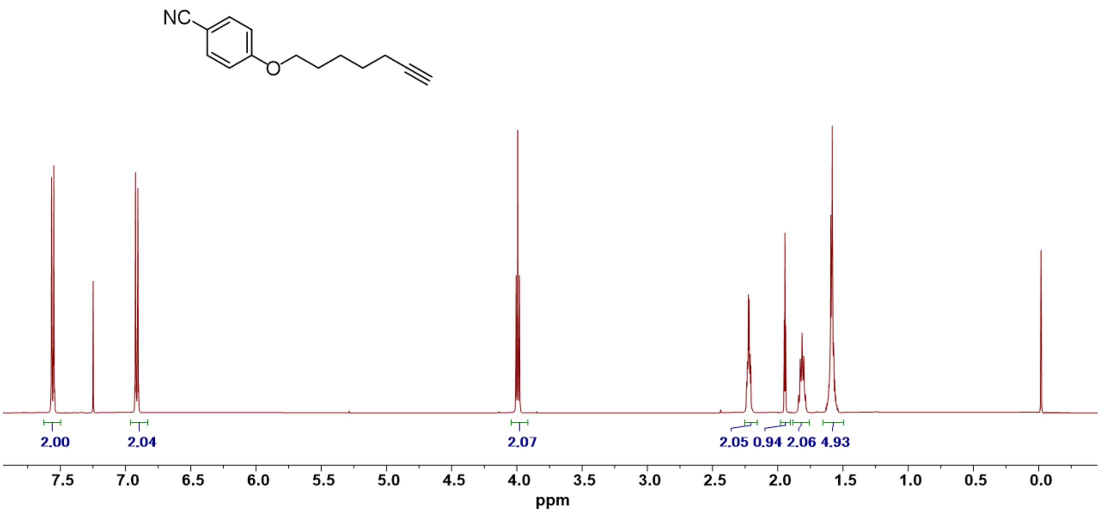

^{13}C NMR (126 MHz, $CHCl_3$-*d*, 298 K) spectrum of 8

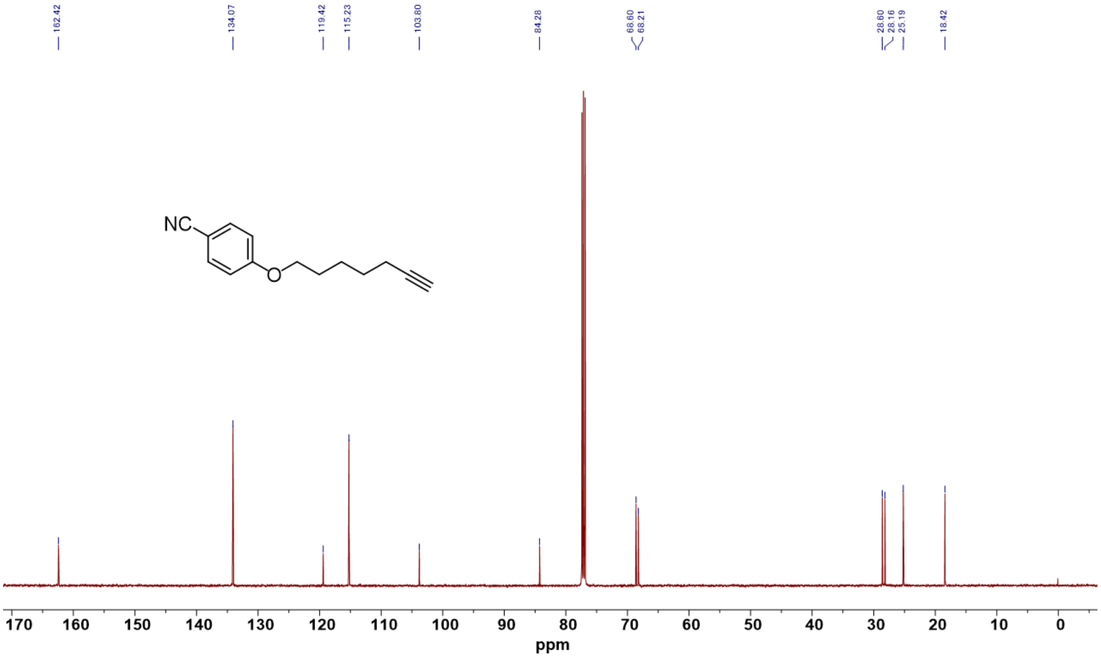

Synthesis of 9

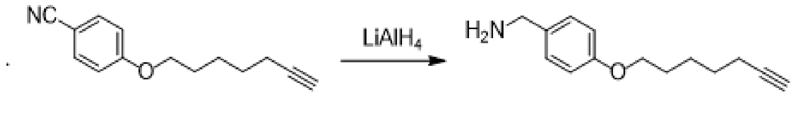

8 9

8 (2.00 g, 9.38 mmol) was dissolved in dry THF (40 mL). $LiAlH_4$ (1.42 g, 37.5 mmol) was added to the solution in portions under ice bath. The reaction mixture was stirred at room temperature for 4 h. The reaction was quenched by dropwise addition of water under ice cooling until gas evolution ceased. The resulting mixture was diluted with CH_2Cl_2 (60 mL) and filtered. The filtrate was dried over Na_2SO_4, filtered, and concentrated under reduced pressure to give 9 as a white solid (1.94 g, 95%).^{1}H NMR (500 MHz, $CHCl_3$-*d*) δ 7.20 (d, *J* = 8.6 Hz, 2H), 6.85 (d, *J* = 8.6 Hz, 2H), 3.94 (t, *J* = 6.5 Hz, 2H), 3.78 (s, 2H), 2.21 (m, 2H), 2.25-2.16 (t, *J* = 2.6 Hz, 1H), 1.85 – 1.73 (m, 2H), 1.61 – 1.56 (m, 4H).^{13}C NMR (126 MHz, $CHCl_3$-*d*) δ 158.06, 135.60, 128.31, 114.63, 84.46, 68.43, 67.87, 46.03, 28.89, 28.30, 25.35, 18.45. HR-MS (ESI): calcd for , $[C_{14}H_{20}NO+H]^+$, m/z = 218.1540, found m/z = 217.1538.

^{1}H NMR (500 MHz, CHCl3-*d*, 298 K) spectrum of 9

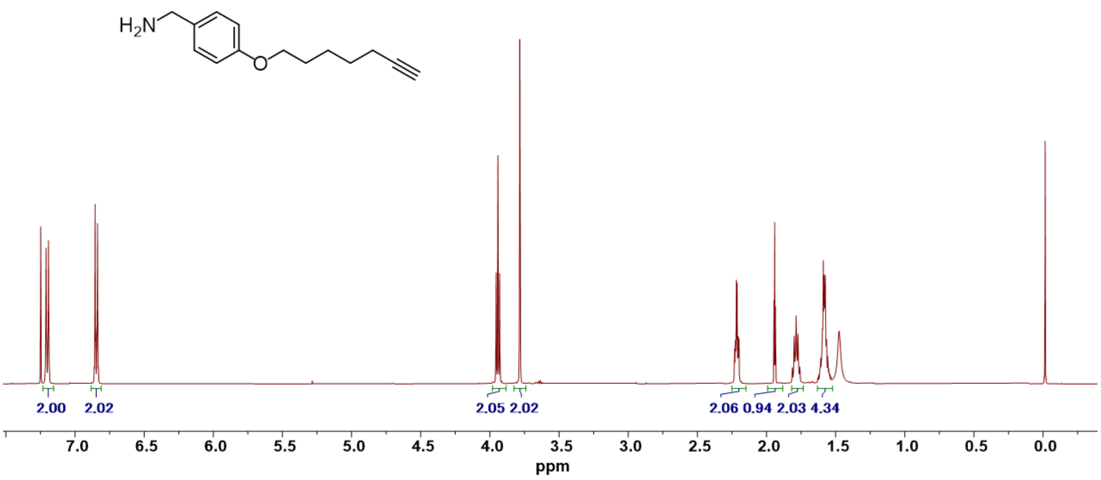

^{13}C NMR (126 MHz, CHCl3-*d*, 298 K) spectrum of 9

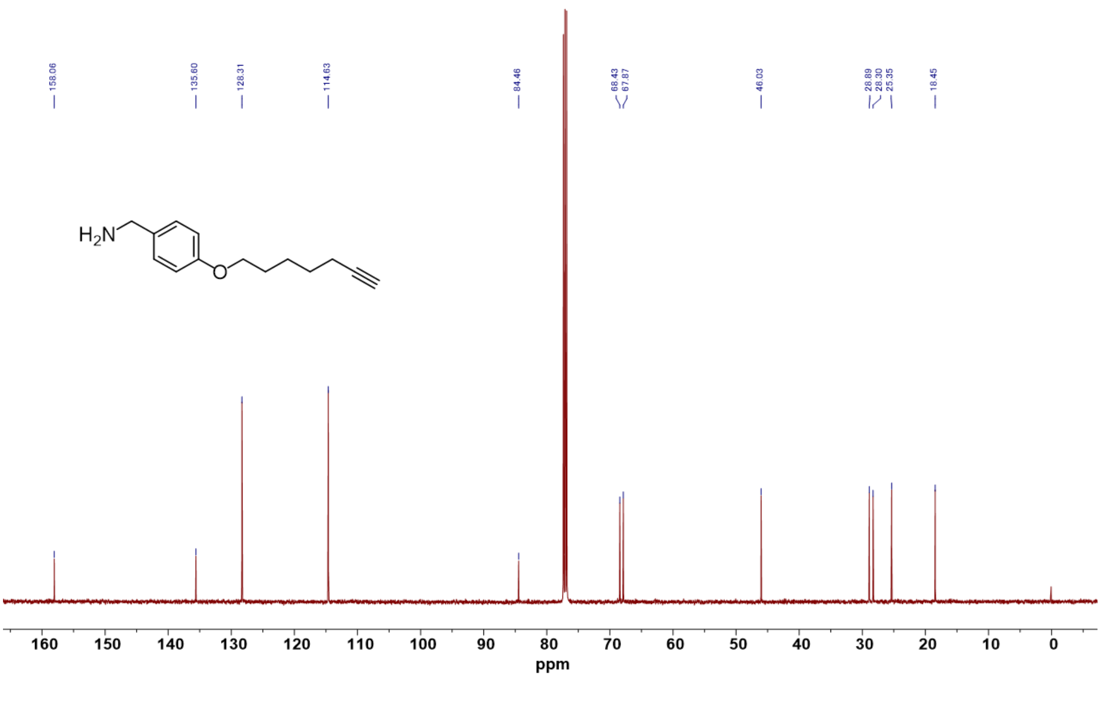

Synthesis of 10

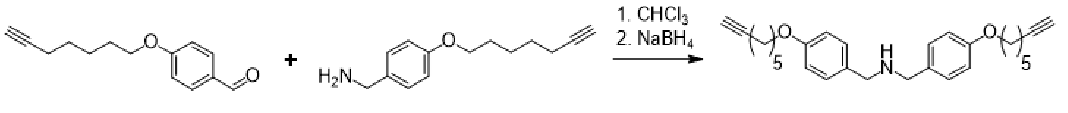

7 (2.00 g, 9.20 mmol) and 9 (1.99 g, 9.20 mmol) were dissolved in dry $CHCl_3$ (20 mL) and stirred at room temperature overnight. The solvent was removed under reduced pressure, and the residue was redissolved in a mixture solvent of THF and MeOH (30 mL, v/v = 2/1). $NaBH_4$ (1.39 g, 36.8 mmol) was added to the solution in portions, and the mixture was stirred at 65 °C for 1 h. The reaction was quenched by addition of water, followed by extraction with ethyl acetate and washing with water and the brine. The organic layer was separated, dried over Na_2SO_4, filtered, and concentrated under reduced pressure to give compound 10 as a yellow solid (Yield: 3.35 g, 87%). ^{1}H NMR (400 MHz, $CHCl_3$-*d*) δ 7.22 (d, *J* = 8.6 Hz, 4H), 6.84 (d, *J* = 8.6 Hz, 4H), 3.94 (t, *J* = 6.5 Hz, 4H), 3.71 (s, 4H), 2.29 – 2.16 (m, 4H), 1.94 (t, *J* = 2.6 Hz, 2H), 1.83 – 1.70 (m, 4H), 1.64-1.51 (m, 8H). ^{13}C NMR (101 MHz, $CHCl_3$-*d*) δ 158.14, 132.46, 129.40, 114.43, 84.49, 68.45, 67.81, 52.55, 28.91, 28.31, 25.36, 18.47. HR-MS (ESI): calcd for , $[C_{28}H_{36}NO_2+H]^+$, m/z = 418.2741, found m/z = 418.2740.

^1H NMR (400 MHz, $CHCl_3$-*d*, 298 K) spectrum of 10

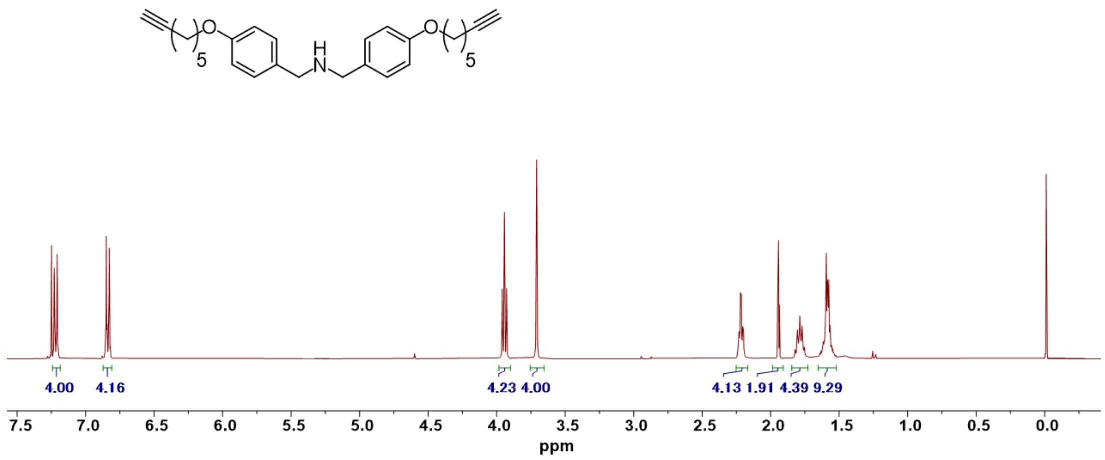

^{13}C NMR (101 MHz, $CHCl_3$-*d*, 298 K) spectrum of 10

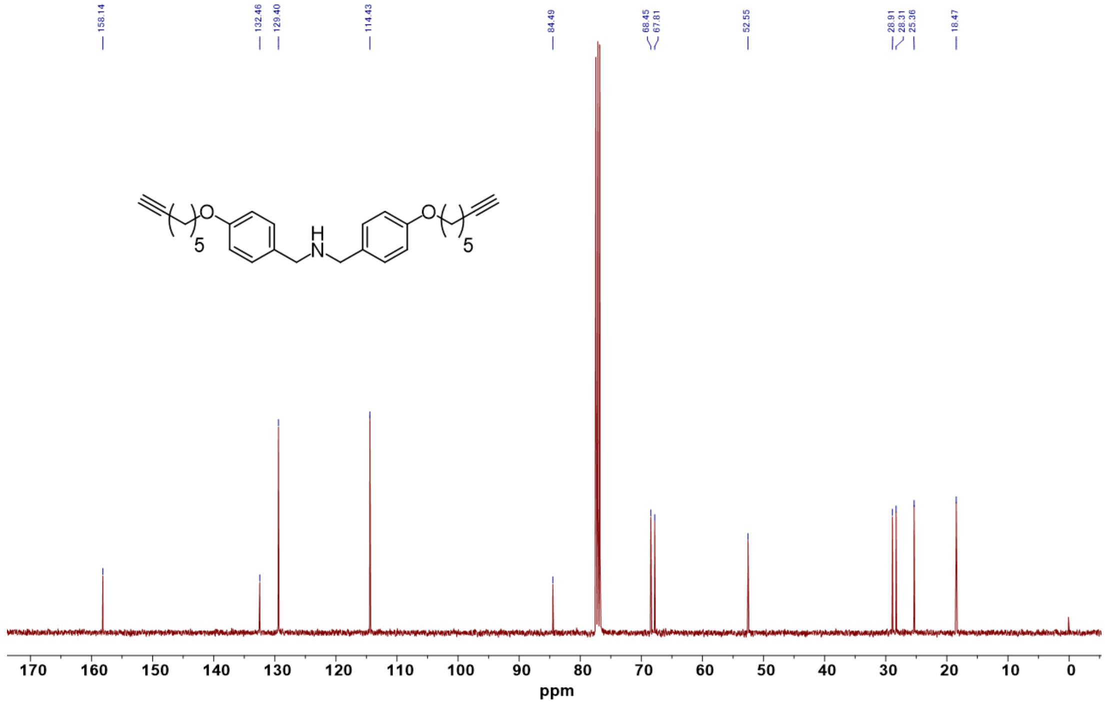

Synthesis of G

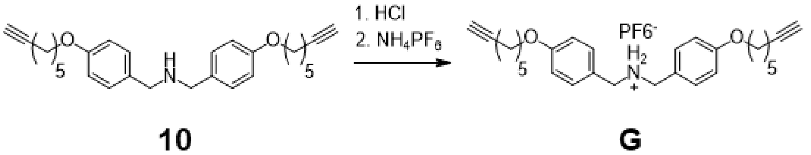

10 (2.00 g, 4.79 mmol) was dissolved in MeOH (20 mL) and CH_2Cl_2 (20 mL). 6 mL concentrated HCl was added to the solution and stirred at room temperature for 1 hour. The white precipitate was collected via suction filtration, washed by water. The resulting solid was dispersed in CH_2Cl_2 (30 mL), followed by addition of saturated aqueous solution of NH_4PF_6 (15 mL). The mixture was stirred at room temperature overnight. The reaction was extracted with CH_2Cl_2 and water, and the organic layer was separated and evaporated. G was obtained as a white solid (Yield: 2.30 g, 85%).^{1}H NMR (500 MHz, $CHCl_3$-*d*) δ 7.24 (d, *J* = 9.0 Hz, 4H), 6.87 (d, *J* = 8.7 Hz, 4H), 4.01 (s, 4H), 3.89 (t, *J* = 6.4 Hz, 4H), 2.25-2.16 (m, 4H), 1.94 (t, *J* = 2.6 Hz, 2H), 1.78 – 1.70 (m, 4H), 1.59 – 1.48 (m, 8H).^{13}C NMR (126 MHz, $CHCl_3$-*d*) δ 160.36, 131.36, 121.37, 115.42, 84.39, 68.55, 67.90, 50.61, 28.74, 28.24, 25.26, 18.43. HR-MS (ESI): calcd for , $[C_{28}H_{36}F_6NO_2P\text{-}PF_6]^+$, m/z = 418.2741, found m/z = 418.2739.

^{1}H NMR (500 MHz, $CHCl_3$-*d*, 298 K) spectrum of G

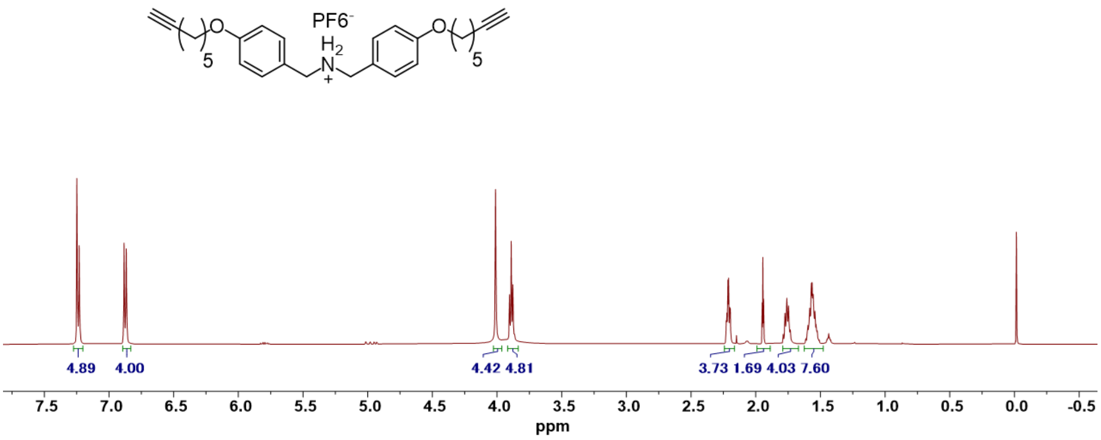

^{13}C NMR (126 MHz, $CHCl_3$-*d*, 298 K) spectrum of G

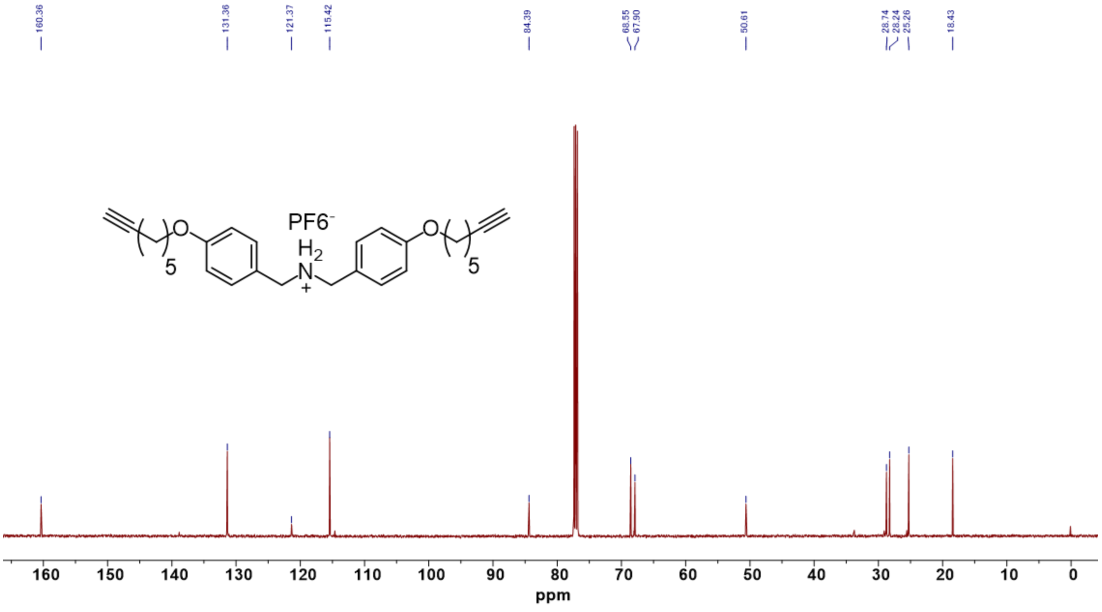

Synthesis of 11

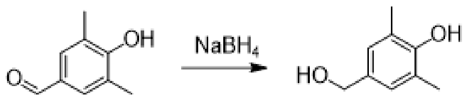

11

4-Hydroxy-3,5-dimethylbenzaldehyde (3.00 g, 20.0 mmol) MeOH (60 mL). $NaBH_4$ (3.02 g, 79.9 mmol) was added to the solution in portions, and the mixture was stirred at room temperature overnight. The reaction was quenched by addition of water, followed by extraction with ethyl acetate and washing with water and the brine. The organic layer was separated, dried over Na_2SO_4, filtered, and concentrated under reduced pressure to give compound 11 as a yellow solid (Yield: 2.89 g, 95%). ^{1}H NMR (500 MHz, DMSO-D_6) δ 8.00 (s, 1H), 6.79 (s, 2H), 4.84 (t, J = 5.7 Hz, 1H), 4.27 (d, J = 5.7 Hz, 2H), 2.10 (s, 6H).

Data was consistent with those reported in the literature [3].

^{1}H NMR (500 MHz, DMSO-D_6, 298 K) spectrum of 11

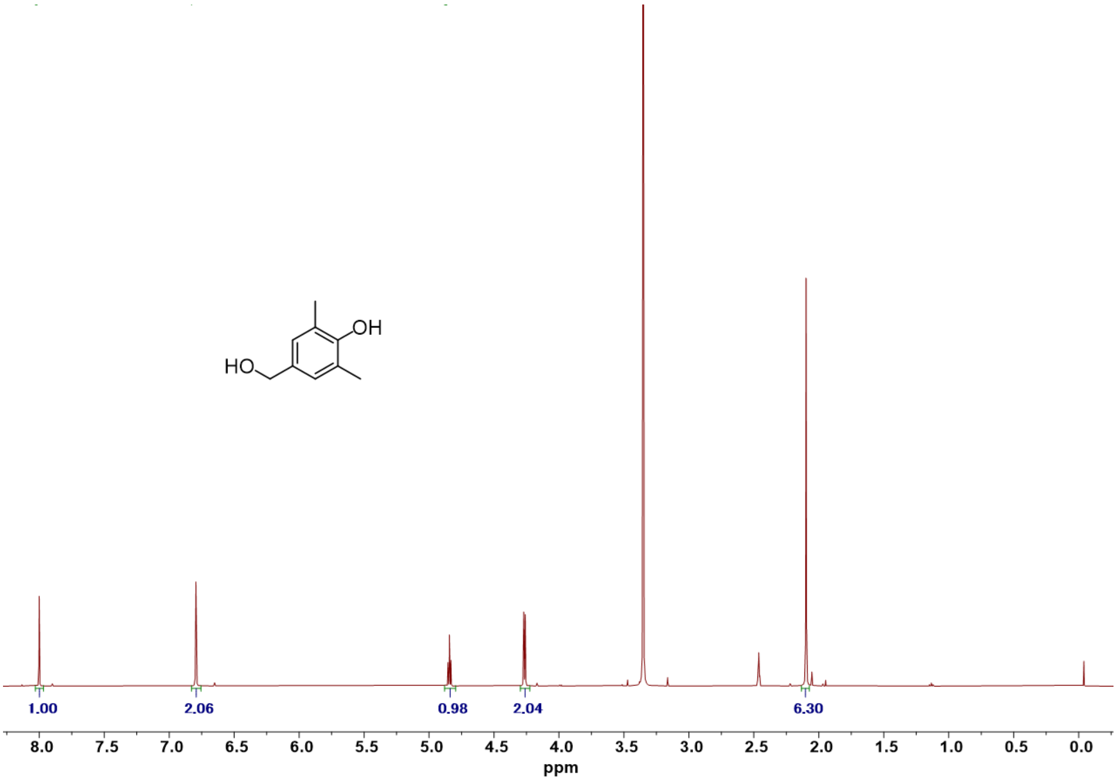

Synthesis of 12

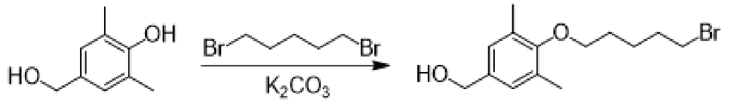

11 **12**

To a solution of 11 (4.20 g, 27.6 mmol) and 1,5-dibromopentane (25.4 g, 0.11 mol) in CH_3CN (100 mL) was added K_2CO_3 (7.63 g, 55.19 mmol) under N_2 atmosphere. After stirring at 75 °C overnight, the reaction mixture was poured into water and extracted with ethyl acetate. The organic phase was washed with water (3 x) and brine (1x), dried over $MgSO_4$ and evaporated. The crude was purified via silica column chromatography (Hexane/Ethyl acetate). 12 was obtained as yellow oil (Yield: 7.21 g, 86%). ^{1}H NMR (500 MHz, $CHCl_3$-*d*) δ 6.99 (s, 2H), 4.55 (s, 2H), 3.74 (t, J = 6.4 Hz, 2H), 3.45 (t, J = 6.8 Hz, 2H), 2.26 (s, 6H), 1.99-1.90 (m, 2H), 1.84 – 1.77 (m, 2H), 1.71 – 1.62 (m, 2H).^{13}C NMR (126 MHz, $CHCl_3$-*d*) δ 155.50, 136.14, 131.15, 127.80, 71.94, 65.20, 33.78, 32.78, 29.68, 25.00, 16.41. HR-MS (ESI): calcd for , $[C_{14}H_{22}BrO_2+H]^+$, m/z = 301.0798, found m/z = 301.0799.

^{1}H NMR (500 MHz, $CHCl_3$-*d*, 298 K) spectrum of 12

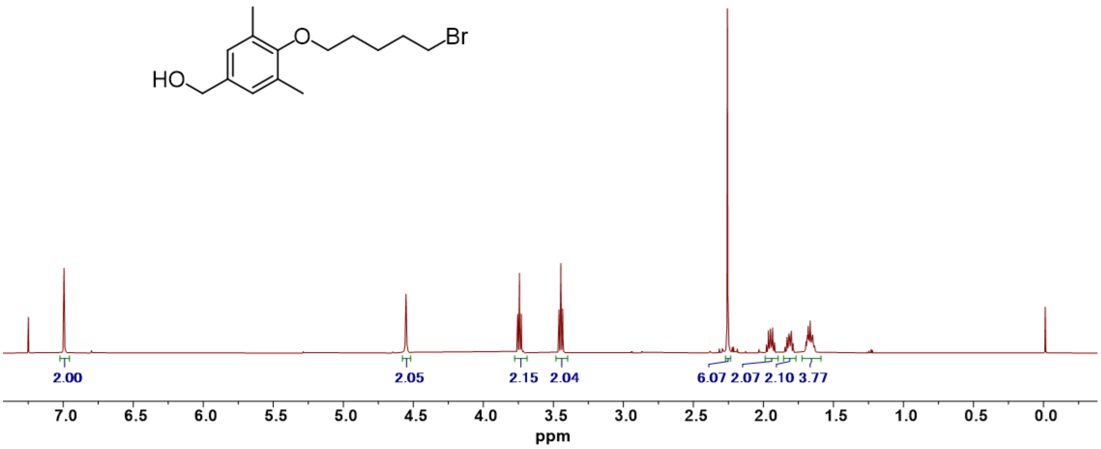

^{13}C NMR (126 MHz, $CHCl_3$-*d*, 298 K) spectrum of 12

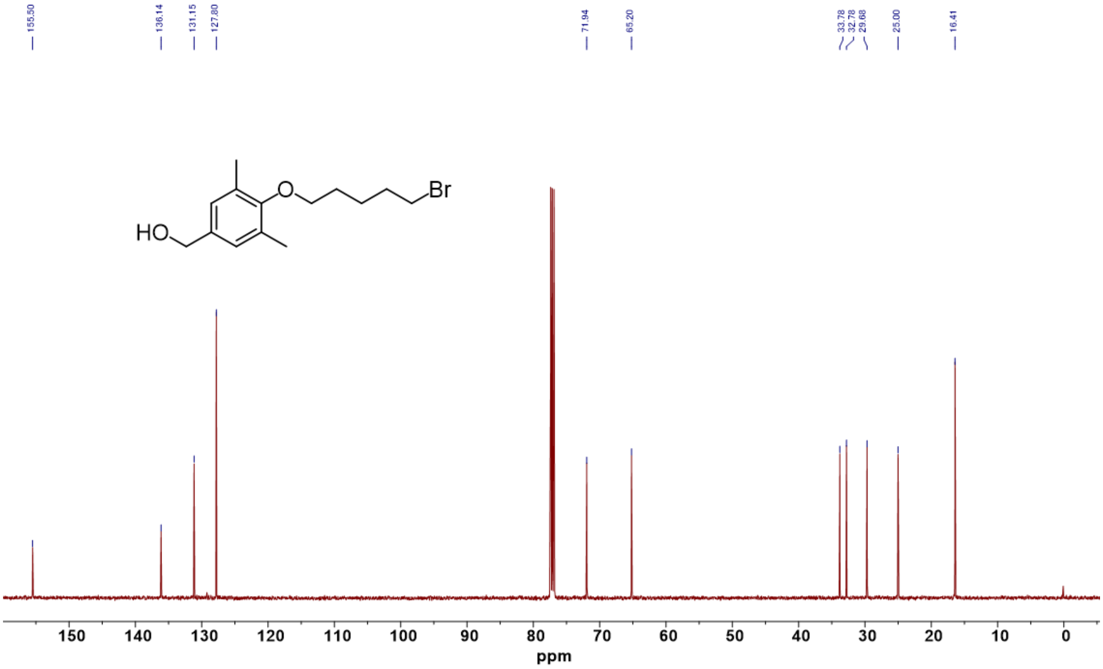

Synthesis of 13

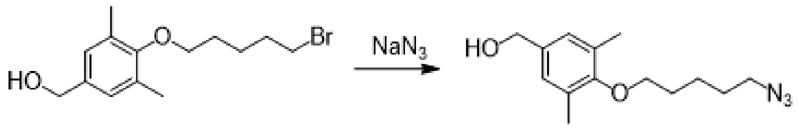

12 **13**

To a solution of 12 (3.00 g, 9.96 mmol) and NaN_3 (1.29 g, 19.9 mmol) was dissolved in DMF (40 mL). After stirring at 65 °C for 3 h, the reaction mixture was poured into water and extracted with ethyl acetate. The organic phase was washed with water (3 x) and brine (1 x), dried over $MgSO_4$ and evaporated. 13 was obtained as a yellow oil (Yield: 2.31 g, 88%). ^{1}H NMR (500 MHz, $CHCl_3$-*d*) δ 7.00 (s, 2H), 4.56 (d, *J* = 4.6 Hz, 2H), 3.74 (t, *J* = 6.4 Hz, 2H), 3.31 (t, *J* = 6.8 Hz, 2H), 2.26 (s, 6H), 1.87 – 1.78 (m, 2H), 1.71 – 1.65 (m, 2H), 1.64 – 1.56 (m, 2H). ^{13}C NMR (126 MHz, $CHCl_3$-*d*) δ 155.50, 136.14, 131.16, 127.80, 71.93, 65.21, 51.48, 30.06, 28.93, 23.57, 16.39. HR-MS (ESI): calcd for , $[C_{14}H_{22}N_3O_2+H]^+$, m/z = 264.1707, found m/z = 264.1705.

^{1}H NMR (500 MHz, $CHCl_3$-*d*, 298 K) spectrum of 13

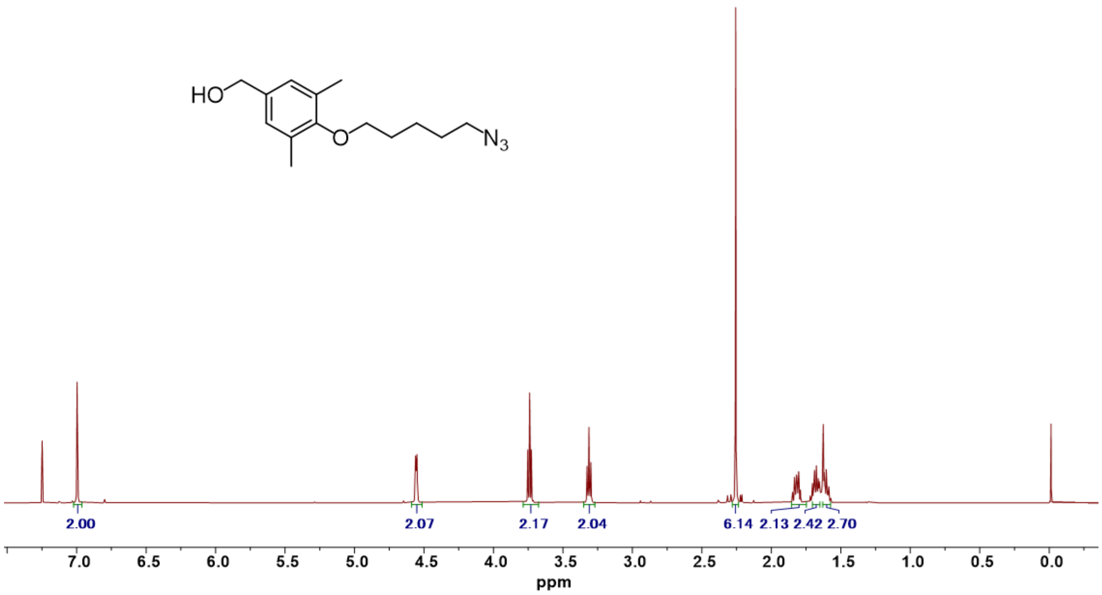

^{13}C NMR (126 MHz, $CHCl_3$-*d*, 298 K) spectrum of 13

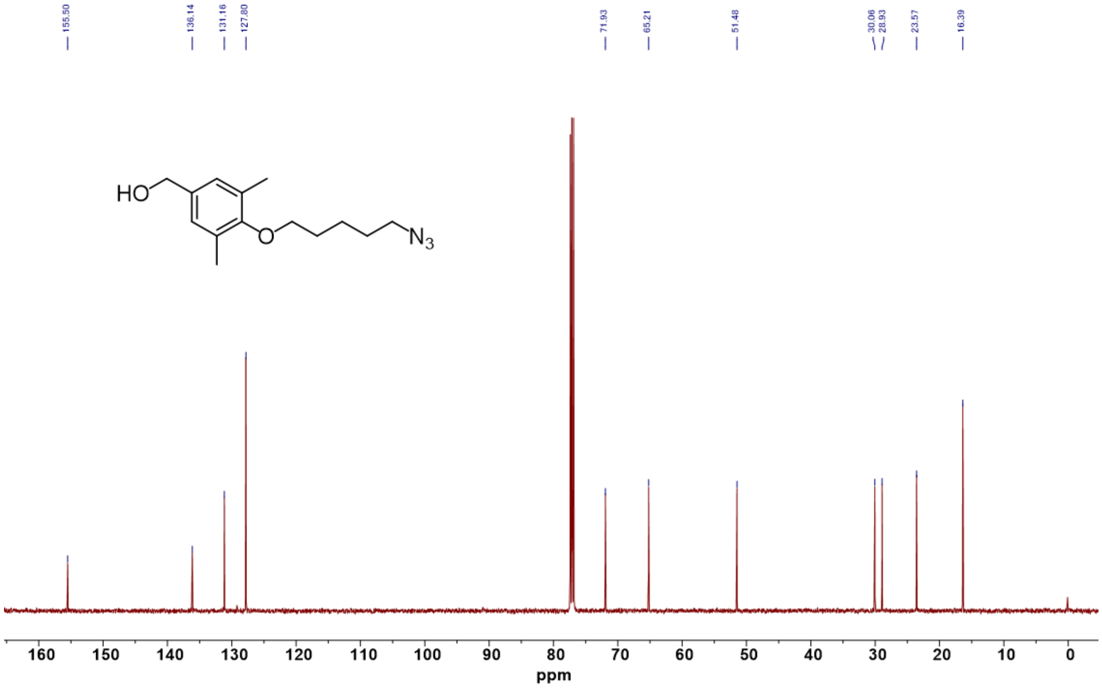

Synthesis of RM

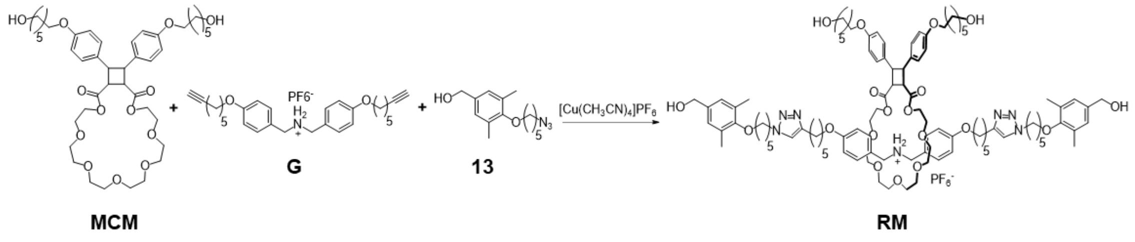

MCM (0.25 g, 0.32 mmol), G (0.27 g, 0.48 mmol), and 13 (0.30 g, 1.13 mmol) were dissolved in dry and degassed CH_2Cl_2 (7 mL). After the solution was stirred for 1 h, $[Cu(CH_3CN)_4]PF_6$ (0.12 g, 0.32 mmol) were added under N_2 atmosphere. Stir the mixture overnight, then diluted with CH_2Cl_2 (50 mL) and wash with EDTA·2Na solution (aq, 0.1 M, 2 x) and H_2O (2 x), dried over $MgSO_4$ and evaporated. The crude was purified via the silica column chromatography (CH_2Cl_2/ Methanol = 100/0 – 95/5, gradient elution). RM was isolated as white solid. (Yield: 0.26 g, 45%). For details, see Supplementary Figs. 8-12. ^{1}H NMR (500 MHz, $CHCl_3$-*d*) δ 7.75-7.67 (br s, 2H), 7.41 (s, 2H), 7.26 (d, *J* = 8.7 Hz, 4H), 7.00 (d, *J* = 8.7 Hz, 4H), 6.95 (s, 4H), 6.91 (d, *J* = 8.7 Hz, 4H), 6.71 (d, *J* = 8.7 Hz, 4H), 4.52 (s, 4H), 4.44-4.37(m, 2H), 4.33 (t, *J* = 7.2 Hz, 4H), 4.10-4.27 (m, 6H), 3.96 (t, *J* = 6.6 Hz, 4H), 3.86 – 3.78 (m, 8H), 3.69 (t, *J* = 6.3 Hz, 8H), 3.61 (t, *J* = 6.6 Hz, 4H), 3.56-3.45 (m, 6H), 3.38 – 3.33 (m, 2H), 3.29 – 3.19 (m, 4H), 3.00 (t, *J* = 4.2 Hz, 4H), 2.71 (t, *J* = 7.6 Hz, 4H), 2.20 (s, 12H), 1.99-1.89 (m, 8H), 1.83-1.66 (m, 16H), 1.60 – 1.48 (m, 8H), 1.45 – 1.33 (m, 8H). ^{13}C NMR (126 MHz, $CHCl_3$-*d*) δ 172.26, 160.23, 158.40, 155.35, 136.31, 132.21, 131.47, 130.94, 127.76, 127.74, 122.50, 115.00, 114.71, 71.82, 71.22, 71.14, 70.97, 70.53, 70.01, 68.05, 67.93, 65.12, 64.94, 62.74, 51.72, 50.22, 48.08,

44.43, 32.74, 30.29, 29.84, 29.30, 29.20, 28.92, 25.95, 25.65, 23.33, 16.38. HR-MS (ESI): calcd for , $[C_{98}H_{140}F_6N_7O_{19}P-PF_6]^+$, m/z = 1720.0231, found m/z = 1720.0234.

^{1}H NMR (500 MHz, $CHCl_3$-*d*, 298 K) spectrum of RM

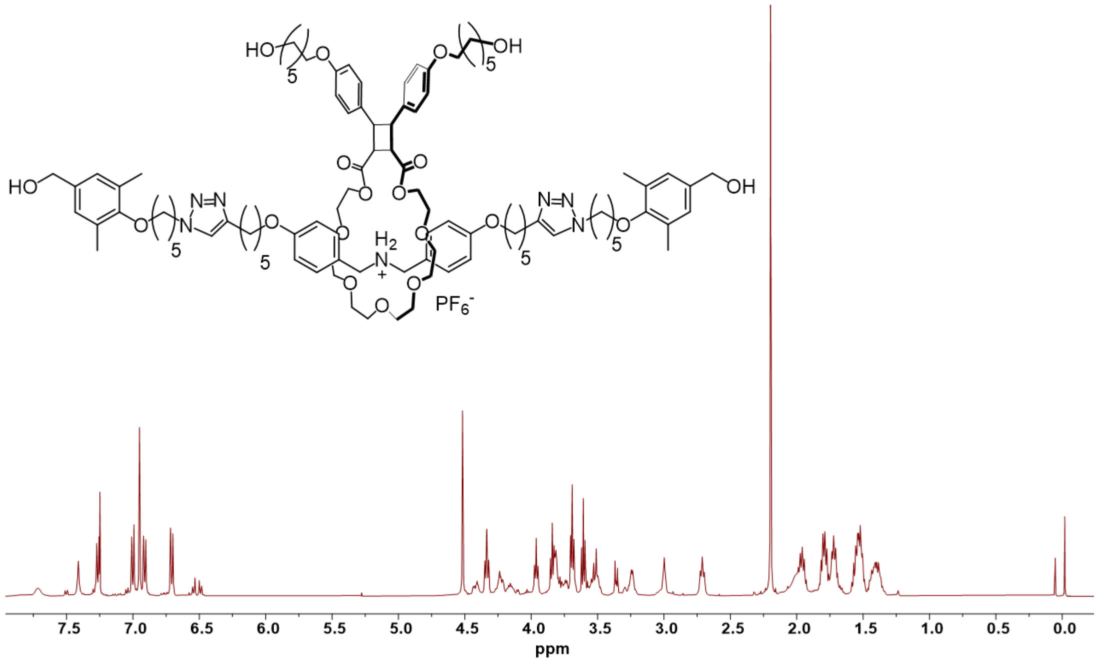

^{13}C NMR (126 MHz, $CHCl_3$-*d*, 298 K) spectrum of RM

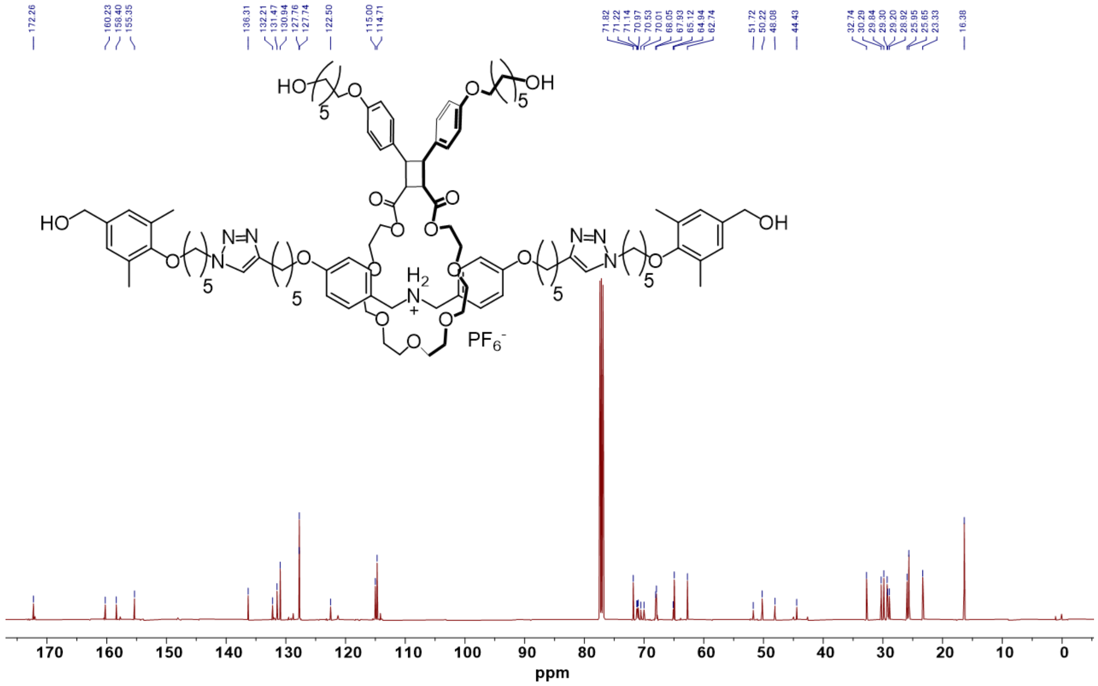

Electrospray ionization mass spectrum of RM

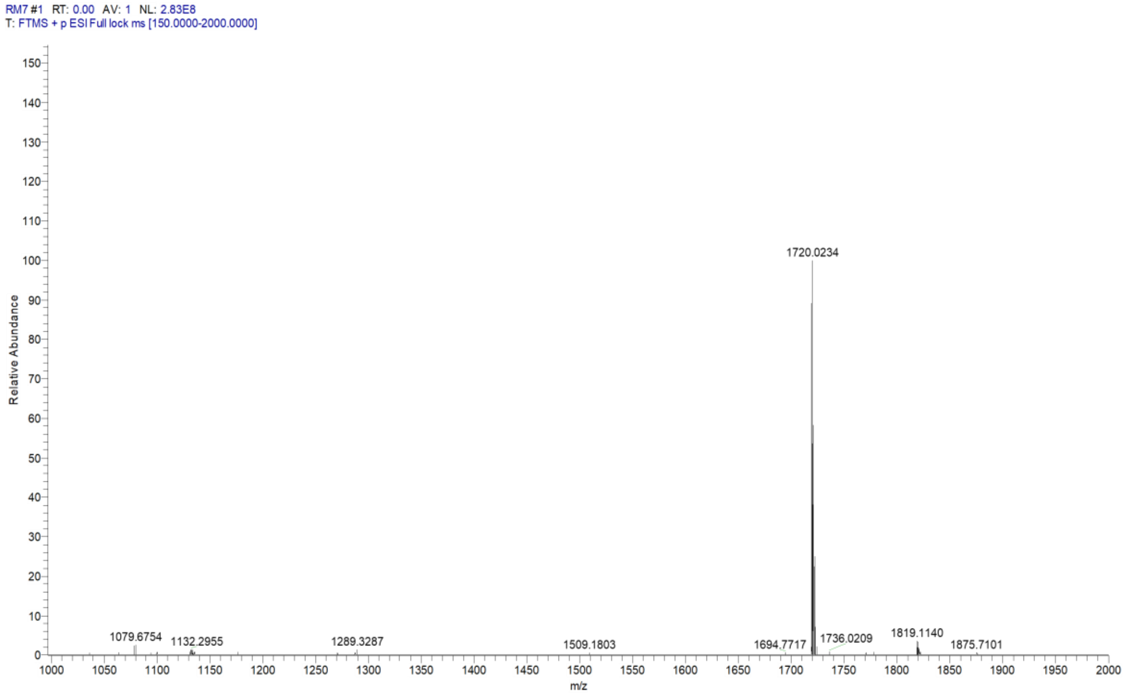

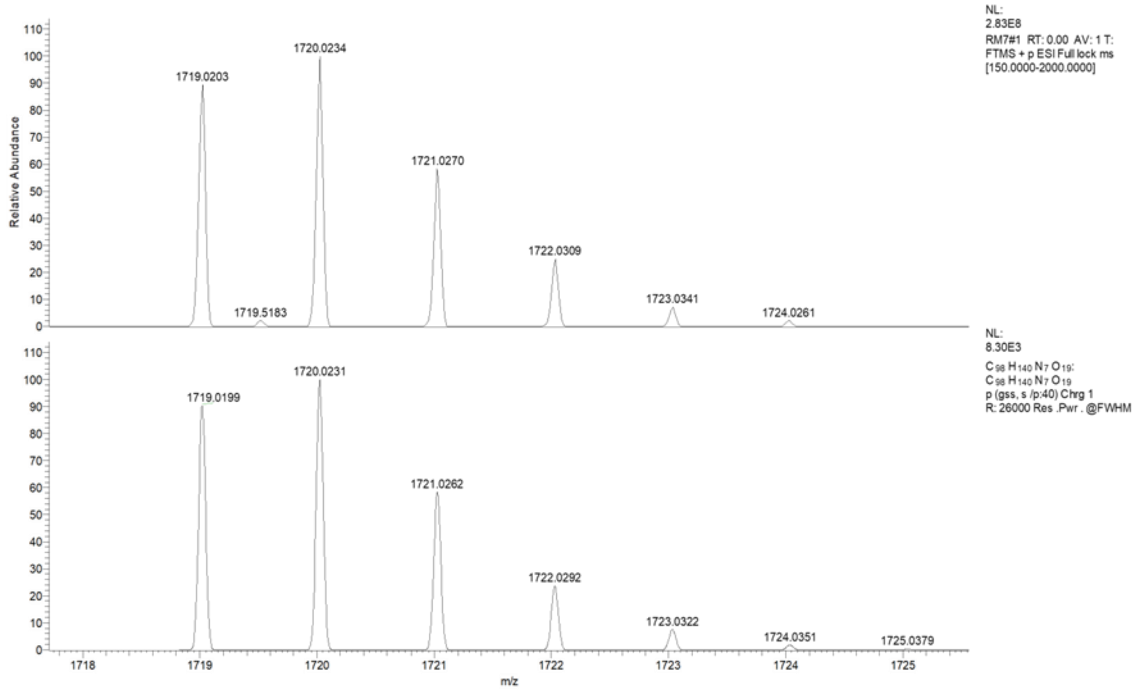

Synthesis of 14

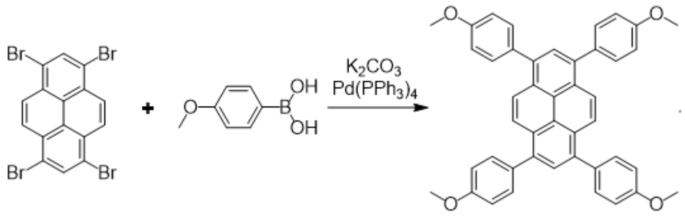

14

The solution of 1,3,6,8-Tetrabromopyrene (1.00 g, 1.93 mmol) and 4-methoxyphenylboronic acid (2.39 g, 15.7 mmol) in toluene (80 mL) was mixed with the solution of K_2CO_3 (4.30 g, 31.1 mmol) in water (20 mL). $Pd(PPh_3)_4$ (0.69 g, 0.60 mmol) was added under N_2 atmosphere and the mixture was heated to 95 °C overnight. The phases were separated and the aqueous layer was extracted with DCM (3 × 100 mL), dried over $MgSO_4$ and evaporated. 14 was isolated as a yellow powder (1.05 g, 87%). ^1H NMR (400 MHz, $CHCl_3$-*d*) δ 8.15 (s, 4H), 7.95 (s, 2H), 7.58 (d, *J* = 8.6 Hz, 8H), 7.07 (d, *J* = 8.7 Hz, 8H), 3.91 (s, 12H).

Data was consistent with those reported in the literature [4].

^{1}H NMR (400 MHz, $CHCl_3$-*d*, 298 K) spectrum of 14

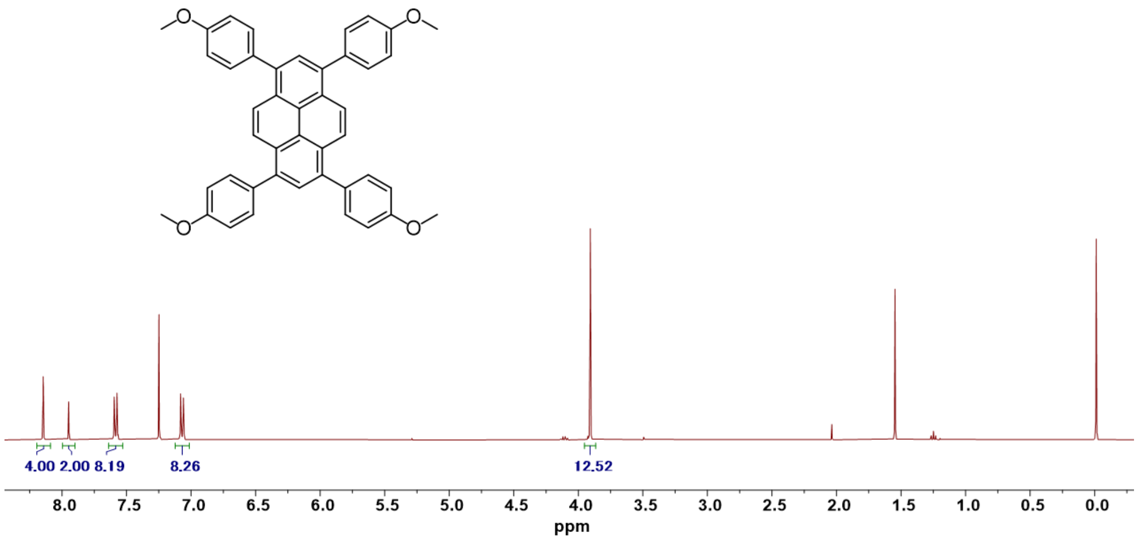

Synthesis of 15

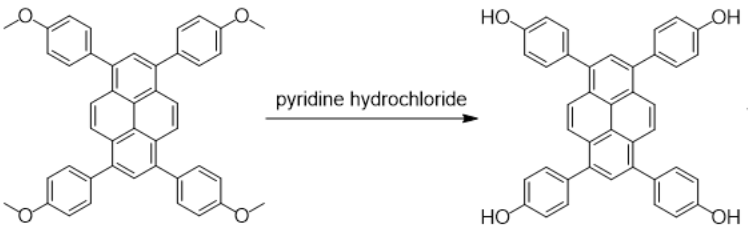

14 15

14 (0.50 g, 0.80 mmol) and pyridine hydrochloride (5.12 g, 44.7 mmol) were stirred at 220 °C for 3 h under N_2 atmosphere. The hot reaction mixture was then diluted with 10 mL warm water and slowly poured into 60 mL hot water. After cooling, the precipitated solid was filtered by suction and dried to afford 15 as a yellow solid (0.40 g, 89%). 1H NMR (500 MHz, DMSO-D_6) δ 9.74-9.54 (br s, 4H), 8.09 (s, 4H), 7.81 (s, 2H), 7.45 (d, J = 8.5 Hz, 8H), 6.94 (d, J = 8.6 Hz, 8H).

Data was consistent with those reported in the literature [4].

^{1}H NMR (500 MHz, DMSO-d_6, 298 K) spectrum of 15

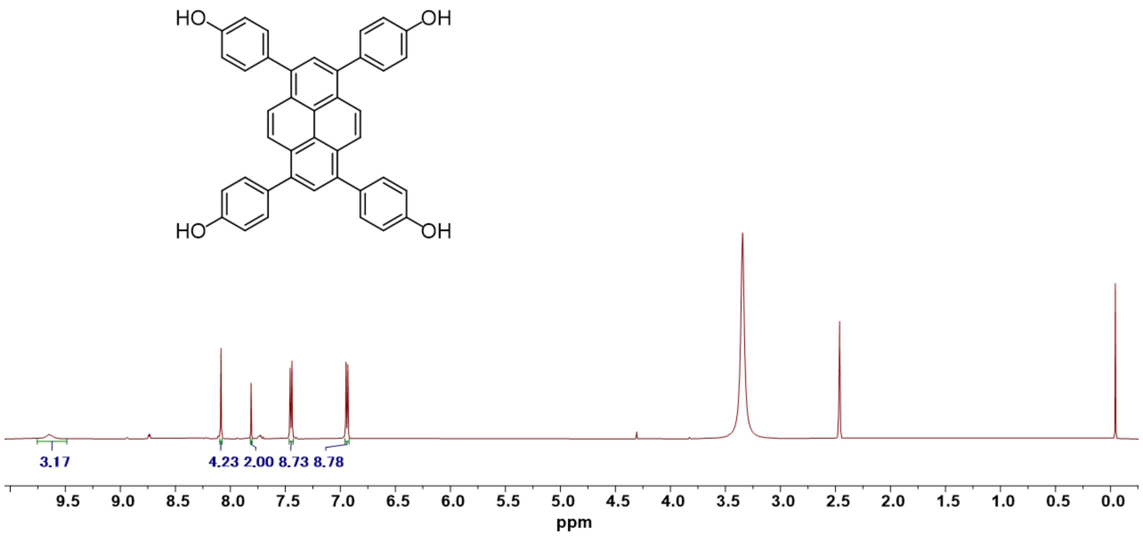

Synthesis of C

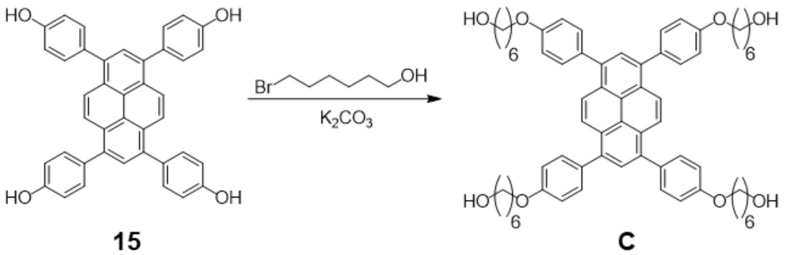

To a solution of 15 (0.40 g, 0.70 mmol) and 6-bromohexan-1-ol (0.76 g, 4.21 mmol) in DMF (20 mL) was added K_2CO_3 (0.76 g, 5.61 mmol)under N_2 atmosphere. After stirring at 70 °C overnight, the reaction mixture was poured into water and extracted with CH_2Cl_2. The organic phase was washed with water (3 x) and brine (1x), dried over $MgSO_4$ and evaporated. C were obtained as yellow solid (Yield: 0.32 g, 47%). ^1H NMR (400 MHz, $CHCl_3$-*d*) δ 8.14 (s, 4H), 7.93 (s, 2H), 7.55 (d, *J* = 8.7 Hz, 4H), 7.03 (d, *J* = 8.8 Hz, 4H), 4.04 (t, *J* = 6.4 Hz, 8H), 3.67 (t, *J* = 6.6 Hz, 8H), 1.93 – 1.79 (m, 8H), 1.69 – 1.34 (m, 24H). ^{13}C NMR (101 MHz, $CHCl_3$-*d*) δ 158.55, 136.84, 133.46, 131.77, 129.69, 128.07, 126.23, 125.20, 114.46, 68.05, 63.00, 32.79, 29.39, 26.04, 25.67. HR-MS (ESI): calcd for , $[C_{64}H_{75}O_8+H]^+$, m/z = 971.5457, found m/z = 971.5459.

^{1}H NMR (400 MHz, $CHCl_3$-*d*, 298 K) spectrum of C

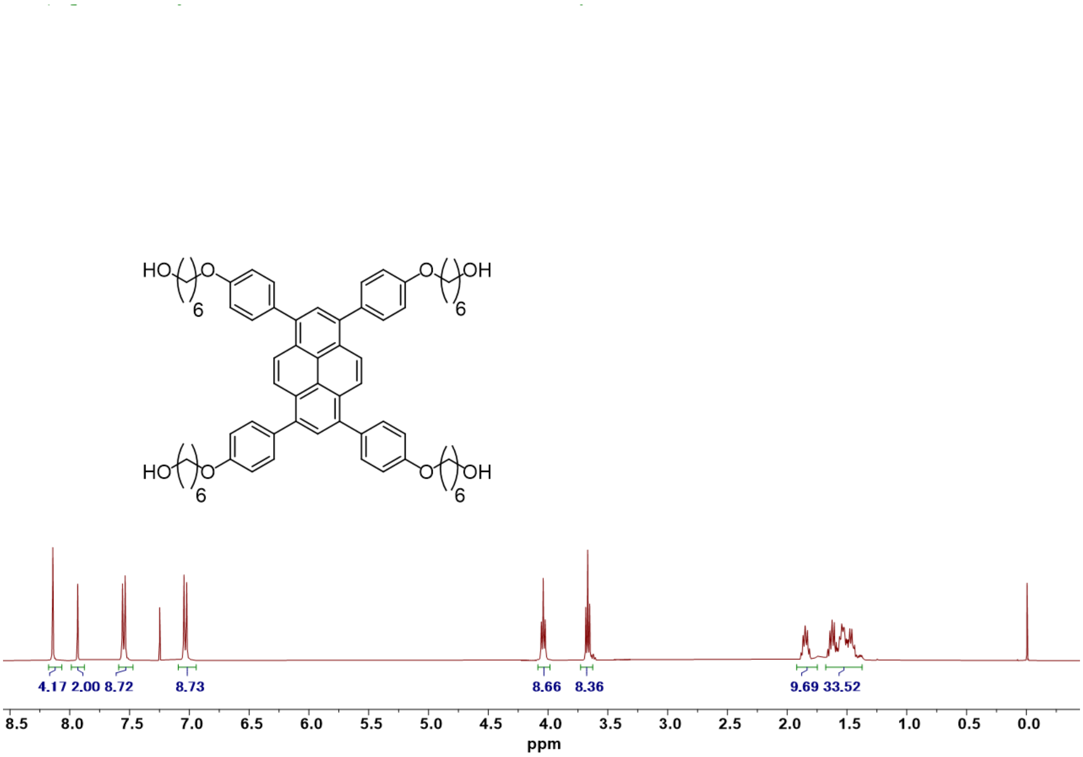

^{13}C NMR (101 MHz, $CHCl_3$-*d*, 298 K) spectrum of C

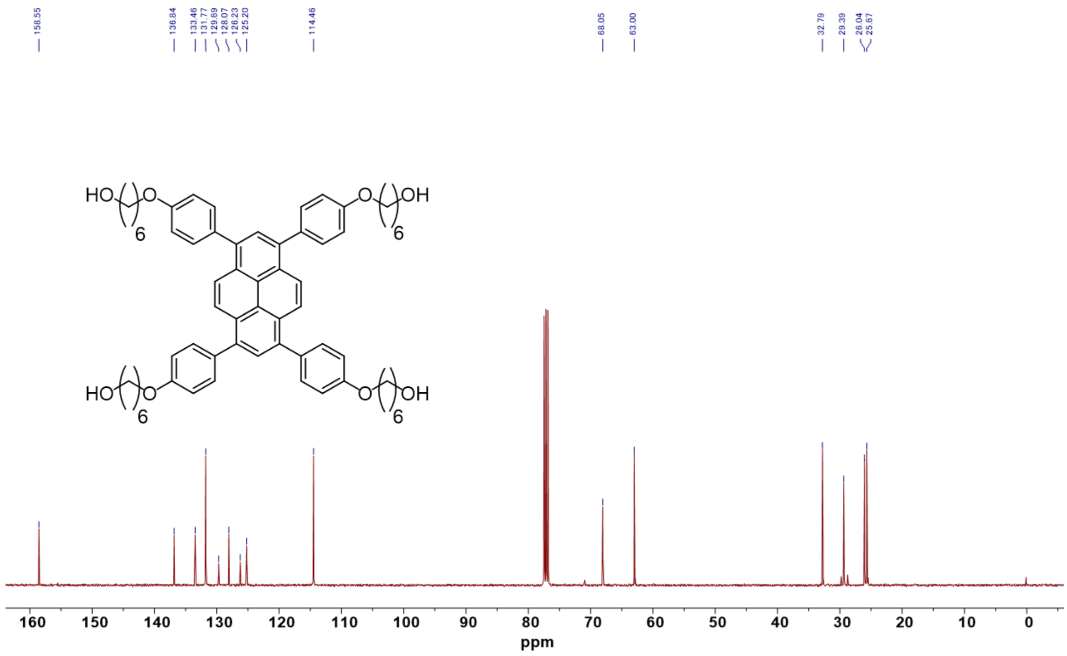

Synthesis of 16

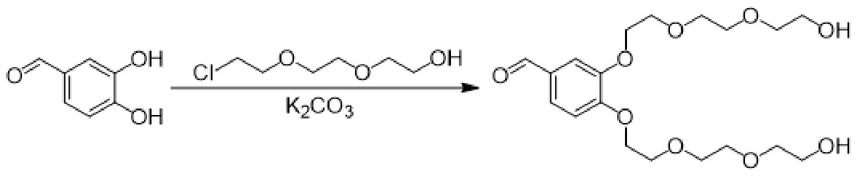

16

To a solution of 3,4-Dihydroxybenzaldehyde (3.00 g, 21.7 mmol) and 2-[2-(2-Chloroethoxy)ethoxy]ethanol (7.51 g, 44.5 mol) in DMF (50 mL) was added K_2CO_3 (12.0 g, 86.9 mmol) under N_2 atmosphere. After stirring at 100 °C overnight, the reaction mixture was poured into water and extracted with CH_2Cl_2. The organic phase was washed with water (3 x) and brine (1x), dried over $MgSO_4$ and evaporated. 16 was obtained as white solid (Yield: 7.86 g, 90%).^{1}H NMR (400 MHz, $CHCl_3$-*d*) δ 9.82 (s, 1H), 7.49 – 7.34 (m, 2H), 6.97 (d, *J* = 8.1 Hz, 1H), 4.38 – 4.07 (m, 4H), 3.91 (m, 4H), 3.80 – 3.50 (m, 20H).

Data was consistent with those reported in the literature [5].

^{1}H NMR (400 MHz, $CHCl_3$-*d*, 298 K) spectrum of 16

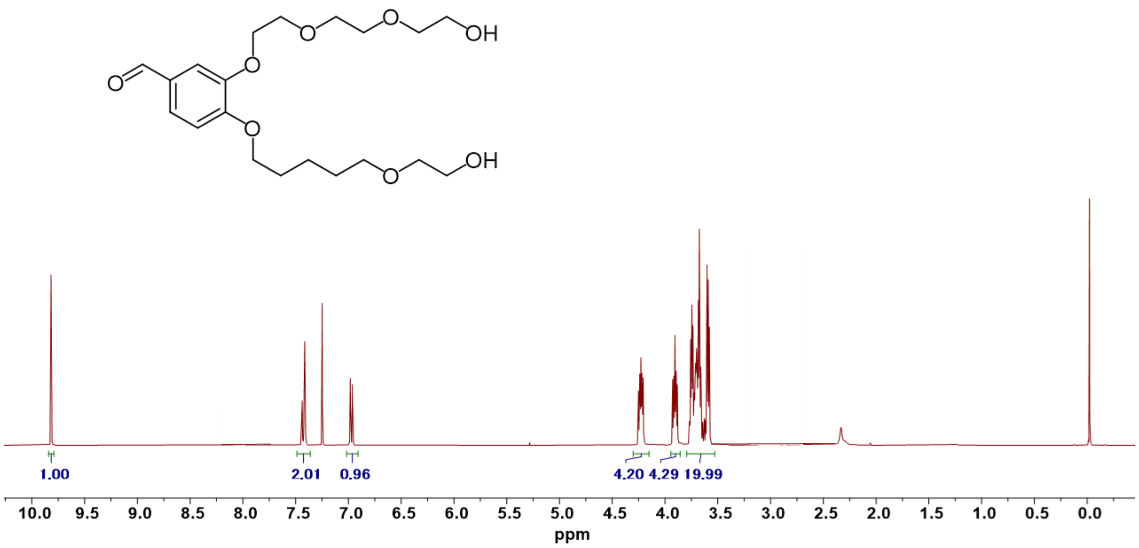

Synthesis of 17

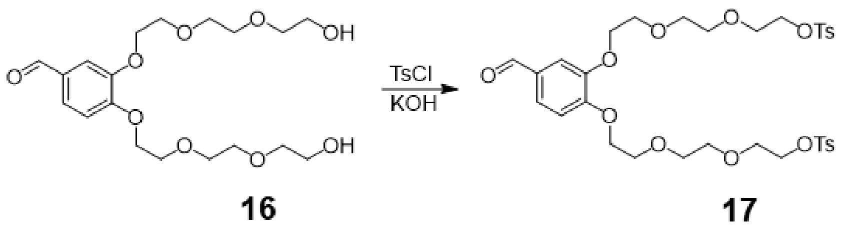

16 (3.80 g, 9.44 mmol) and p-toluenesulfonyl chloride (3.78 g, 19.8 mmol) were dissolved in CH_2Cl_2 (40 mL). KOH (4.24 g, 75.5 mmol) was added portionwise to the solution under an ice bath, and the reaction was stirred for 3 h. Then the reaction mixture was poured into water and extracted with CH_2Cl_2 (2x). The organic phase was washed with water (2x) and brine (1x), dried over Na_2SO_4 and evaporated. The crude was purified via silica column chromatography (CH_2Cl_2 /Ethyl acetate). 17 was obtained as yellow oil (Yield: 5.70 g, 85%). ^{1}H NMR (400 MHz, $CHCl_3$-*d*) δ 9.81 (s, 1H), 7.76 (d, J = 8.4 Hz, 4H), 7.48 – 7.36 (m, 2H), 7.33 – 7.28 (d, J = 8.4 Hz, 4H), 6.98 (d, J = 8.2 Hz, 1H), 4.41 – 4.03 (m, 8H), 3.95 – 3.77 (m, 4H), 3.71 – 3.45 (m, 12H), 2.41 (s, 6H).

Data was consistent with those reported in the literature [5].

^{1}H NMR (400 MHz, $CHCl_3$-*d*, 298 K) spectrum of 17

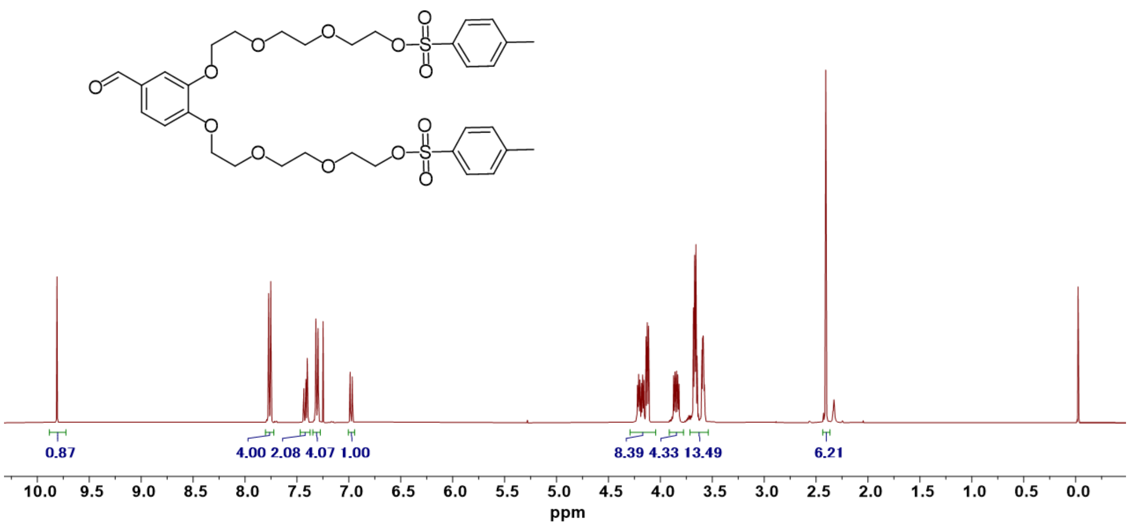

Synthesis of 18

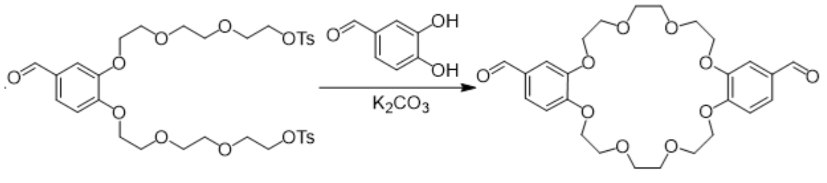

17 **18**

To a solution of 17 (2.00 g, 2.81 mmol) and 3,4-Dihydroxybenzaldehyde (0.39 g, 2.81 mmol) in CH_3CN (130 mL) was added K_2CO_3 (1.94 g, 14.1 mmol) under N_2 atmosphere. After stirring at 75 °C overnight, the reaction mixture was poured into water and extracted with CH_2Cl_2. The organic phase was washed with water (3 x) and brine (1x), dried over $MgSO_4$ and evaporated. The crude was purified via silica column chromatography (CH_2Cl_2 /Ethyl acetate). 18 was obtained as white solid (Yield: 0.66g, 47%). ^{1}H NMR (400 MHz, $CHCl_3$-*d*) δ 9.80 (s, 2H), 7.48 – 7.31 (m, 4H), 6.92 (d, J = 8.2 Hz, 2H), 4.27 – 4.15 (m, 8H), 4.01 – 3.73 (m, 16H).

Data was consistent with those reported in the literature [6].

^{1}H NMR (400 MHz, $CHCl_3$-*d*, 298 K) spectrum of 18

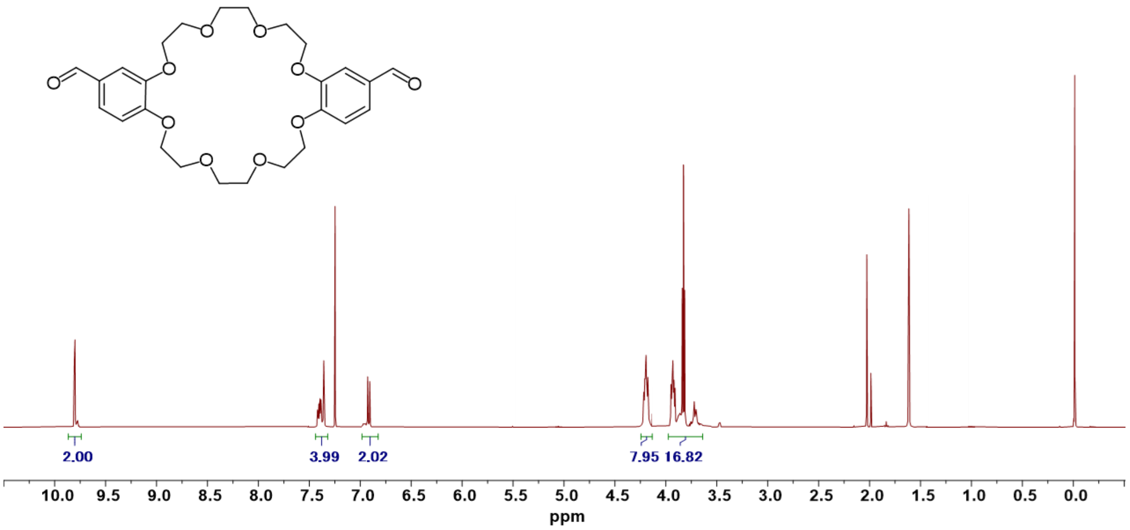

Synthesis of 19

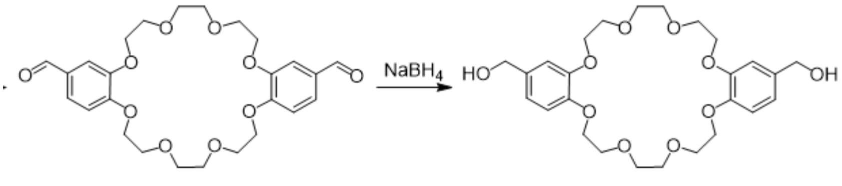

18 19

18 (1.70 g, 3.37 mmol) was dissolved in a mixture solvent of THF and MeOH (60 mL, v/v = 2/1). $NaBH_4$ (3.02 g, 79.9 mmol) was added to the solution in portions, and the mixture was stirred at room temperature overnight. The reaction was quenched by addition of water, followed by extraction with CH_2Cl_2 and washing with water and the brine. The organic layer was separated, dried over Na_2SO_4, filtered, and concentrated under reduced pressure to give compound 19 as a white solid (Yield: 1.45 g, 85%).^{1}H NMR (400 MHz, $CHCl_3$-*d*) δ 6.91 – 6.76 (m, 6H), 4.57 (br s, 4H), 4.17-4.06 (m, 8H), 3.93 – 3.71 (m, 16H).

Data was consistent with those reported in the literature [7].

^{1}H NMR (400 MHz, $CHCl_3$-*d*, 298 K) spectrum of 19

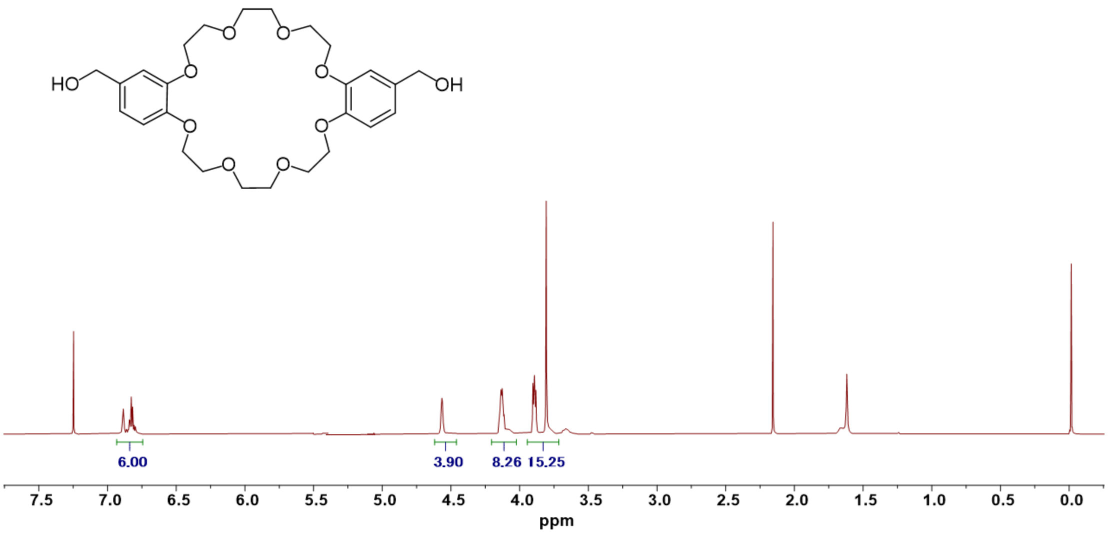

Synthesis of R

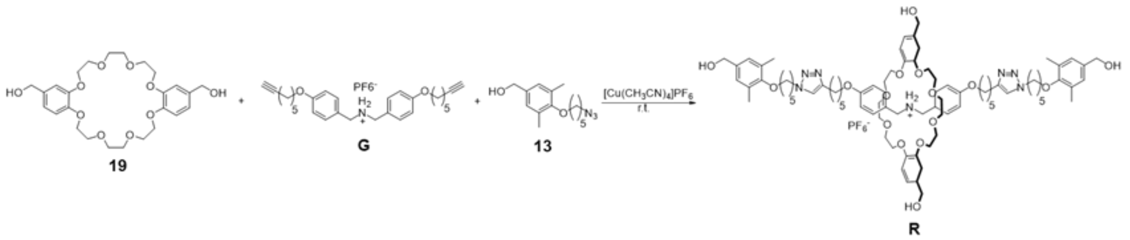

19 (0.50 g, 0.98 mmol), G (0.83 g, 1.47 mmol), and 13 (0.78 g, 2.95 mmol) were dissolved in dry and degassed CH_2Cl_2 (10 mL). After the solution was stirred for 1 h, $[Cu(CH_3CN)_4]PF_6$ (0.12 g, 0.32 mmol) were added under N_2 atmosphere. Stir the mixture overnight, then diluted with CH_2Cl_2 (50 mL) and wash with EDTA·2Na solution (aq, 0.1 M, 2 x) and H_2O (2 x), dried over $MgSO_4$ and evaporated. The crude was purified via the silica column chromatography (CH_2Cl_2/ Methanol = 100/0 – 95/5, gradient elution). R was isolated as white solid. (Yield: 0.52 g, 33%). ^{1}H NMR (400 MHz, $CHCl_3$-*d*) δ 7.38 (s, 2H), 7.15 (d, *J* = 8.3 Hz, 4H), 6.97 (s, 4H), 6.85 – 6.60 (m, 10H), 4.54 (d, *J* = 5.2 Hz, 8H), 4.44 (t, *J* = 6.8 Hz, 4H), 4.36 (t, *J* = 7.1 Hz, 4H), 4.16 – 3.98 (m, 8H), 3.88 (t, *J* = 6.7 Hz, 4H), 3.79 – 3.62 (m, 16H), 3.53-3.40 (m, 8H), 2.72 (t, *J* = 7.5 Hz, 4H), 2.21 (s, 12H), 2.06 – 1.90 (m, 4H), 1.76 (m, 12H), 1.53 (m, 8H). ^{13}C NMR (126 MHz, $CHCl_3$-*d*) δ 159.52, 155.41, 147.41, 146.75, 136.20, 131.06, 130.67, 127.78, 123.59, 120.14, 114.72, 112.38, 111.80, 71.81, 70.86, 70.65, 70.33, 70.12, 69.08, 68.29, 68.09, 67.95, 65.11, 64.75, 52.02, 30.27, 29.86, 28.75, 25.50, 23.38, 16.40. HR-MS (ESI): calcd for , $[C_{98}H_{140}F_6N_7O_{19}P\text{-}PF_6]^+$, m/z = 1482.8792, found m/z = 1482.8790.

^{1}H NMR (400 MHz, $CHCl_3$-*d*, 298 K) spectrum of R

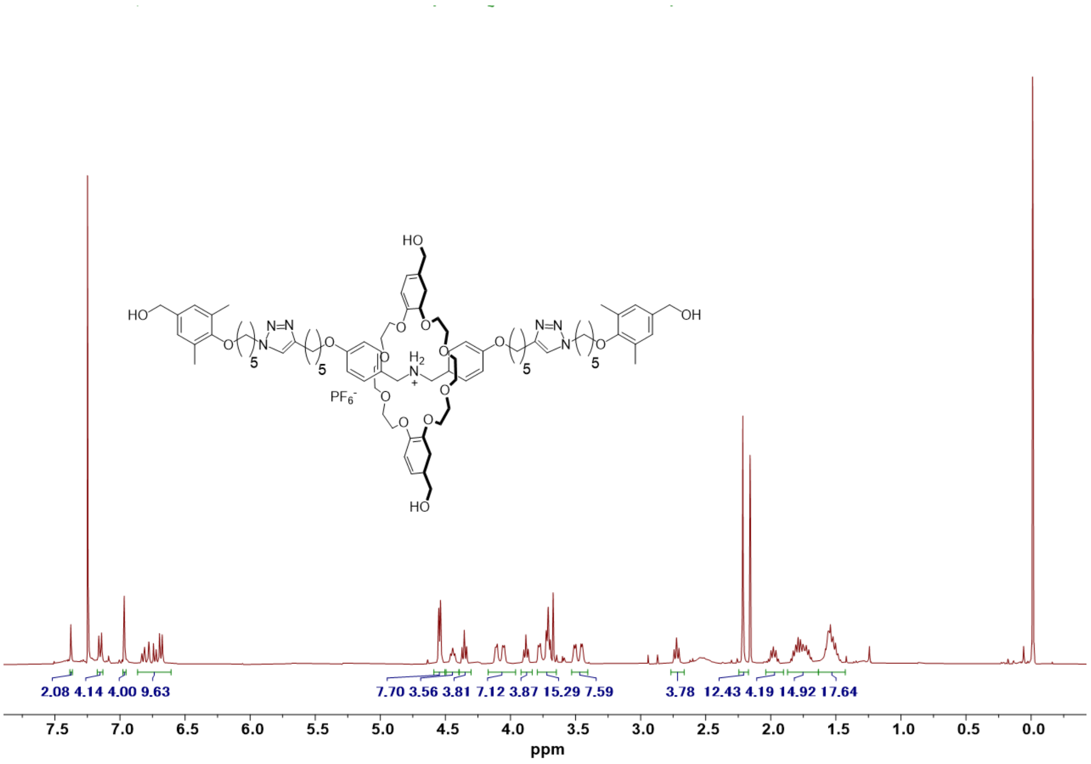

^{13}C NMR (126 MHz, $CHCl_3$-*d*, 298 K) spectrum of R

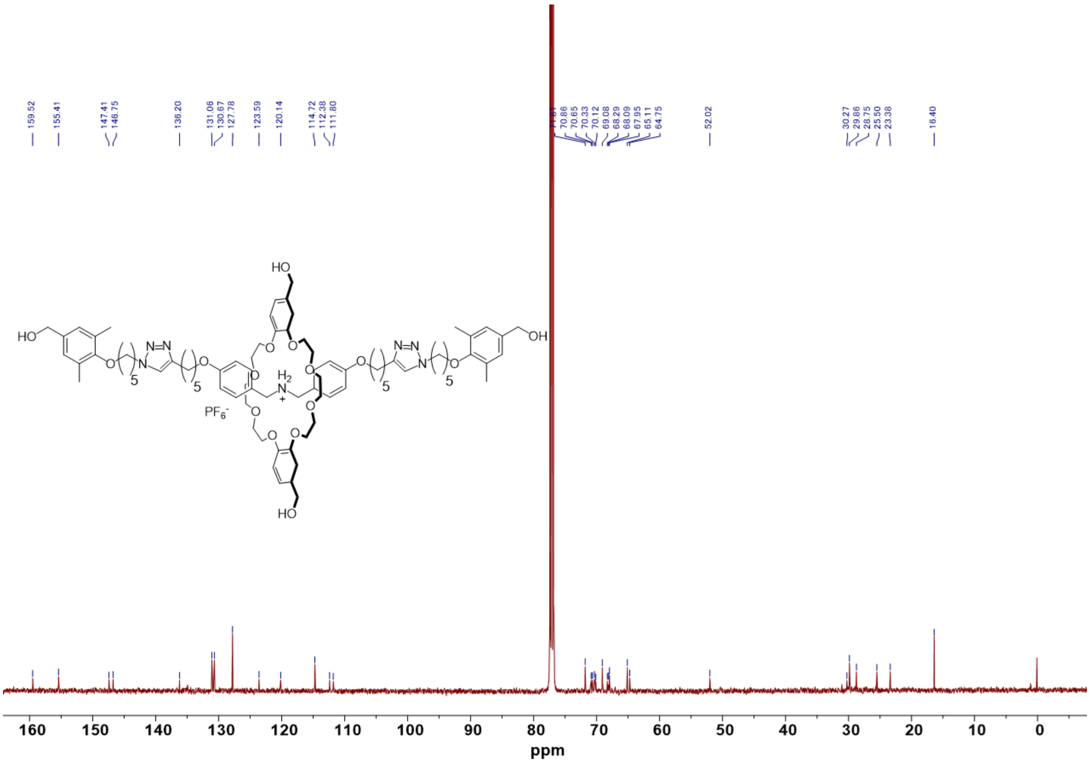

2. Preparation of polymers

Synthesis of PU-RM

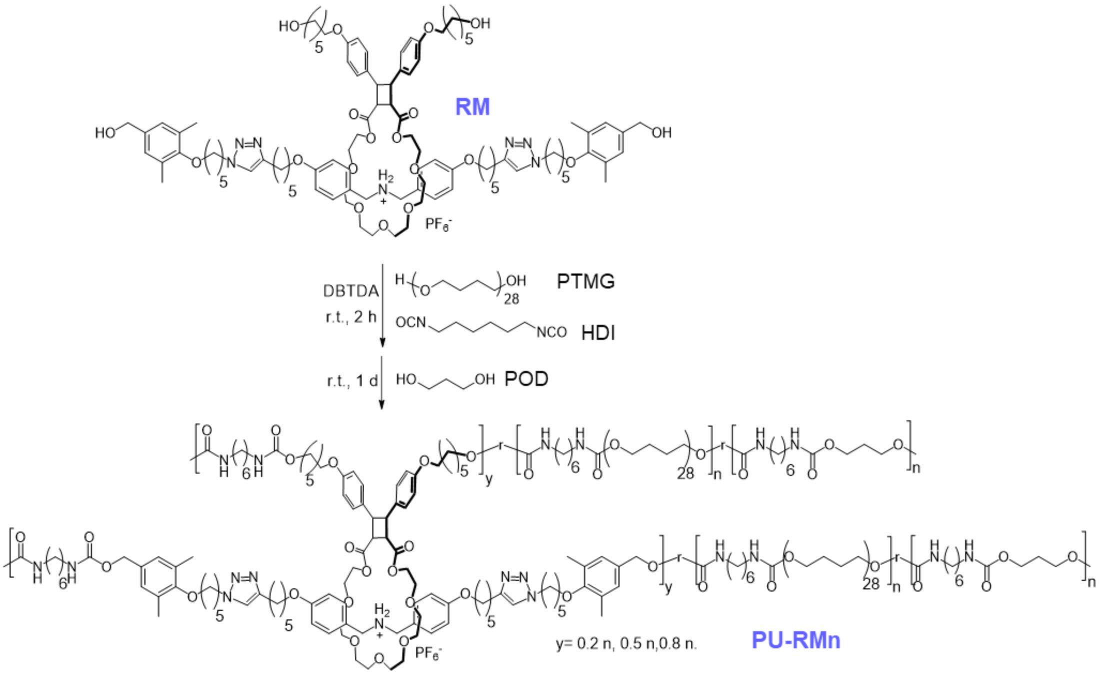

RM (molar ratio 1.0 %, 2.5 %, 4.0%), Poly(tetrahydrofuran) (PTMG, M_n = 2000, 2.00 g, 1.00 mmol) dissolved in dry CH_2Cl_2 (25 mL). 1,6-Diisocyanatohexane (HDI, 0.36 g, 2.13 mmol) and Dibutyltin diacetate (DBTDA, 2.50 mg) in dry CH_2Cl_2 (5 mL) were added to the mixture under N_2 atmosphere. The mixture was stirred at room temperature for 2 h. 1,3-Propanediol (POD, 76.0 mg, 1.00 mmol) dissolved in dry CH_2Cl_2 (2 mL) was added into the mixture followed by further stirring for 22 h. The reaction mixture was added to hexane to precipitate the polymer. The collected precipitate was dissolved in CH_2Cl_2 and poured into a Teflon mold and dried at 25 °C overnight to afford the PU-RM1-3 film (yield from 86% to 95%).

Synthesis of PU-MCM

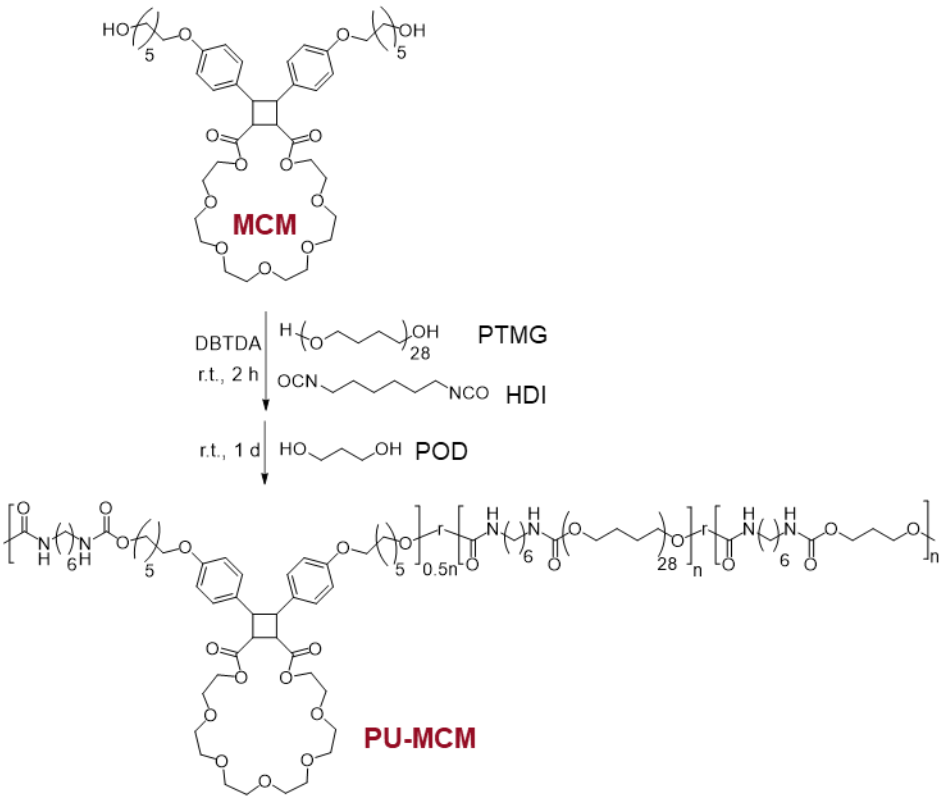

MCM (38.7 mg, 0.05 mmol), PTMG (M_n = 2000, 2.00 g, 1.00 mmol) dissolved in dry CH_2Cl_2 (25 mL). HDI (0.35 g, 2.08 mmol) and DBTDA (2.50 mg) in dry CH_2Cl_2 (5 mL) were added to the mixture under N_2 atmosphere. The mixture was stirred at room temperature for 2 h. POD (76.0 mg, 1.00 mmol) dissolved in dry CH_2Cl_2 (2 mL) was added into the mixture followed by further stirring for 22 h. The reaction mixture was added to hexane to precipitate the polymer. The collected precipitate was dissolved in CH_2Cl_2 and poured into a Teflon mold and dried at 25 °C overnight to afford the PU-MCM film (yield: 2.33 g, 94%).

Synthesis of PU-C

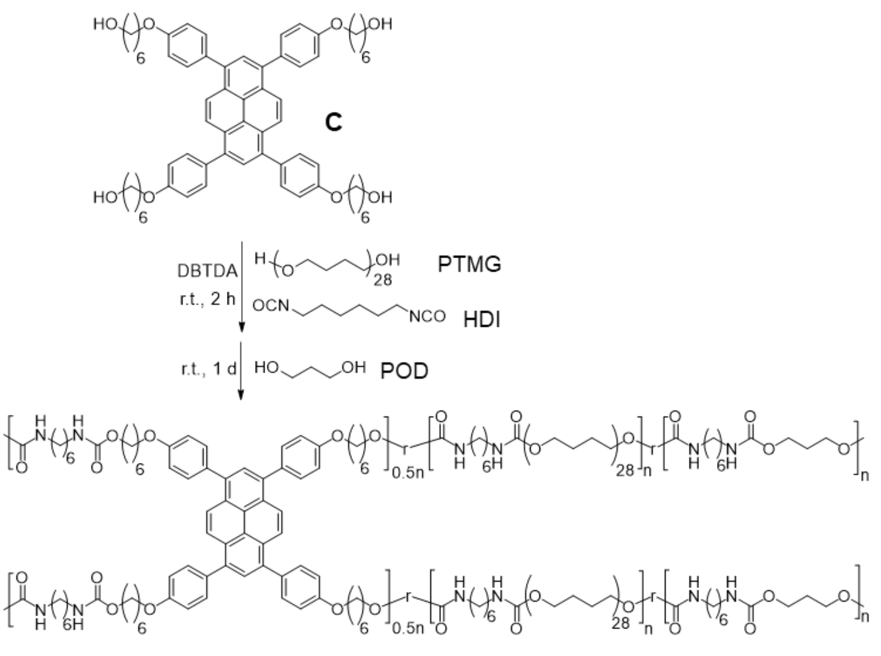

C (48.6 mg, 0.05 mmol), PTMG (M_n = 2000, 2.00 g, 1.00 mmol) dissolved in dry CH_2Cl_2 (25 mL). HDI (0.36 g, 2.13 mmol) and DBTDA (2.50 mg) in dry CH_2Cl_2 (5 mL) were added to the mixture under N_2 atmosphere. The mixture was stirred at room temperature for 2 h. POD (76.0 mg, 1.00 mmol) dissolved in dry CH_2Cl_2 (2 mL) was added into the mixture followed by further stirring for 22 h. The reaction mixture was added to hexane to precipitate the polymer. The collected precipitate was dissolved in CH_2Cl_2 and poured into a Teflon mold and dried at 25 °C overnight to afford the PU-C film (yield: 2.29 g, 93%).

Synthesis of PU-R

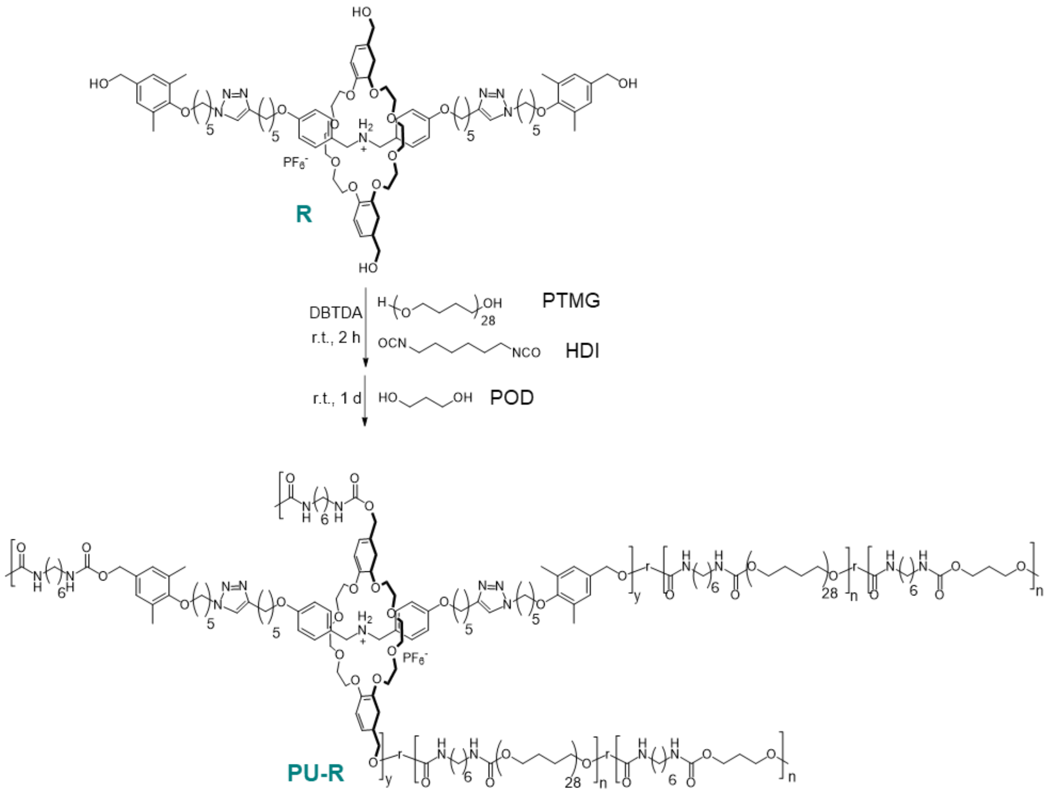

R (79.9 mg, 0.05 mmol), PTMG (M_n = 2000, 2.00 g, 1.00 mmol) dissolved in dry CH_2Cl_2 (25 mL). HDI (0.36 g, 2.13 mmol) and DBTDA (2.50 mg) in dry CH_2Cl_2 (5 mL) were added to the mixture under N_2 atmosphere. The mixture was stirred at room temperature for 2 h. POD (76.0 mg, 1.00 mmol) dissolved in dry CH_2Cl_2 (2 mL) was added into the mixture followed by further stirring for 22 h. The reaction mixture was added to hexane to precipitate the polymer. The collected precipitate was dissolved in CH_2Cl_2 and poured into a Teflon mold and dried at 25 °C overnight to afford the PU-R film (yield: 2.39 g, 95%).

Synthesis of PU-CA

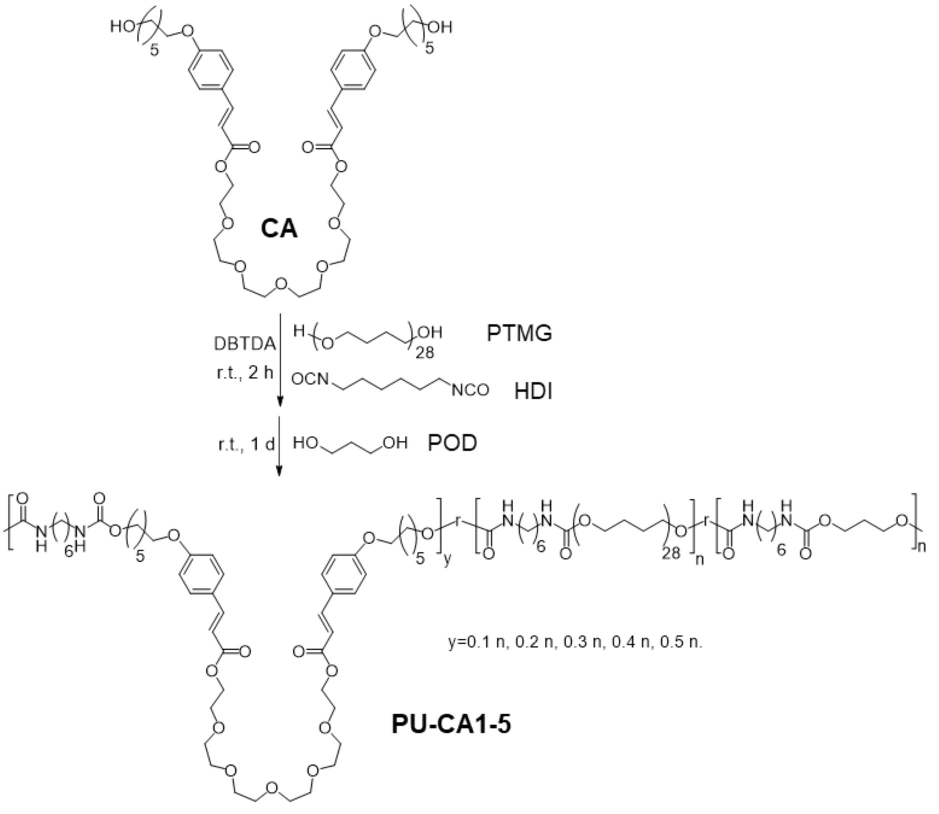

5 (molar ratio 0.5%, 1.0 %, 1.5%, 2.0%, 2.5 %), PTMG (M_n = 2000, 2.00 g, 1.00 mmol) dissolved in dry CH_2Cl_2 (25 mL). HDI (0.36 g, 2.13 mmol) and DBTDA (2.50 mg) in dry CH_2Cl_2 (5 mL) were added to the mixture under N_2 atmosphere. The mixture was stirred at room temperature for 2 h. POD (76.00 mg, 1.00 mmol) dissolved in dry CH_2Cl_2 (2 mL) was added into the mixture followed by further stirring for 22 h. The reaction mixture was added to hexane to precipitate the polymer. The collected precipitate was dissolved in CH_2Cl_2 and poured into a Teflon mold and dried at 25 °C overnight to afford the PU-CA1-5 film with different molar ratio of cinnamate moiety (CA) of 0.5%, 1.0 %, 1.5%, 2.0%, and 2.5 %, respectively (yield from 84% to 95%).

3. Structural analysis of RM

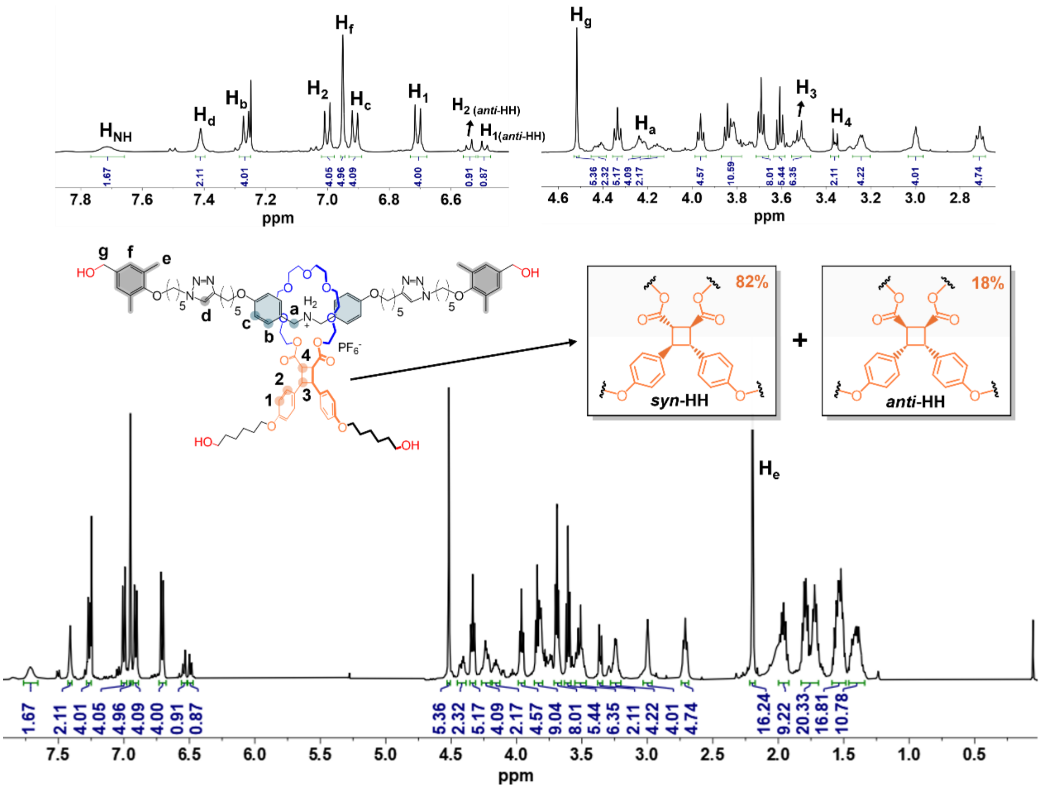

Supplementary Figure 8. ^{1}H NMR spectrum of **RM**; 500 Hz, 298 K, $CHCl_3$-*d*.

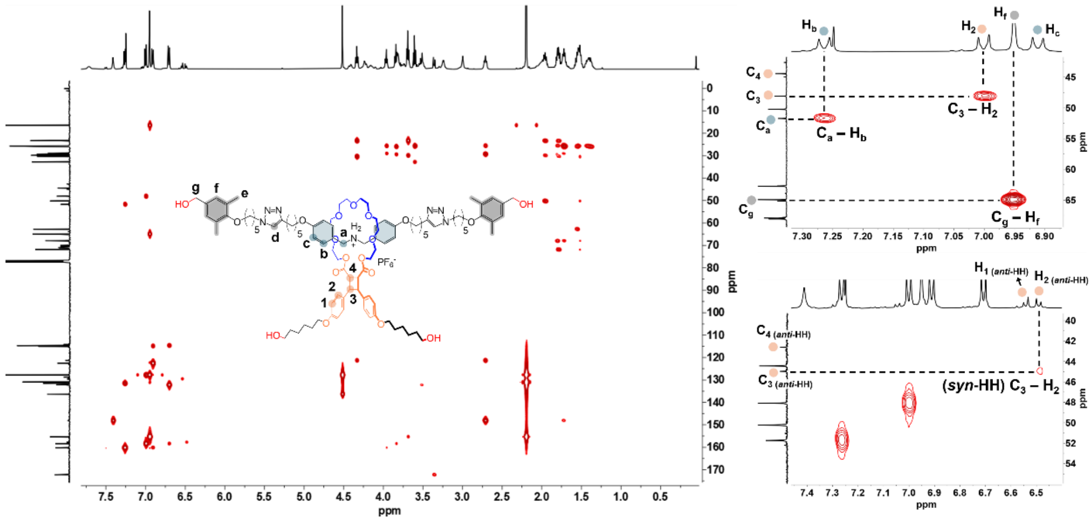

Supplementary Figure 9. HMBC spectrum of RM; 500 Hz, 298 K, $CHCl_3$-*d*.

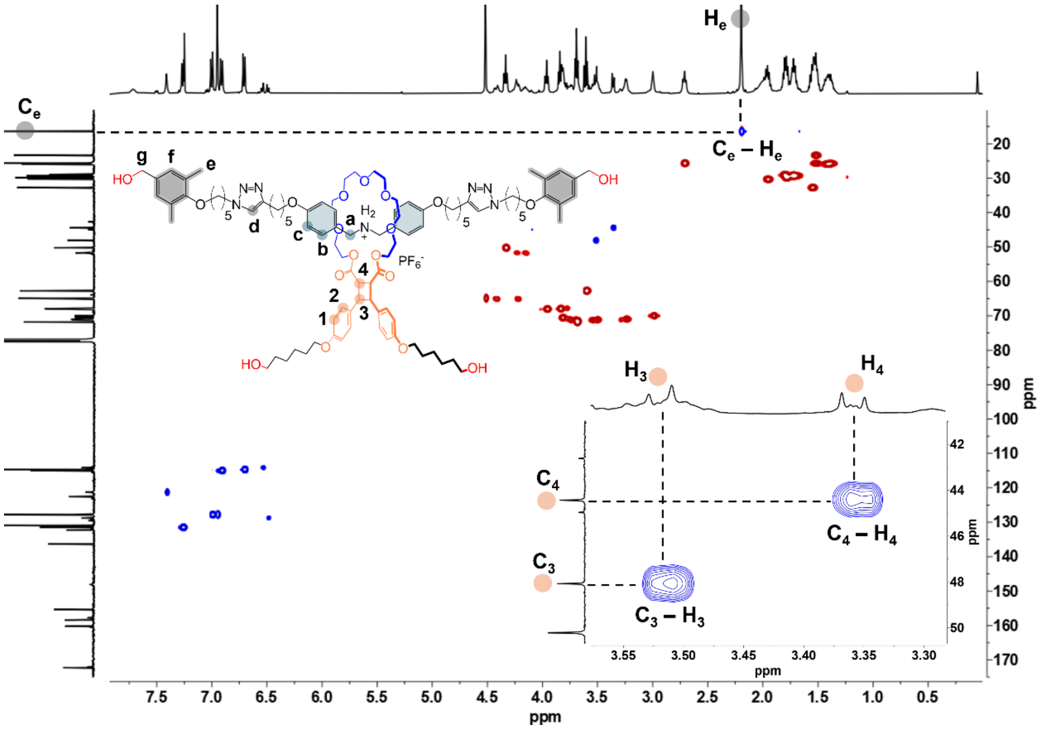

Supplementary Figure 10. HSQC spectrum of RM; 500 Hz, 298 K, $CHCl_3$-*d*.

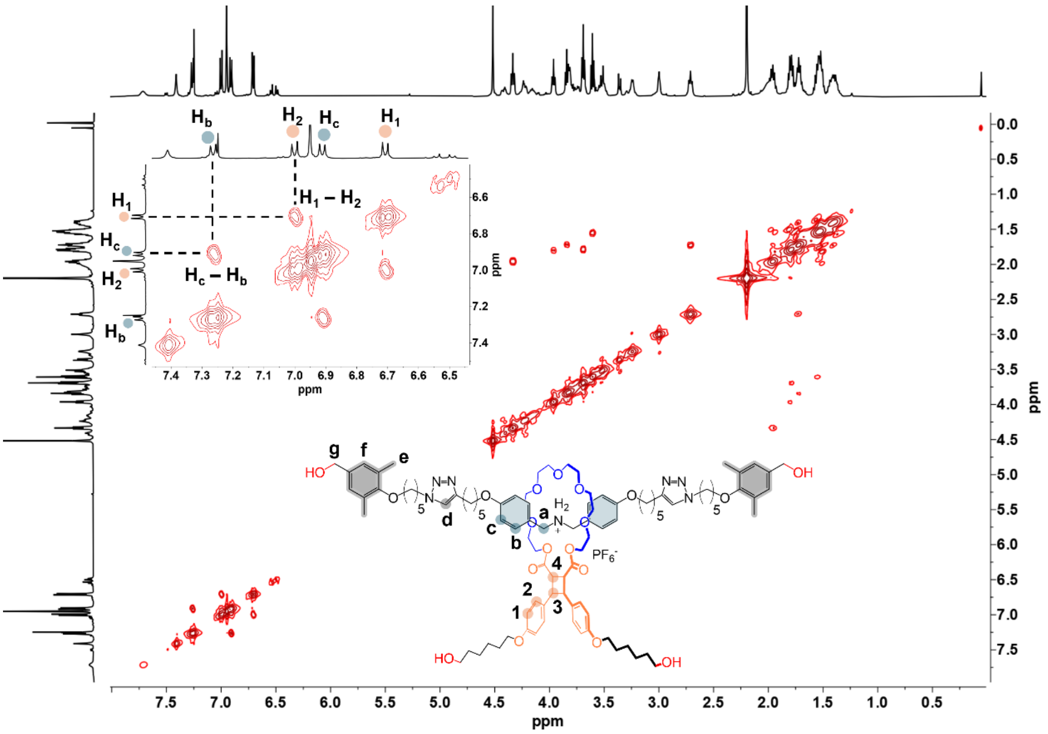

Supplementary Figure 11. COSY spectrum of RM; 500 Hz, 298 K, $CHCl_3$-*d*.

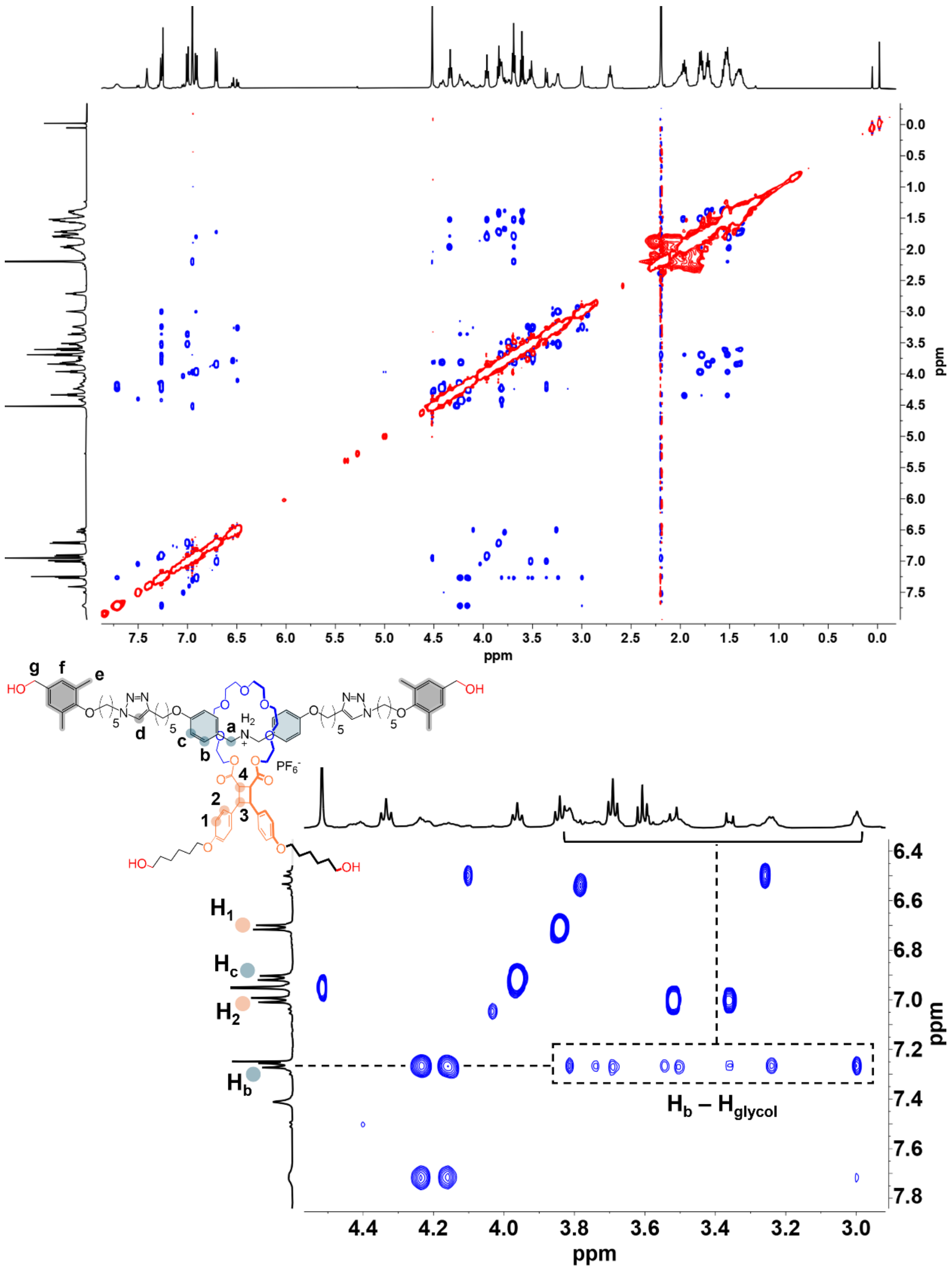

Supplementary Figure 12. ROESY spectrum of RM; 600 Hz, 298 K, $CHCl_3$-*d*.

4. Structural characterization of polymers

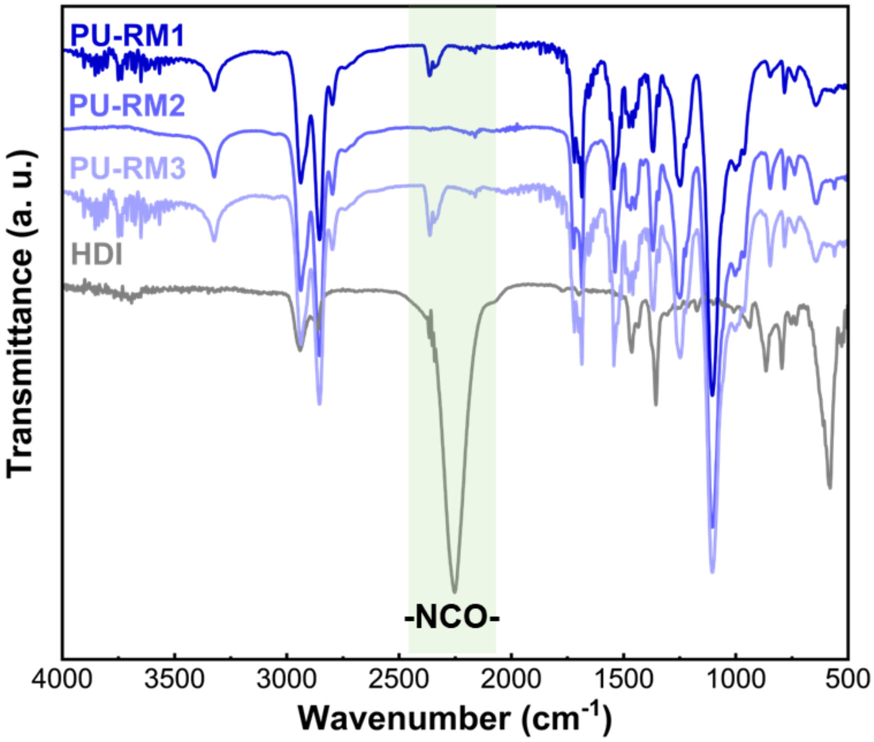

Supplementary Figure 13. FTIR spectra of PU-RM1-3. ATR-FTIR spectrum of HDI was shown in Figure as a reference.

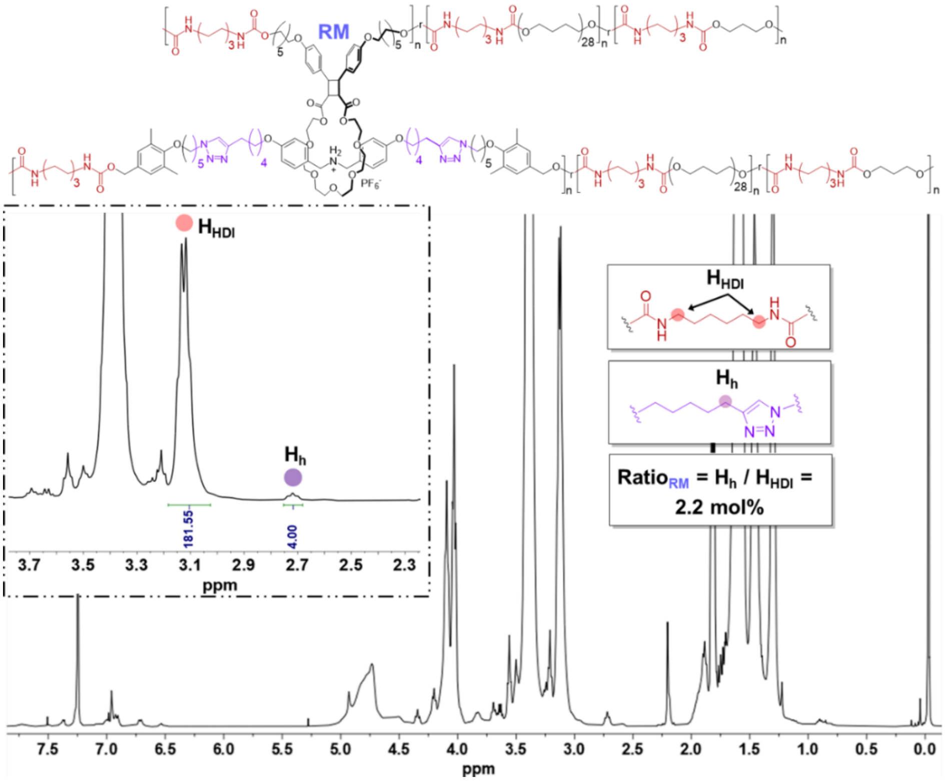

Supplementary Figure 14. ^{1}H NMR spectrum of PU-RM2; 500 Hz, 298 K, $CHCl_3$-*d*.

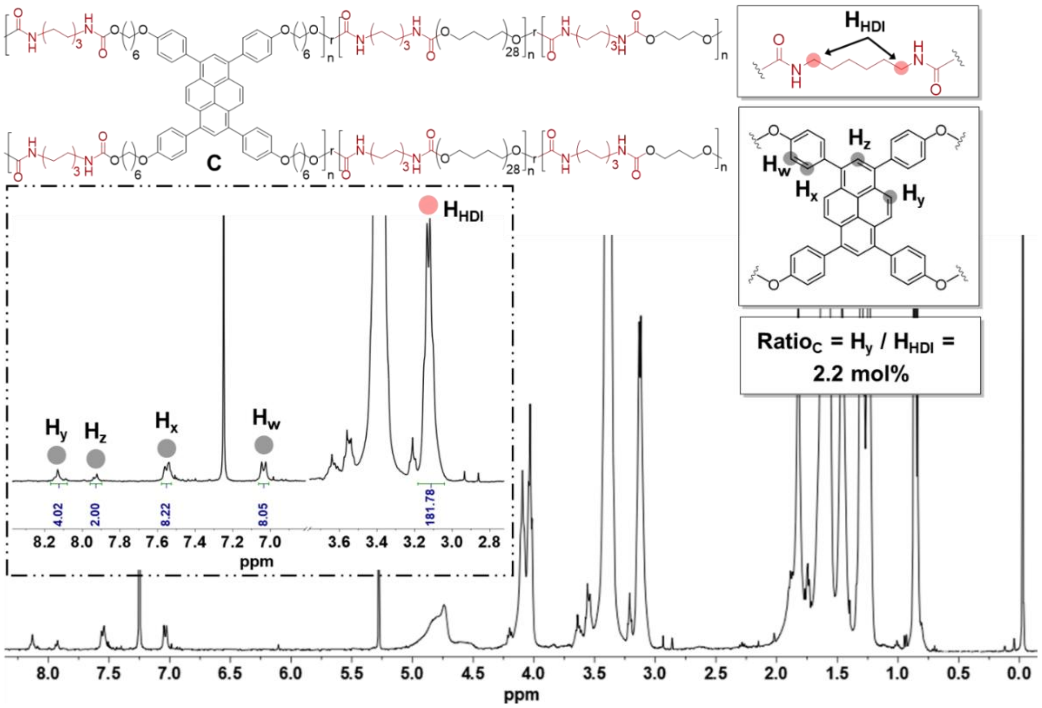

Supplementary Figure 15. ^{1}H NMR spectrum of PU-C; 500 Hz, 298 K, $CHCl_3$-*d*.

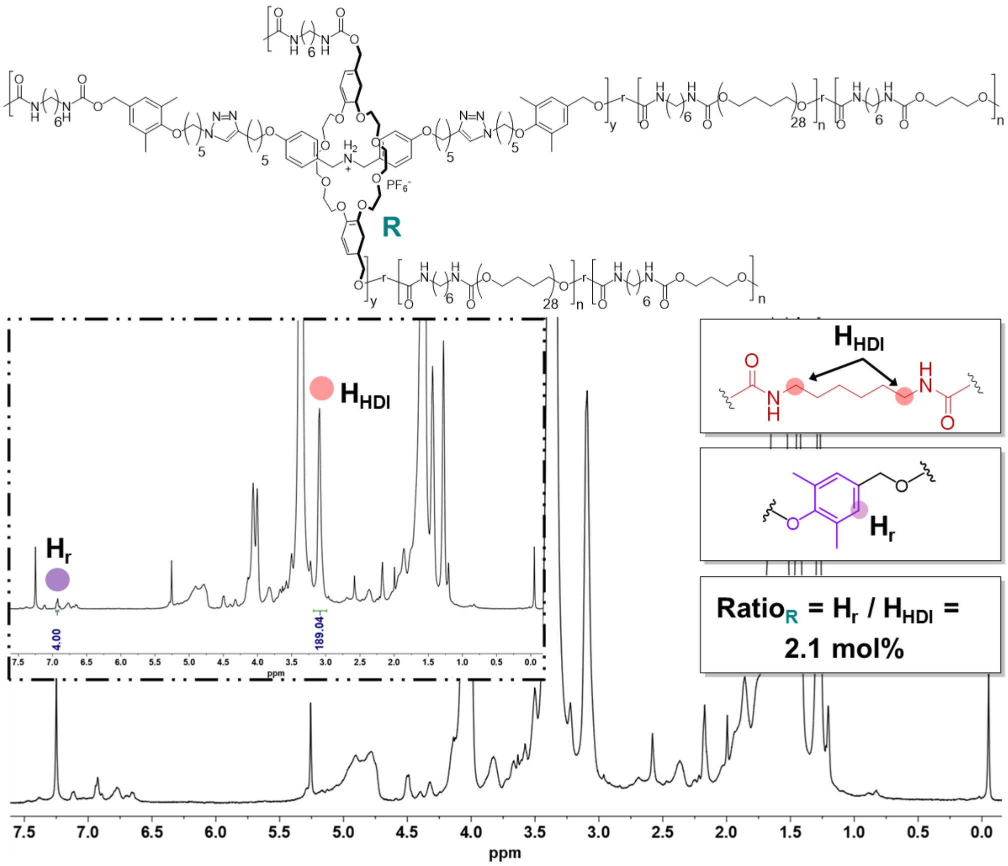

Supplementary Figure 16. ^1H NMR spectrum of PU-R; 500 Hz, 298 K, $CHCl_3$-*d*.

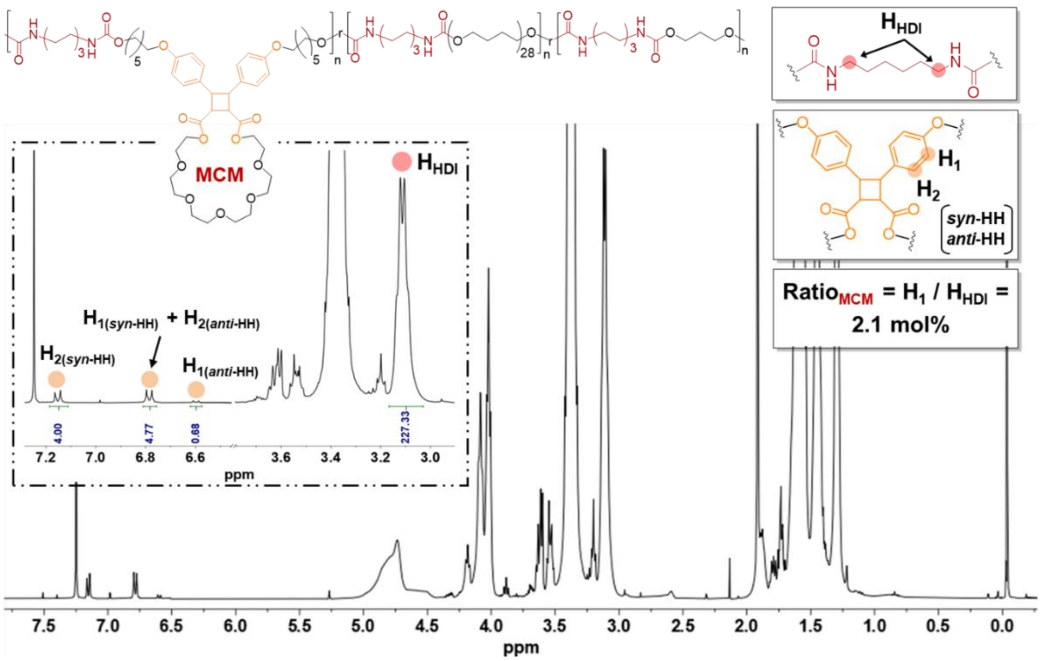

Supplementary Figure 17. ^1H NMR spectrum of PU-MCM; 500 Hz, 298 K, $CHCl_3$-*d*.

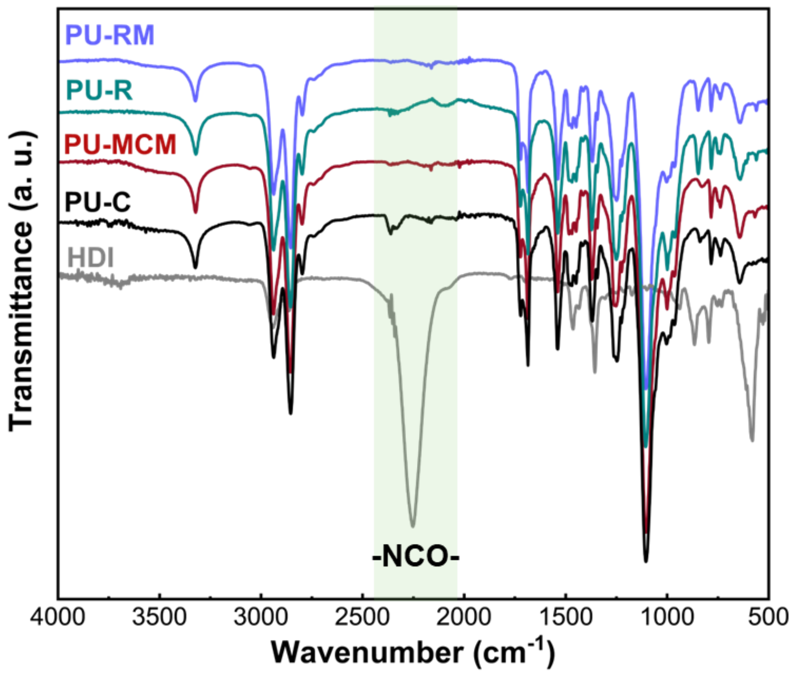

Supplementary Figure 18. FTIR spectra of PU-RM2, PU-R, PU-MCM, and PU-C. FTIR spectrum of HDI was shown in Figure as a reference.

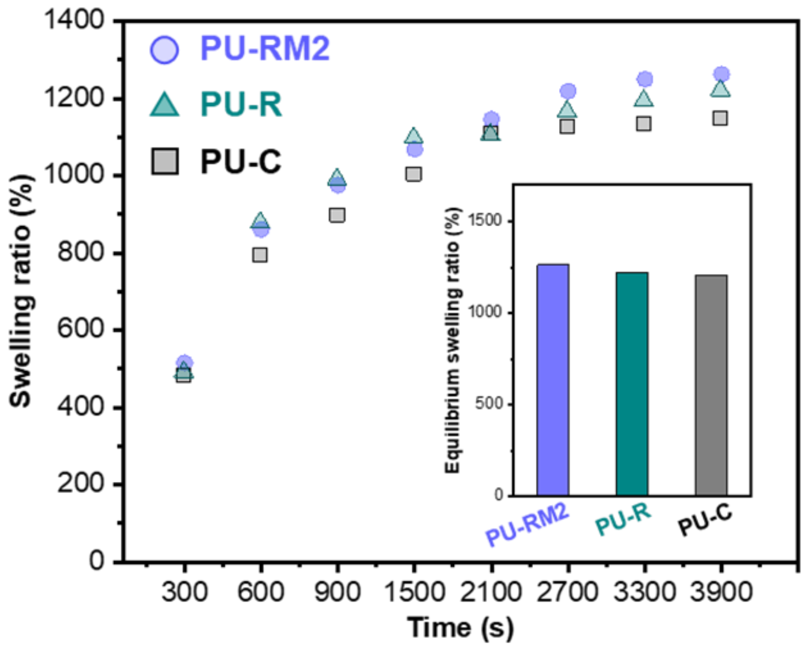

Supplementary Figure 19. Swelling ratio curves of PU-RM2, PU-R and PU-C in THF at room temperature.

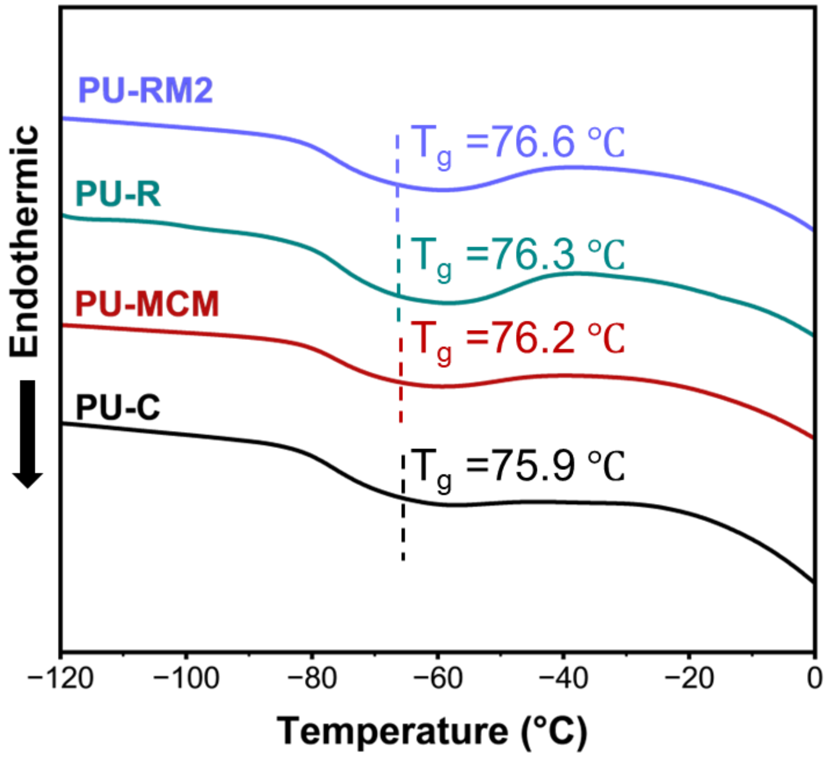

Supplementary Figure 20. DSC curves of 2nd scan for PU-RM2, PU-R, PU-MCM, and PU-C. The temperature range was from -150 °C to 0 °C, the heating rate was 10 °C/min.

5. Mechanical properties of PU-RM and controls

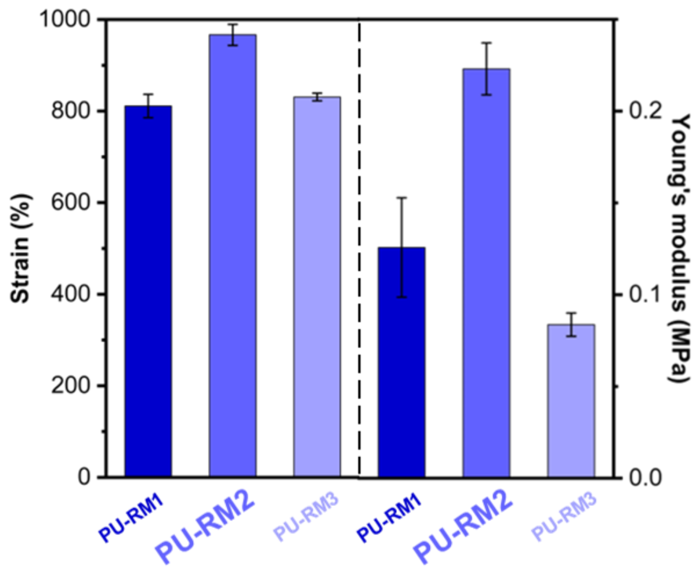

Supplementary Figure 21. Fracture strain and Young's modulus of PU-RM1-3 calculated from their stress-strain curves. Values represent the mean and the standard deviation calculated from the mechanical property data obtained from three independent measurements.

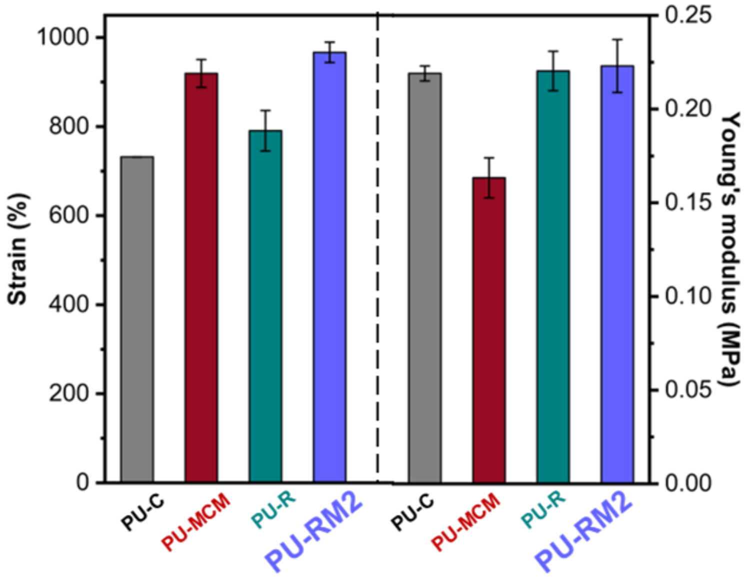

Supplementary Figure 22. Fracture strain and Young's modulus of PU-RM2, PU-R, PU-MCM, and PU-C. calculated from their stress-strain curves. Values represent the mean and the standard deviation calculated from the mechanical property data obtained from three independent measurements.

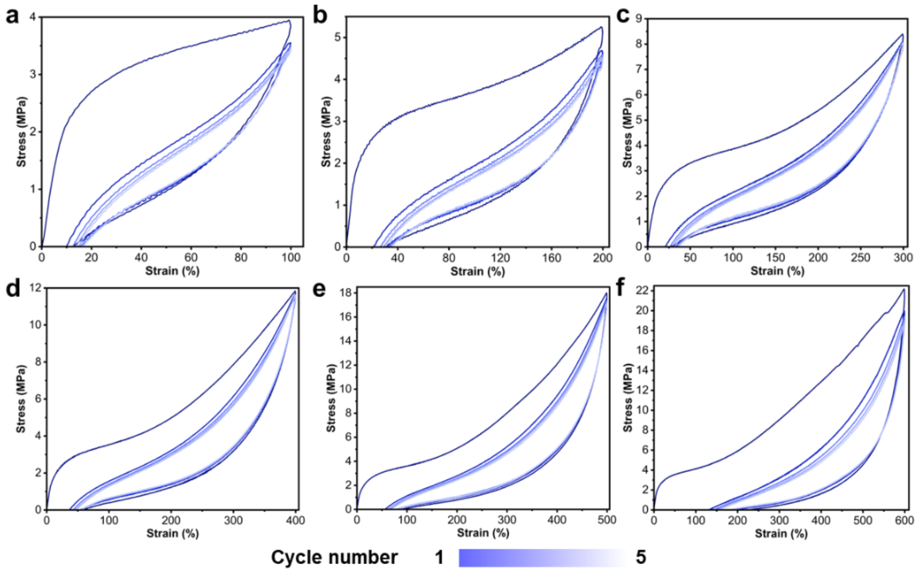

Supplementary Figure 23. Tensile stress-strain curves with five successive loading-unloading cycles for PU-RM2 at a maximum strain of 100% a), 200% b), 300% c), 400% d), 500% e), and 600% f).

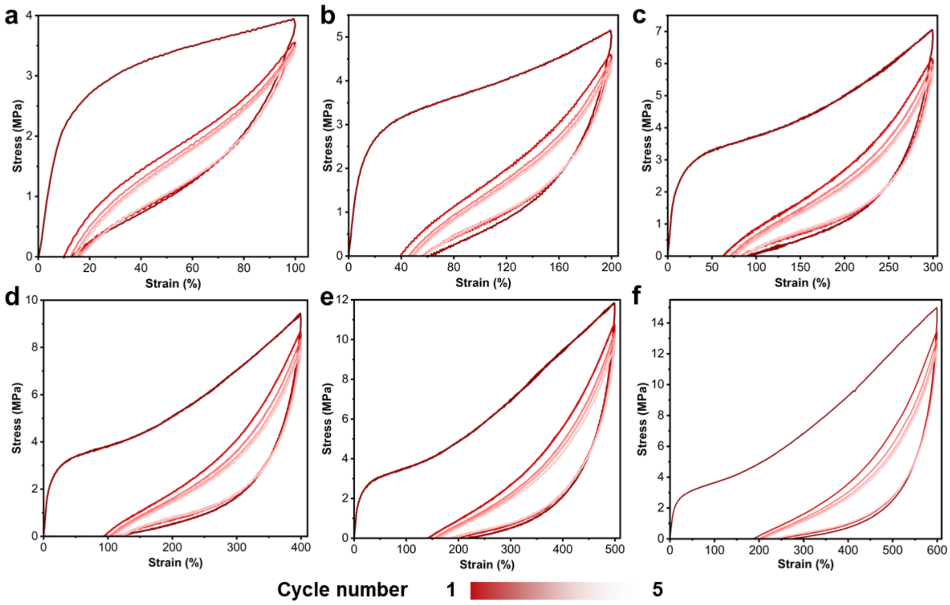

Supplementary Figure 24. Tensile stress-strain curves with five successive loading-unloading cycles for PU-MCM at a maximum strain of 100% a), 200% b), 300% c), 400% d), 500% e), and 600% f).

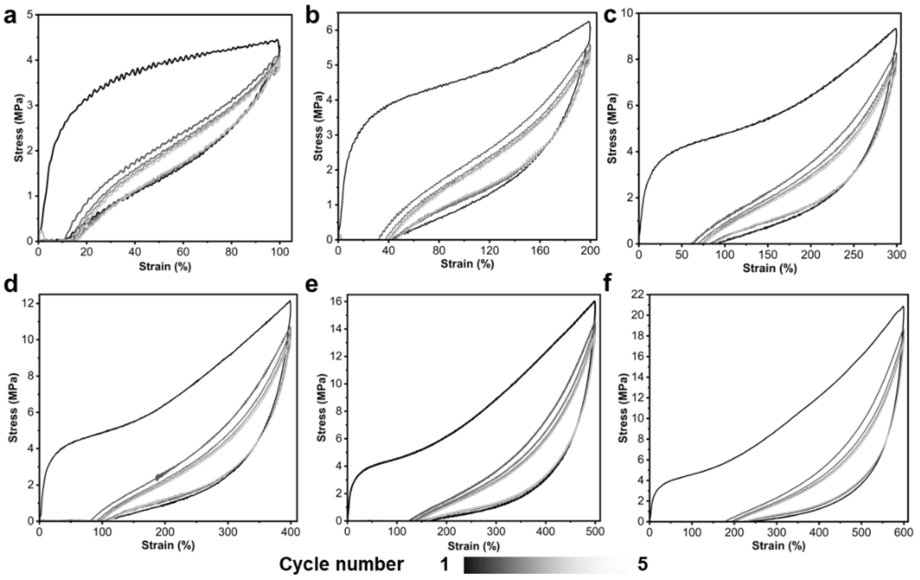

Supplementary Figure 25. Tensile stress-strain curves with five successive loading-unloading cycles for PU-C at a maximum strain of 100% a), 200% b), 300% c), 400% d), 500% e), and 600% f).

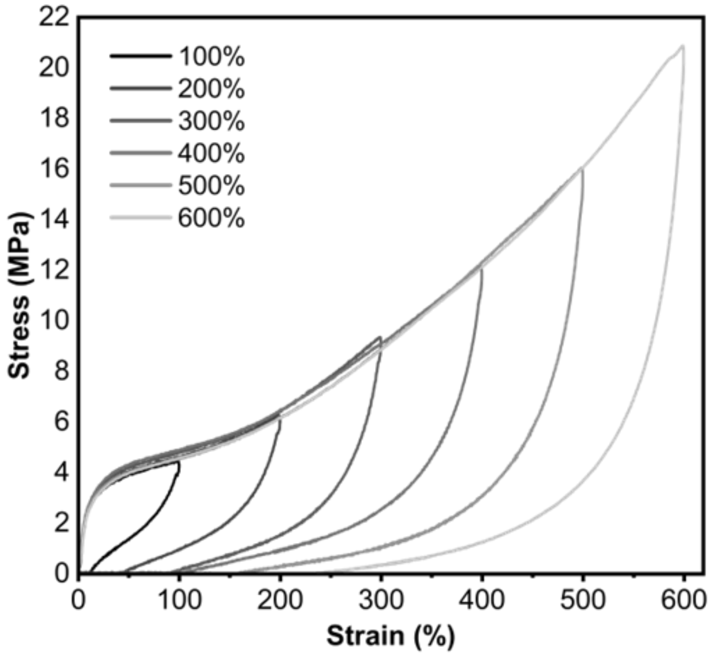

Supplementary Figure 26. Cyclic tensile curves of PU-C recorded at room temperature with increased maximum strain.

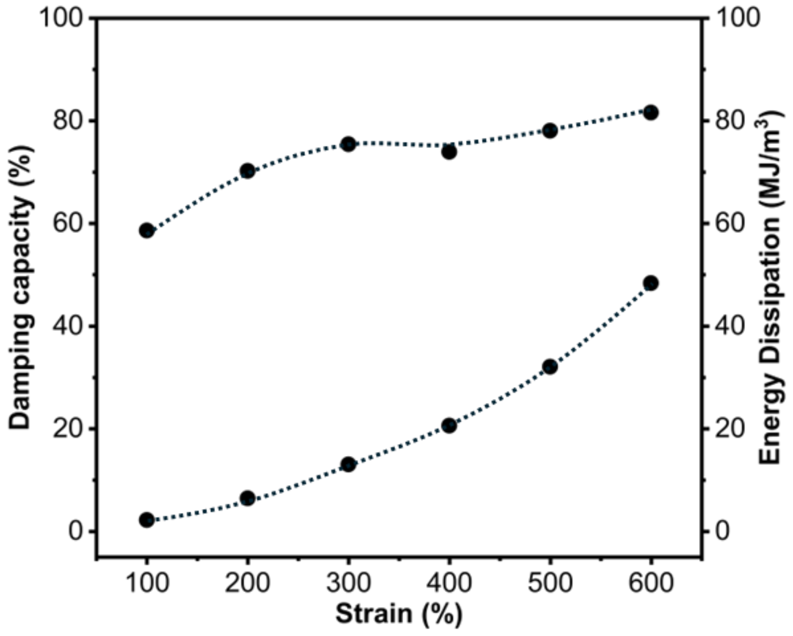

Supplementary Figure 27. Damping capacity (the ratio of dissipated energy to total input energy) and hysteresis area of PU-C for each cycle in the cyclic tensile tests.

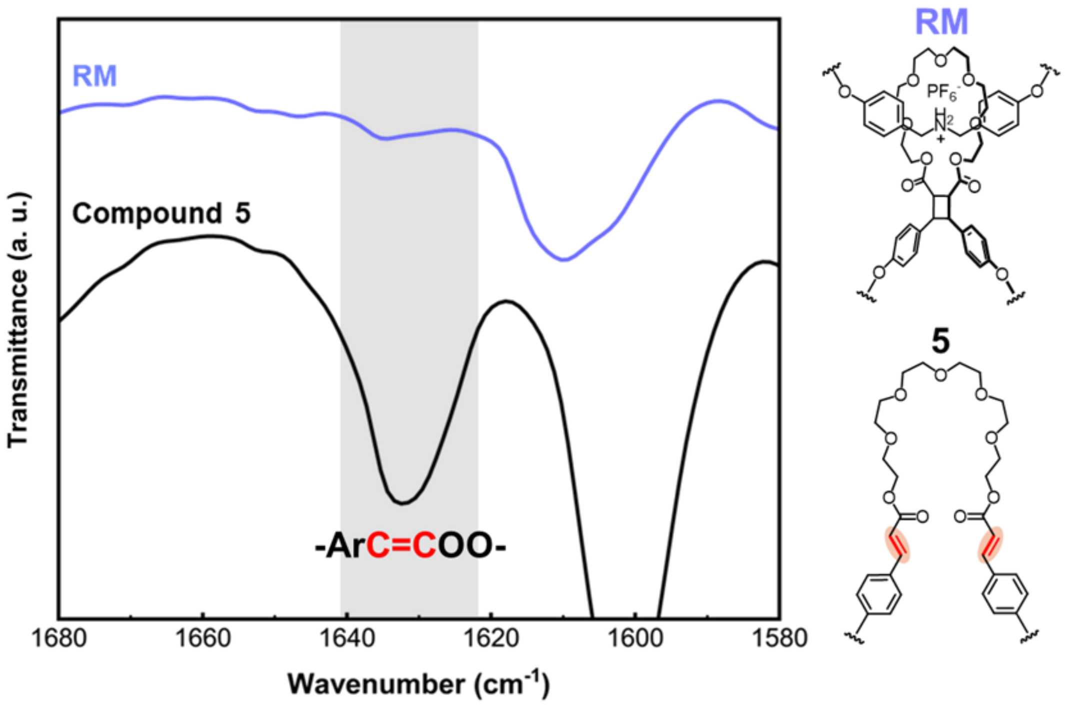

Supplementary Figure 28. Partial FTIR spectra of RM and compound 5.

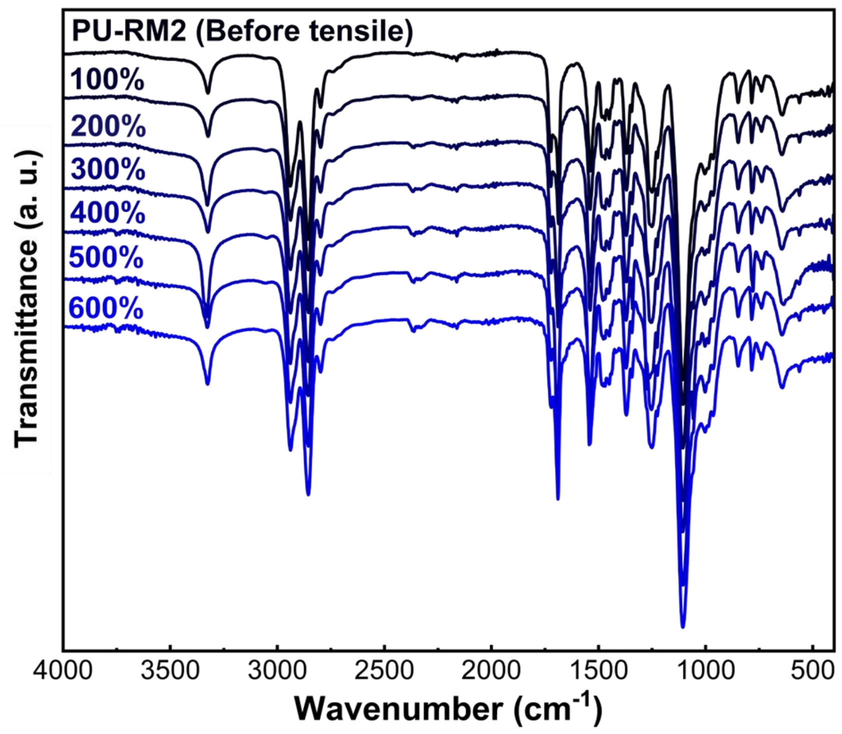

Supplementary Figure 29. FTIR spectra of PU-RM2 after tensile with stain interval.

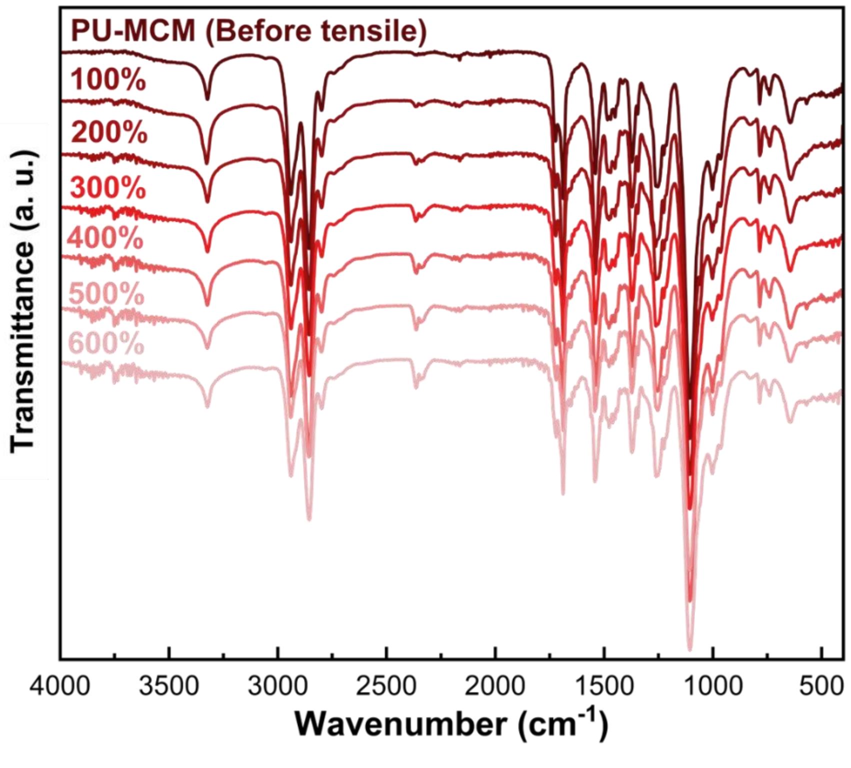

Supplementary Figure 30. FTIR spectra of PU-MCM after tensile with stain interval.

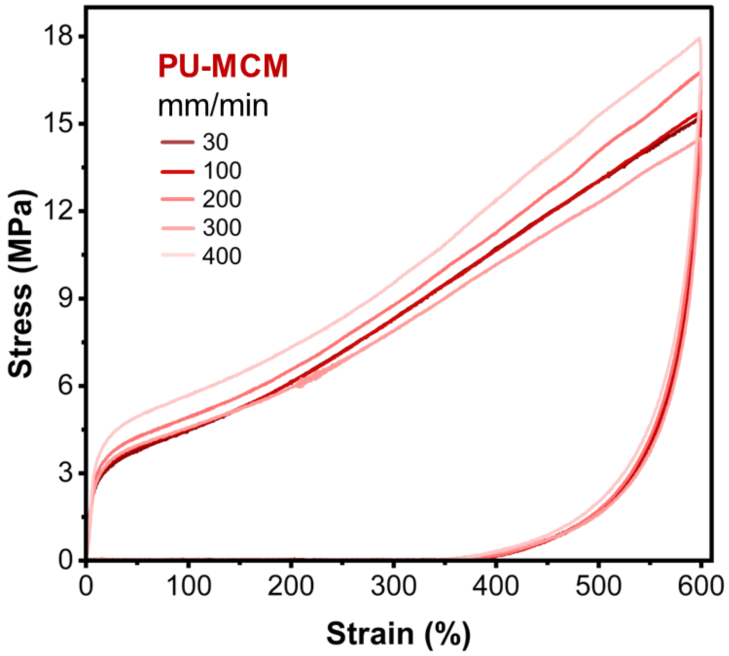

Supplementary Figure 31. Cyclic tensile experiments of PU-MCM at 600% strain recorded with deformation rates ranging from 30 to 400 mm/min.

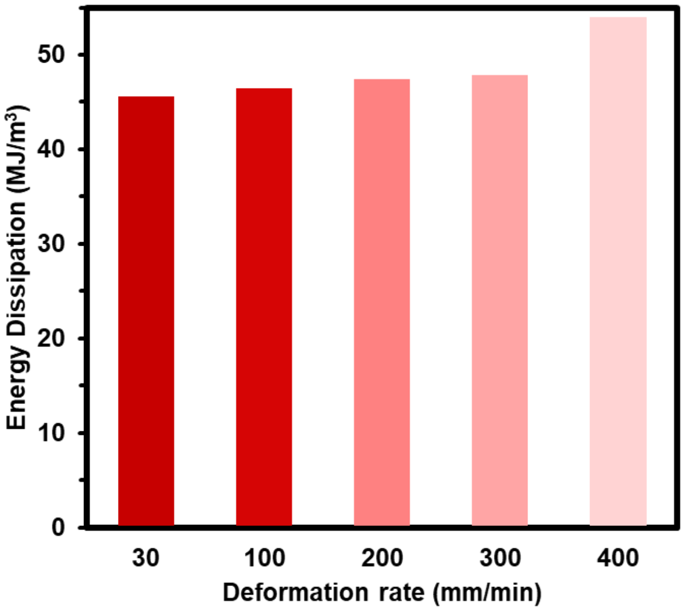

Supplementary Figure 32. Energy dissipation of PU-MCM at various deformation rates.

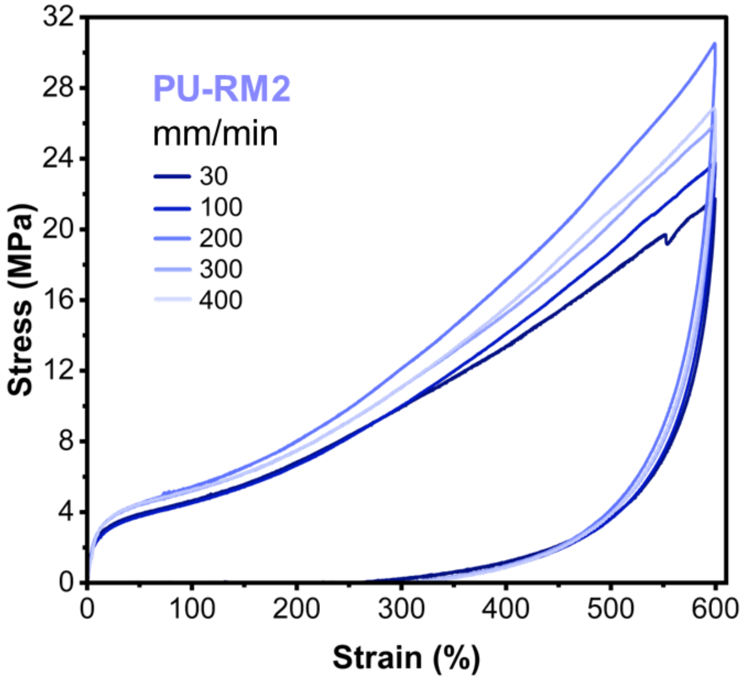

Supplementary Figure 33. Cyclic tensile experiments of PU-RM2 recorded with deformation rates ranging from 30 to 400 mm/min.

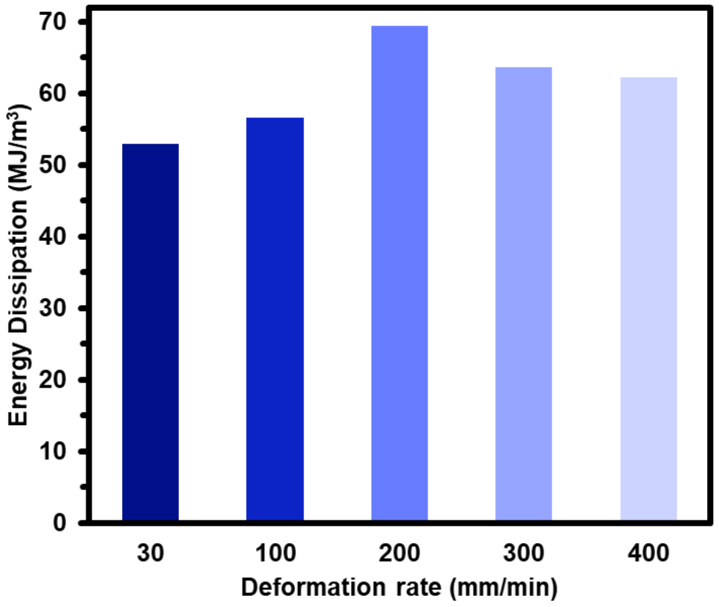

Supplementary Figure 34. Energy dissipation of PU-RM2 at various deformation rates.

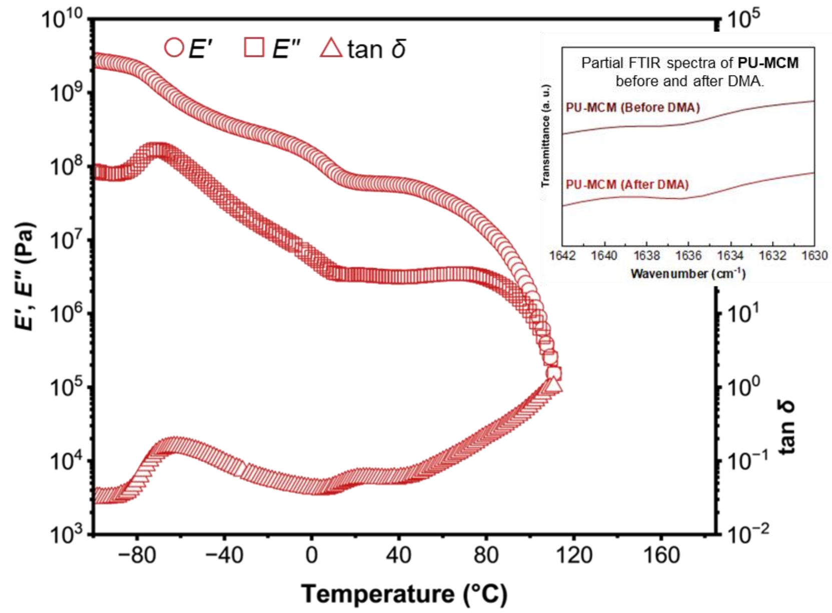

Supplementary Figure 35. Temperature sweep curves for PU-MCM spanning a temperature range from −100 to 120 °C at a frequency of 8.0 Hz.

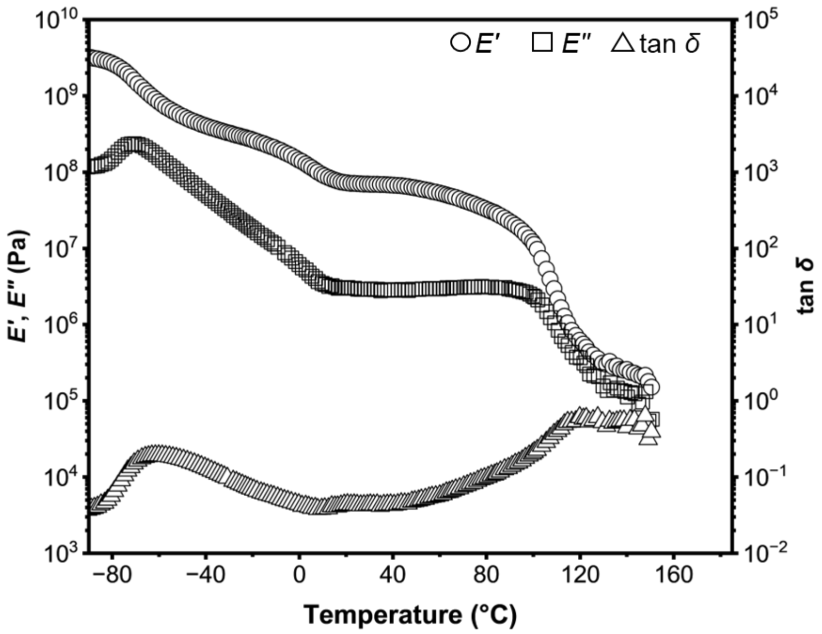

Supplementary Figure 36. Temperature sweep curves for PU-C spanning a temperature range from −100 to 160 °C at a frequency of 8.0 Hz.

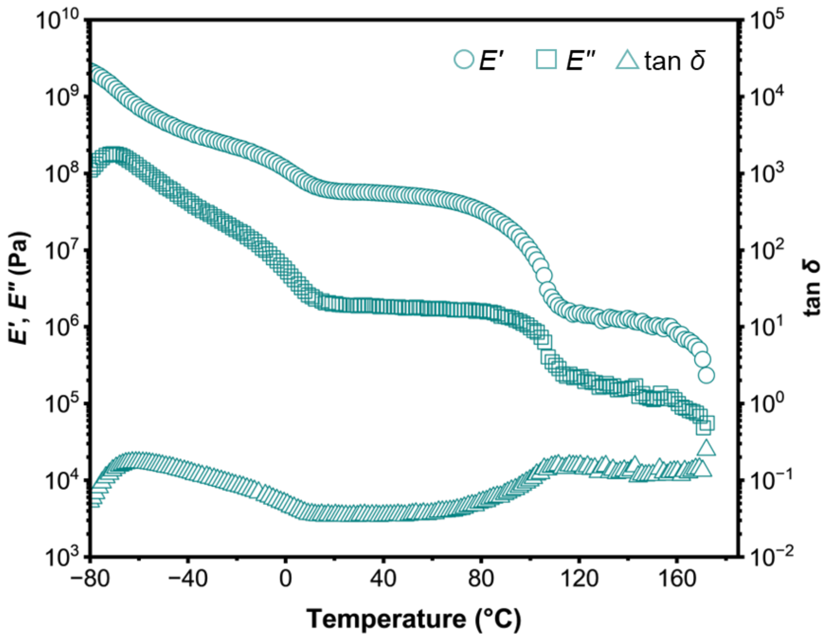

Supplementary Figure 37. Temperature sweep curves for PU-R spanning a temperature range from −80 to 180 °C at a frequency of 8.0 Hz.

6. Rupture Ratio Evaluation Based on FTIR Measurements

To evaluate the structural change quantitatively, FTIR spectra of the samples were recorded by ATR mode. The peaks at 1636 and 1465 cm^{-1} were selected, where the band at 1636 cm^{-1} corresponds to C=C and the band at 1465 cm^{-1} (CH_2 bending) was used as an internal reference [8, 9]. The peak areas were obtained by integrating the absorbance over 1632–1640 cm^{-1} and 1463–1470 cm^{-1}, respectively, using JASCO spectrum manager Ver.2. The absorbance ratio was then calculated according to

$$A_{r\,(PU-CA)} = \frac{A_{1636}}{A_{1465}}$$

where A_{1636} and A_{1465} refer to the integrated absorbance areas at 1636 and 1465 cm^{-1}, respectively. This ratio was used as an index of the relative amount of cinnamate moiety.

As shown in figure S31, the molar ratio of cinnamate moiety exhibits a clear proportional relationship with the FTIR absorbance area ratio, indicating that the amount of cinnamate moiety can be reliably quantified through this method. Therefore, this proportionality allows the rupture ratio to be calculated based on the absorbance ratio.

The absorbance areas ratio ($A_{r(DMA)}$) of PU-RM2 after DMA temperature measurements was calculated using the same method.

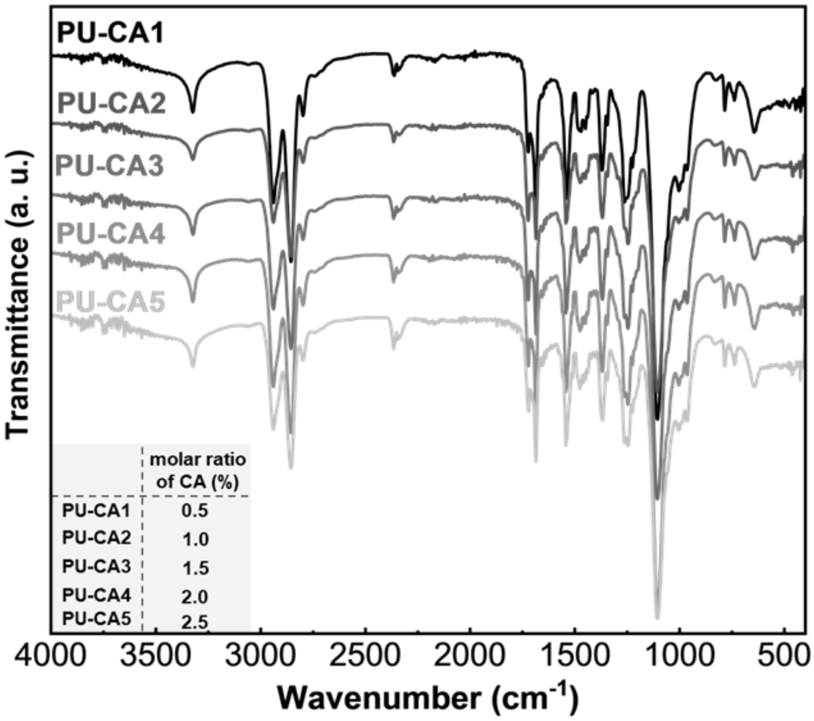

Supplementary Figure 38. FTIR spectra of PU-CA1-5.

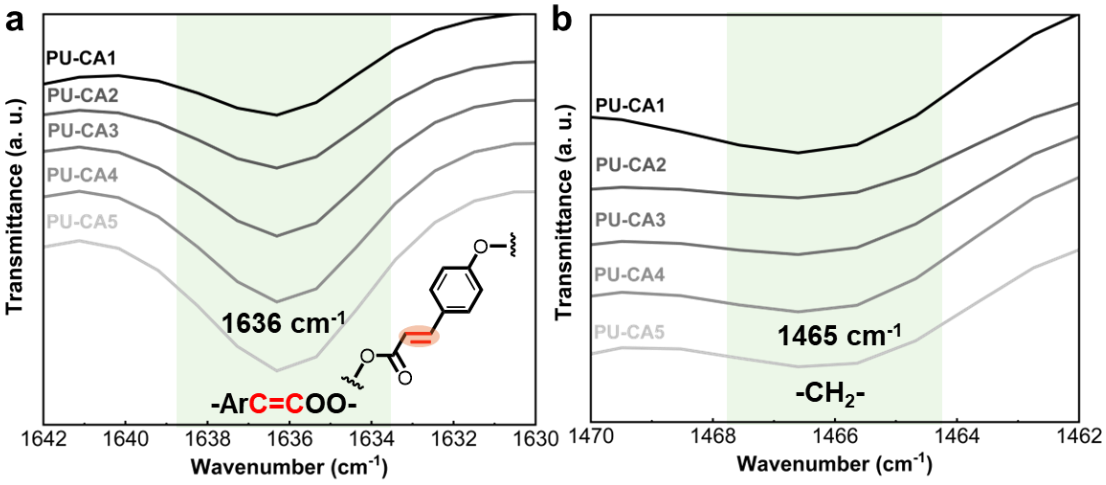

Supplementary Figure 39. Partial FTIR spectra of PU-CA1-5 at 1636 cm^{-1} a) and 1465 cm^{-1} b).

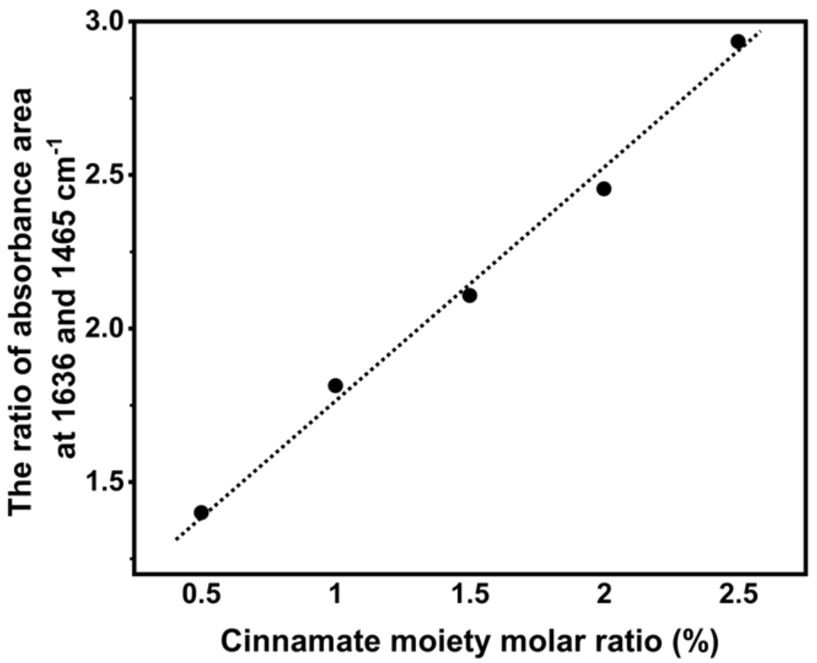

Supplementary Figure 40. Plot of the absorbance area ratio at 1636 and 1465 cm^{-1} versus cinnamate moiety molar ratio for PU-CA1-5.

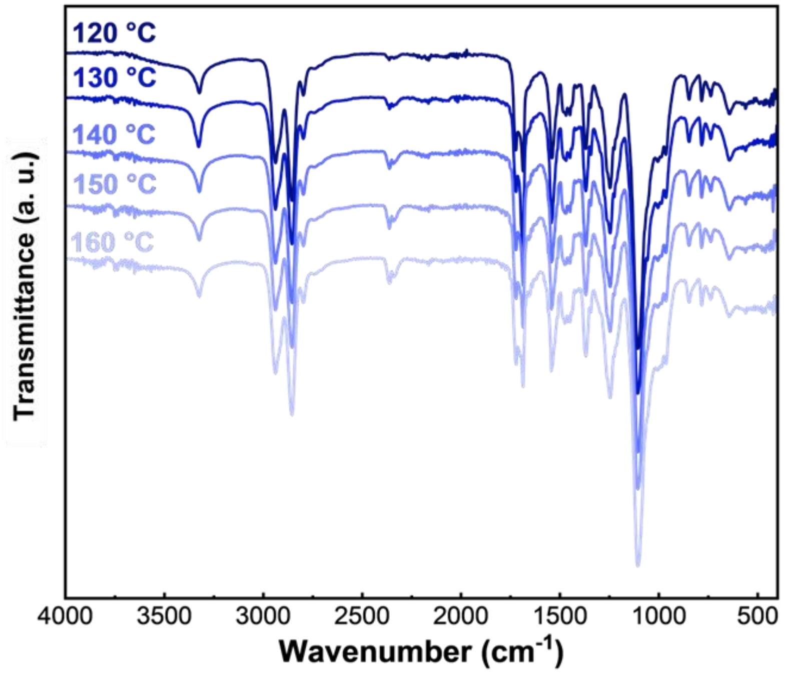

Supplementary Figure 41. FTIR spectra of PU-RM2 after DMA measurements conducted at 120, 130, 140, 150, and 160 °C.

To quantify the extent of mechanophore cleavage at different DMA temperatures, the rupture ratio was calculated by comparing the FTIR absorption area ratio $A_{r(DMA)}$ with that of the reference sample $A_{r(PU\text{-}CA5)}$. PU-CA5 was selected as the reference because its structure already contains the double bond generated after mechanophore scission. Therefore, PU-CA5 represents the fully cleaved state of the mechanosensitive group. The absorption area ratio of $A_{r(PU\text{-}CA5)}$, denoted as, was therefore set to 100% fracture.

The rupture ratio of the truxillate mechanophore in PU-RM2 at different DMA temperatures were calculated according to the following equation.

$$Rupture\ ratio\ (R) = \frac{A_{r(DMA)}}{A_{r(PU-CA5)}} \times 100\%$$

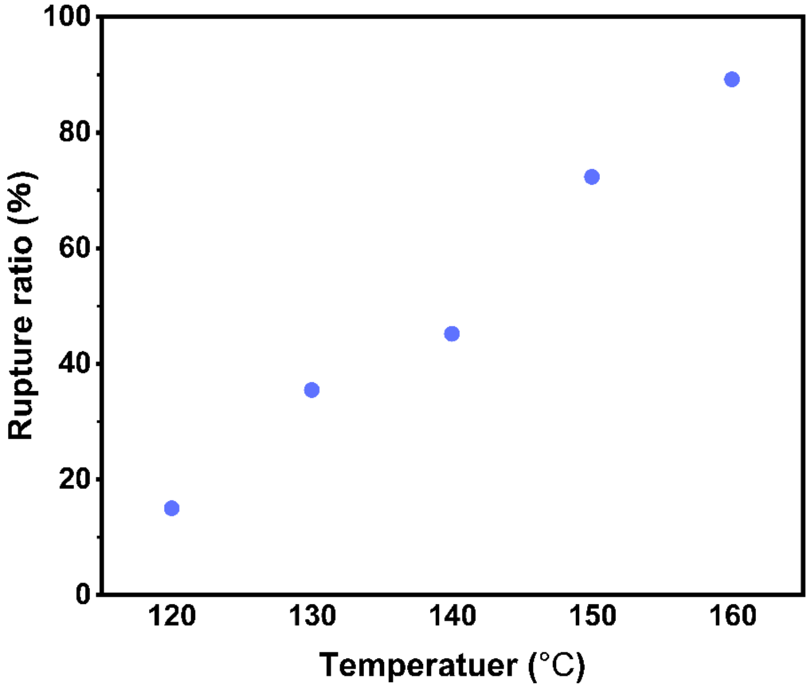

Supplementary Figure 42. Plot of the rupture ratio of PU-RM versus DMA temperature.

7. References

1. Zhang, H.; Li, X.; Lin, Y.; Gao, F.; Tang, Z.; Su, P.; Zhang, W.; Xu, Y.; Weng, W.; Boulatov, R. Multi-modal mechanophores based on cinnamate dimers. *Nat. Commun.* **2017**, *8*, 1147.
2. Bucher, J.; Wurm, T.; Nalivela, K. S.; Rudolph, M.; Rominger, F.; Hashmi, A. S. K. Cyclization of Gold Acetylides: Synthesis of Vinyl Sulfonates via Gold Vinylidene Complexes. *Angew. Chem., Int. Ed.* **2014**, *53*, 3854– 3858.
3. McLaughlin, M. F.; Massolo, E.; Cope, T. A.; Johnson, J. S. Phenolic Oxidation Using H_2O_2 via in Situ Generated para-Quinone Methides for the Preparation of para-Spiroepoxydienones. *Org. Lett.* **2019**, *21*, 6504– 6507.
4. Li, G.; Zhao, J.; Zhang, Z.; Zhao, X.; Cheng, L.; Liu, Y.; Guo, Z.; Yu, W.; Yan, X. Robust and Dynamic Polymer Networks Enabled by Woven Crosslinks. *Angew. Chem.* **2022**, *134* , e202210078.
5. Cantrill, S. J.; Youn, G. J.; Stoddart, J. F.; Williams, D. J. Supramolecular Daisy Chains. *J. Org. Chem.* **2001**, *66*, 6857– 6872.
6. Elizarov, A.; Chang, T.; Chiu, S.-H.; Stoddart, J. F. Self-assembly of dendrimers by slippage. *Org. Lett.* **2002**, *4*, 3565.
7. Gibson, H. W.; Wang, H.; Slebodnick, C.; Merola, J.; Kassel, W. S.; Rheingold, A. L. Isomeric 2, 6-pyridino-cryptands based on dibenzo-24-crown-8. *J. Org. Chem.* **2007**, *72*, 3381.
8. Bellamy, M. K. Using FTIR-ATR Spectroscopy to Teach the Internal Standard Method. *J. Chem. Educ.* **2010**, *87*, 1399.
9. Herrera-González, A. M.; Caldera-Villalobos, M.; Pérez-Mondragón, A. A.; Cuevas-Suárez, C. E.; González-López, J. A. Analysis of Double Bond Conversion of

Photopolymerizable Monomers by FTIR-ATR Spectroscopy. *J. Chem. Educ*. **2019**, *96* , 1786– 1789.
