# Supplementary material for: Toughening elastomer via sequentially activated multi-pathway energy dissipation
Source: Nat Commun. 2026 Jul 1;17:5452. doi: 10.1038/s41467-026-74148-z (PMC13323342; doi:10.1038/s41467-026-74148-z)
Supplement: Supplementary file 2 — Transparent Peer Review file [file 41467_2026_74148_MOESM2_ESM.pdf]

nature portfolio

Peer Review File

Toughening Elastomer via Sequentially Activated Multi-Pathway Energy Dissipation

Corresponding Author: Dr Chunlin Xiao

This file contains all reviewer reports in order by version, followed by all author rebuttals in order by version.

Version 0:

Reviewer comments:

Reviewer #1

(Remarks to the Author)
General assessment: The manuscript reports a mechanically interlocked network (MIN) incorporating a cyclobutane mechanophore within a rotaxane ring to enable a "sequentially activated multi-pathway energy dissipation." While the empirical findings are interesting, the work as presented fails to demonstrate a generalizable "strategy" as the authors claim. Instead, it offers a single, synthetically non-trivial implementation of a well-established approach where the specific benefit of the mechanochemical component is not adequately isolated from the effect of the slidable architecture itself.

Major critiques
1. Lack of conceptual novelty and proper contextualization: The "multi-stage" dissipation motif—including multiple sequential sacrificial bonds, a combinations of sacrificial bonds with rotaxane-mediated sliding—is well-established. Specifically, cyclobutane-based cinnamic acid derivatives have a long history in polymer mechanochemistry due to their favorable and tunable kinetics, which the authors fail to cite. While this specific combination of sliding (MIN) and dissociation (mechanophore) appears to be new, it does not represent a conceptually new design paradigm. The third mechanism claimed by the authors ("formation of woven networks") remains puzzling, as dissociation of cyclobutane does not increase the "woven" nature of the network beyond the pre-existing topology.
2. Deficiencies in control experiments The central claim of the paper rests on the synergy between the MIN and the mechanochemical moiety. However, the control experiments are insufficient to justify this:
• The "mechanochemical value" gap: The authors compare a mechanochemically active MIN to non-interlocked controls. To isolate the benefit of the cyclobutane dissociation, a mechanochemically inert MIN control is essential.
• Marginal gains: Without this control, it is impossible to determine how much statistically significant dissipative benefit the mechanophore adds. In other materials, rotaxane-mediated backbone sliding dominates incremental toughness. The fact that changing the fraction of the rotoxane unit by just 1% eliminates the increase toughness further weakens the claim of any purported synergy and must certainly be placed in context of structure/property relationship in other MINs.
3. Unsupported mechanistic claims: I find the "templated-polymerization" and "woven network" narrative technically unsupported. Since cyclobutane dissociation cannot reduce the average segmental length or introduce new topological entanglements not already present in the MIN precursor (only change their nature), the claim that the network becomes "more woven" post-dissociation lacks a physical basis. The authors provide no evidence that converting a mechanical crosslink to a topological one toughens material more than comparable hidden-length release does in non-interlocked systems.
4. Utility vs. synthetic complexity The potential impact of this work is hampered by the significant synthetic hurdle of incorporating mechanophores into polyrotaxane rings. For such complexity to be justified, the paper must demonstrate either a new conceptual or design paradigm or a commensurate improvement in mechanical properties. Currently, the "oversold" novelty and limited magnitude of improvement make it difficult to see how this approach would be adopted by researchers in the field.

Summary: In its current form, the manuscript lacks the comparative rigor—specifically the MIN inert control—necessary to support its primary claims. The empirical results are noteworthy but are presented without the context or the control data required to demonstrate a functional "strategy" for material design.

Reviewer #2

(Remarks to the Author)
The authors have reported a work on toughening polymers using a combined energy dissipation mechanisms. The work is interesting and the main arguments are largely supported by the experiment results. It's publishable after some minor corrections which are suggested as follows:

1) it is not mentioned why RM2 is the best in terms of the toughening as compared to RM1 and RM3. could the authors explained this?
2) the schematic shown in Fig. 1 (b) seems not necessary and can be deleted or merged with Fig. 1(c)
3) the dynamic test is quite critical to understand the effects of rotaxane crosslinks. The testing results should be presented in the text rather than in the supplementary.

Reviewer #3

(Remarks to the Author)
In the manuscript titled "Toughening Elastomer via Sequentially Activated Multi-Pathway Energy Dissipation", Li and his collaborators prepared an elastomer with a tertiary energy dissipation pathway by introducing cyclopentadiene crosslinkers featuring a truxinate structure into the polyurethane network. This strategy establishes a novel multi-path energy dissipation coupling mechanism, which effectively enhances the material's toughness. The manuscript demonstrates excellent molecular-level design and provides effective characterization to elucidate the energy dissipation mechanism. Therefore, it is recommended that this manuscript be published after the following revisions:
1. In the introduction section, lines 63-67 state that "most studies have focused on a single energy dissipation mechanism". However, systems such as dual-network hydrogels and sacrificial bond network have already established mature multi-dissipation strategies. The scope of innovation in this paper should be more precisely defined.
2. Line 136 states that "PU-RM2 and PU-C exhibit similar original network density", yet further data on effective crosslink density, obtained from swelling experiments or shear modulus measurements, is required for a more accurate assessment.
3. In the tensile test illustrated in Figure 3, it is recommended that a minimum of three specimens are tested, with error bars to be included in the analysis of Figures 3c and 3e to facilitate a more objective evaluation of the material's mechanical properties.
4. As demonstrated in Figure 4f and 4g, the stress-strain curves following a 10-hour recovery period indicate that the stress recovers to its original strength. However, why does the yield point not recover to its initial level? Could it be that irreversible structural changes have actually occurred within the polymer?
5. At different strain rates, polymers exhibit varying energy dissipation patterns as the strain rate increases. At higher strain rates, energy dissipation begins to decrease again. What might be the reason for this, and how might it relate to changes in the polymer's internal structure?
6. Based on the DMA results in Figure 5b, the failure of covalent crosslinks in PU-C appears to precede the rupture of the truxinate structure. Why is this the case?
7. I note that "woven network" is sometimes singular and sometimes plural; it is recommended to standardize the form.

Version 1:

Reviewer comments:

Reviewer #1

(Remarks to the Author)
The authors addressed two of my concerns fully, one partially, and left the remaining major issues unresolved. Accordingly, I am unable to support acceptance of the current draft.

Issues addressed
1. The authors prepared the previously missing control and report its critical mechanical properties. The molecular and physical interpretation of the observed trend is not obvious and appears to contradict findings from a recent paper by Otsuka et al., while being seemingly consistent with broader themes previously outlined by Craig, Creton, and others: namely, that critical mechanical properties are often considerably more sensitive to covalent mechanochemical processes than to purely physical dissipative mechanisms. None of this relevant context is cited or discussed.
2. The authors removed the speculative claims regarding templated polymerization.
3. The authors added several citations on the mechanochemistry of cyclobutane cores. However, citation is not the same as contextualization. The manuscript remains silent on how the authors' use of cyclobutane derivatives is informed by this prior work or how it advances the practical or conceptual exploitability of cyclobutane mechanochemistry.

Remaining concerns
1. The authors continue to claim that "three distinct types of energy dissipation pathways coexist" in their materials without providing convincing evidence for the third mechanism.
2. The manuscript repeatedly refers to the "woven" nature of the network without defining the term, explaining how this "wovenness" was quantified, or clarifying how such a structure forms and dissipates local stress. Statements such as "MINs gradually convert into the woven networks along with the rupture of rotaxane crosslinkers, preserving the network integrity and serving as the tertiary energy dissipation pathway" simply restate the claim without providing clarity or supporting evidence.

3. The authors continue to avoid even qualitative comparison with previously reported materials that incorporate multiple dissipative mechanisms, while presenting their work as a paradigm-shifting strategy rather than another implementation of a well-established idea.
4. The authors' rebuttal to my objection and that of Reviewer 3 regarding the overclaimed novelty is internally inconsistent. For example, they justify the significance of their work by stating that "the toughening mechanisms when multiple (three) distinct types of energy dissipation pathways coexist remain inadequately elucidated." The authors report another example of a material containing competing dissipative mechanisms, but do not offering new insights—at least none that I can infer—into the generalizable molecular requirements for multi-dissipative designs, nor into the molecular or physical mechanisms that control the resulting changes in bulk mechanical properties.

I do not expect the authors to answer every plausible question. Good research often raises more questions than it resolves. However, the manuscript should acknowledge the conceptual, mechanistic, and quantitative uncertainties and ambiguities, avoid overstating its novelty, and place its results faithfully within the existing literature.

Reviewer #2

(Remarks to the Author)
I have gone through the revised manuscript and the response to the comments from all three reviewers. I think they have fully addressed all reviewers' comments and added or modified the draft accordingly. Therefore, I would like to recommend this to be considered for publication in NC. Thanks.

Reviewer #3

(Remarks to the Author)
The raised concerns have been well addressed. Please consider to accept the revised manuscript as is.

Version 2:

Reviewer comments:

Reviewer #1

(Remarks to the Author)
The revisions didn't materially change my assessment of the paper. I find the science largely credible and the small tweaks in the presentation that the authors made are not substantive enough to alleviate my concerns summarized in hte closing paragraph of my previous review. The authors and I seem to be talking a bit past each other. Given that the most likely impact of the contentious aspects is on how experts in the field perceive authors' credibility and intellectual rigor, the supportive stances of the other reviewers and the low likelihood that an additional round of review will narrow the remaining gap in our mutual understanding of the appropriate contextualization of work, I consider proceeding with accepting the paper as the most sensible course of action.

Open Access This Peer Review File is licensed under a Creative Commons Attribution 4.0 International License, which permits use, sharing, adaptation, distribution and reproduction in any medium or format, as long as you give appropriate credit to the original author(s) and the source, provide a link to the Creative Commons license, and indicate if changes were made.
In cases where reviewers are anonymous, credit should be given to 'Anonymous Referee' and the source.
The images or other third party material in this Peer Review File are included in the article’s Creative Commons license, unless indicated otherwise in a credit line to the material. If material is not included in the article’s Creative Commons license and your intended use is not permitted by statutory regulation or exceeds the permitted use, you will need to obtain permission directly from the copyright holder.
To view a copy of this license, visit https://creativecommons.org/licenses/by/4.0/

Response to Reviewer 1’s Comments

Comment 1 (General assessment).

The manuscript reports a mechanically interlocked network (MIN) incorporating a cyclobutane mechanophore within a rotaxane ring to enable a "sequentially activated multi-pathway energy dissipation." While the empirical findings are interesting, the work as presented fails to demonstrate a generalizable "strategy” as the authors claim. Instead, it offers a single, synthetically non-trivial implementation of a well-established approach where the specific benefit of the mechanochemical component is not adequately isolated from the effect of the slidable architecture itself.

Response 1.

We appreciate the reviewers' constructive comments. To clarify the discussion on multi-path energy dissipation, which is gradually becoming more active, we newly prepared an appropriate control sample “**PU-R**” (see figure below) that includes MINs without mechanical receptors and compared the data. Analysis of the mechanical properties and DMA results of all reference materials alongside this sample provided further evidence supporting the proposed mechanism.

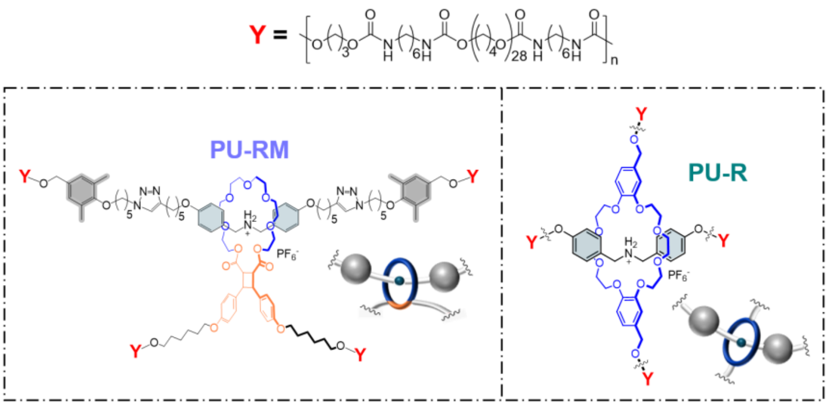

Comment 2 (Major critiques-1).

Lack of conceptual novelty and proper contextualization: The "multi-stage" dissipation motif—including multiple sequential sacrificial bonds, a combination of sacrificial bonds with rotaxane-mediated sliding—is well-established. Specifically, cyclobutane-based cinnamic acid derivatives have a long history in polymer mechanochemistry due to their favorable and tunable kinetics, which the authors fail to cite. While this specific combination of sliding (MIN) and dissociation (mechanophore) appears to be new, it does not represent a conceptually new design paradigm. The third mechanism claimed by the authors ("formation of woven networks") remains puzzling, as dissociation of cyclobutane does not increase the "woven" nature of the network beyond the pre-existing topology.

Response 2.

As you pointed out, multiple dissipation motifs incorporated into mechanically interlocked molecules have been reported to date. However, attempts to integrate multiple pathways—including mechanically interlocked networks (MINs), mechanophores, and woven networks—remain scarce. Consequently, the toughening mechanism when multiple (three) distinct types of energy dissipation pathways coexist remains poorly understood. Therefore, we have revised our claim regarding novelty as follows.

Moreover, efforts to integrate multiple pathways, including MINs, mechanophores, and woven networks, remain scarce, and the toughening mechanisms when multiple (three) distinct types of energy dissipation pathways coexist remain inadequately elucidated.

Previous studies on mechanically interlinked polymers (MIPs) have reported combining cleavable mechanical receptors with rotaxane structures, enabling visualization of rotaxane slippage under mechanical force (e.g., research by the Otsuka and Takada groups: *Chem. Commun.* **2020**, *56*, 3361). Additionally, molecular-level applications such as controlled molecular release and mechanochemical reactions have been reported (e.g., De Bo group research: *J. Am. Chem. Soc.* **2024**, *146*, 16381; *Angew. Chem. Int. Ed.* **2025**, *64*, e202511039; *Nature* **2024**, *628*, 320). Reports integrating the sacrificial bond properties of cleavable mechanophores with dynamically responsive structures mechanically coupled for energy dissipation were published in 2023 by Otsuka *et al.* (*J. Am. Chem. Soc.* **2023**, *145*, 23794). However, the molecular design in this work fundamentally differs from ours. In their design, the mechanophore functions as a bridge connecting two rotaxane units. In contrast, our design embeds the mechanophore within the macrocyclic skeleton of the rotaxane. Furthermore, the primary focus of Otsuka et al.'s work was to demonstrate the sacrificial bond effect of rotaxane structures within a polymer network, not to investigate how a cleavable mechanophore interacts with a mechanically coupled structure. As a result, the detailed mechanical properties and rheological characteristics of such materials have not been systematically investigated. In contrast, our study is the first to investigate the relationship between structural changes under mechanical stress and mechanical performance through a synergistic analysis of mechanical testing and spectroscopic characterization. This study elucidated the mechanism whereby force-induced cleavage of the truxinate units and the sliding motion of the rotaxane itself interact to comprehensively influence the mechanical properties.

In the previous manuscript, key references regarding the application of cyclobutane-based cinnamic acid derivatives in polymers were missing. The following representative studies from the past 20 years have been appropriately cited in the revised manuscript.

[41] Chung, C. M., Roh, Y. S., Cho, S. Y. & Kim, J. G. Crack healing in polymeric materials via photochemical [2+2] cycloaddition. *Chem. Mater.* **16**, 3982–3984 (2004).

[42]Cho, S. Y., Kim, J. G. & Chung, C. M. A fluorescent crack sensor based on cyclobutane-containing crosslinked polymers of tricinnamates. *Sens. Actuators B Chem.* **134**, 822–825 (2008).

[48]Pill, M. F., Holz, K., Preußke, N., Berger, F., Clausen-Schaumann, H., Lüning, U. & Beyer, M. K. Mechanochemical cycloreversion of cyclobutane observed at the single molecule level. *Chem. Eur. J.* **22**, 12034–12039 (2016).

[21]Tian, Y., Cao, X., Li, X., Zhang, H., Sun, C. L., Xu, Y. & Boulatov, R. A polymer with mechanochemically active hidden length. *J. Am. Chem. Soc.* **142**, 18687–18697 (2020).

[25]Bowser, B. H., Wang, S., Kouznetsova, T. B., Beech, H. K., Olsen, B. D., Rubinstein, M. & Craig, S. L. Single-event spectroscopy and unravelling kinetics of covalent domains based on cyclobutane mechanophores. *J. Am. Chem. Soc.* **143**, 5269–5276 (2021).

[20]Wang, S., Beech, H. K., Bowser, B. H., Kouznetsova, T. B., Olsen, B. D., Rubinstein, M. & Craig, S. L. Mechanism dictates mechanics: a molecular substituent effect in the macroscopic fracture of a covalent polymer network. *J. Am. Chem. Soc.* **143**, 3714–3718 (2021).

This research primarily focuses on establishing and elucidating the “sliding-dissociation (-incorporation)” mechanism in polymer design, and demonstrating its potential as a design paradigm is essential. By utilizing the rotaxane-type mechanophore framework we developed as a foundation, both structural and functional diversity can be readily achieved by modifying the polymer backbone and mechanophore components (as shown in the schematic diagram below). We are confident this mechanism can be extended to a wide range of polymer systems.

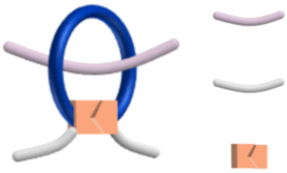

As the reviewer pointed out, we agree that cyclobutane dissociation does not increase the degree of “interwoven/entangled” network. However, the interwoven topology originates from the mutually entangled structure, and the interwoven structure “converted” from the rotaxane can “inherit” the energy dissipation after rotaxane cleavage. Specifically, as shown in the figure below, the rotaxane-type mechanophore crosslinker (**RM**) dissociates by generating two entangled chains while maintaining the original topological constraints. In contrast, the breakage of covalent crosslinks produces free chains with no residual topological constraints. In other words, covalent crosslinking polymers like **PU-C** lack structural elements designed to “take over” further energy dissipation after their chemical structure is destroyed. Conversely, in **PU-RM**, the network behaves as a MIN prior to cyclobutane cleavage. After rotaxane cleavage, the network gradually “transforms” into a woven structure, which then takes over energy dissipation. This mechanism is supported by the comparison of DMA results for **PU-R** and **PU-RM** in the revised manuscript. Similar designs incorporating such topological constraints in polymers have been reported in recent years. (*J. Am. Chem. Soc.* **2025**, *147*, 8695; *Angew. Chem. Int. Ed.* **2022**, *61*, e202210078; *Angew. Chem. Int. Ed.* **2025**, *64*, e202506559; *Nat. Mater.* **2026**, *25*, 107). To avoid potential misunderstanding, we have revised the related description such as:

Therefore, MINs gradually convert into the woven networks along with the rupture of rotaxane crosslinkers, preserving the network integrity and serving as the tertiary energy dissipation pathway (Page 4).

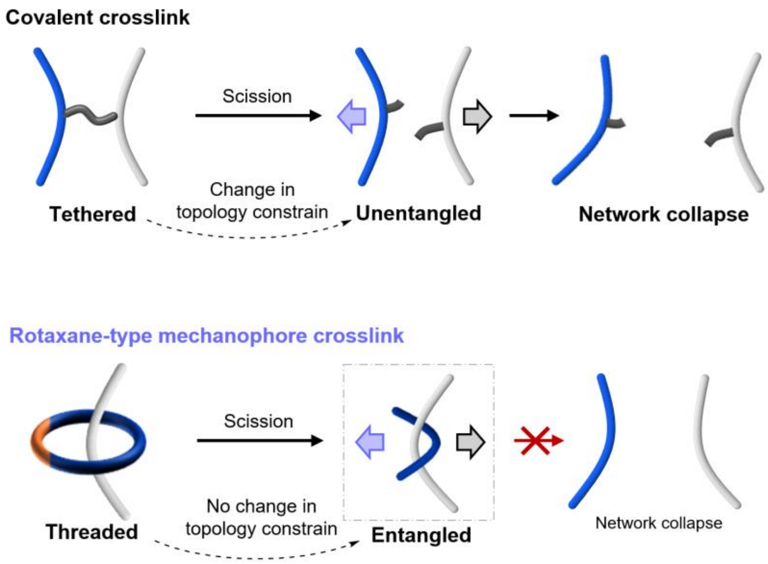

Comment 3 (Major critiques-2).

Deficiencies in control experiments: The central claim of the paper rests on the synergy between the MIN and the mechanochemical moiety. However, the control experiments are insufficient to justify this:

• The "mechanochemical value" gap: The authors compare a mechanochemically active MIN to non-interlocked controls. To isolate the benefit of the cyclobutane dissociation, a mechanochemically inert MIN control is essential.

• Marginal gains: Without this control, it is impossible to determine how much statistically significant dissipative benefit the mechanophore adds. In other materials, rotaxane-mediated backbone sliding dominates incremental toughness. The fact that changing the fraction of the rotoxane unit by just 1% eliminates the increase toughness further weakens the claim of any purported synergy and must certainly be placed in context of structure/property relationship in other MINs.

Response 3.

We thank the reviewer for constructive suggestions. We have added the data for an appropriate control sample. We have prepared the mechanochemically inert MIN control (**PU-R**) and compared it to mechanochemically active MIN (**PU-RM2**), as suggested by the reviewer. The toughness of **PU-R** (123 ± 9) increases to 200 ± 4 upon incorporation of the truxinate mechanophore (**PU-RM2**), corresponding to a +77 absolute increase and a 63% relative enhancement. Notably, all the differences substantially exceed the statistical variations of all samples. Crosslinked **PU-R** shows higher strength but lower elongation than linear **PU-MCM**, which is reasonable given the network structure in **PU-R**. Although the mobility of rotaxane can improve deformability compared to purely covalent networks, the present results suggest that sliding alone is insufficient to recover the elongation level of the linear sample capable of releasing the hidden length. **PU-RM2** exhibits both enhanced strength and elongation relative to controls. Such behavior indicates a cooperative effect between rotaxane-mediated sliding and mechanophore scission. Based on the new data, we have revised the discussion part in manuscript accordingly (Page 9).

	Stress (MPa)	Strain (%)	Toughness (MJ/m^3)
**PU-MCM**	30 ± 2	920 ± 31	140 ± 7
**PU-R**	40 ± 3	791 ± 45	123 ± 9
**PU-RM2**	53 ± 2	967 ± 24	200 ± 4

Comment 4 (Major critiques-3).

Unsupported mechanistic claims: I find the "templated-polymerization" and "woven network" narrative technically unsupported. Since cyclobutane dissociation cannot reduce the average segmental length or introduce new topological entanglements not already present in the MIN

precursor (only change their nature), the claim that the network becomes "more woven" post-dissociation lacks a physical basis. The authors provide no evidence that converting a mechanical crosslink to a topological one toughens material more than comparable hidden-length release does in non-interlocked systems.

Response 4.

We have removed the ambiguous term "templated-polymerization" from the revised manuscript to avoid potential misunderstanding.

As previously indicated in Response 2, we did not intend to claim that mechanophore activation increases the woven "degree" of the polymer network. Instead, the woven/entangled chains generated upon cyclobutane dissociation take over energy dissipation after rotaxane crosslinking and hidden-length release, thereby maintaining, rather than enhancing, the topological constraint and network integrity. The extent to which the woven structure directly contributes to tensile properties has not been independently quantified, because characterizing this woven structure in situ during mechanical testing (such as tensile testing) is difficult. However, dynamic mechanical analysis (DMA) comparing three crosslinked samples (**PU-C**, **PU-R**, and **PU-RM2**) provided evidence of additional structural contribution after rotaxane dissociation. The viscoelastic response of **PU-RM2** (directly related to mechanical performance) was markedly different from the control samples, suggesting a contribution from the woven structure in this design (see Revised Figure 5 and the corresponding discussion in the text for details).

Comment 5 (Major critiques-4).

Utility vs. synthetic complexity. The potential impact of this work is hampered by the significant synthetic hurdle of incorporating mechanophores into polyrotaxane rings. For such complexity to be justified, the paper must demonstrate either a new conceptual or design paradigm or a commensurate improvement in mechanical properties. Currently, the "oversold" novelty and limited magnitude of improvement make it difficult to see how this approach would be adopted by researchers in the field.

Response 5.

Thank you for your concern regarding the practicality of our research. In rotaxane synthesis, the macrocyclization reaction for constructing the host and the final rotaxanization reaction are typically the most challenging steps with the lowest yields. In our design, both **R** and **RM** were prepared using the same rotaxanization method. The main difference lies in the preparation of the corresponding macrocyclic compounds (DB24C derivatives versus **MCM**). The macrocyclic compound **MCM**, which incorporates the mechanophore, was obtained in nearly quantitative yield (with minor losses during chromatography purification) via a one-step photochemical reaction. In contrast, the conventional crown ether host **19** is synthesized via a conventional Williamson etherification reaction in a yield of about 40%. Therefore, incorporating the mechanophore into the macroring compound does not add complexity to the synthesis compared to the standard crown ether system.

Cyclization of **19** by Williamson etherification (Yield: 40% over two steps)

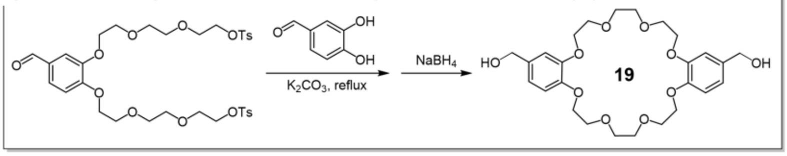

Cyclization of **MCM** by [2+2]photocycloaddition (Yield: 95%)

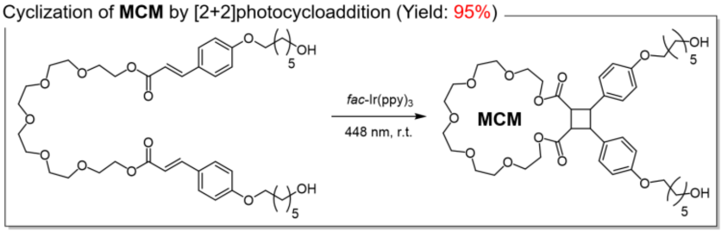

Our strategy achieves a significant improvement in toughness even compared to MINs-containing **PU-R**, one of the most effective toughness enhancement methods reported to date. The convenience of modifying functional groups other than -OH also gives this design potential as a versatile scaffold.

Comment 6 (Summary in Reviewer 1's comments).

Summary: In its current form, the manuscript lacks the comparative rigor—specifically the MIN inert control—necessary to support its primary claims. The empirical results are noteworthy but are presented without the context or the control data required to demonstrate a functional "strategy" for material design.

Response 6.

We thank the reviewer for the rigorous and constructive comments, which have helped improve the quality of the manuscript. We have added appropriate control experiments to further substantiate our claims.

Response to Reviewer 2's Comments

Comment 1 (Remarks to the Author).

The authors have reported a work on toughening polymers using a combined energy dissipation mechanism. The work is interesting and the main arguments are largely supported by the experiment results. It's publishable after some minor corrections which are suggested as follows:

Response 1.

We thank the reviewer for the interest and positive comments on our work. We have addressed the comments raised and revised the manuscript accordingly.

Comment 2 (#1 in Reviewer 2's comments).

(1) It is not mentioned why RM2 is the best in terms of the toughening as compared to **RM1** and **RM3**. Could the authors explain this?

Response 2.

We appreciate the reviewer's concern. The mechanical properties of **PU-RMn** depend on the interaction of three factors. Therefore, at low crosslinking density (**RM1**), the network is relatively loose and stress transfer is inefficient. Consequently, the contributions from rotaxane sliding and mechanophore-related dissipation are not fully exploited, leading to the lowest toughness. At the highest crosslink density (**RM3**), the network is strengthened and stress transfer is improved, leading to increased energy dissipation and higher toughness than **RM1**. However, increasing crosslink density restricts chain mobility and reduces extensibility, limiting the full effects of rotaxane sliding and mechanophore activation. Consequently, while **RM3**'s toughness exceeds that of the 1% sample, it is thought not to surpass that of **RM2**.

Comment 3 (#2 in Reviewer 2's comments).

The schematic shown in Fig. 1 (b) seems not necessary and can be deleted or merged with Fig. 1(c).

Response 3.

We thank the reviewer for the instructive suggestion. We have simplified Fig. 1 according to the reviewer's suggestion as shown below.

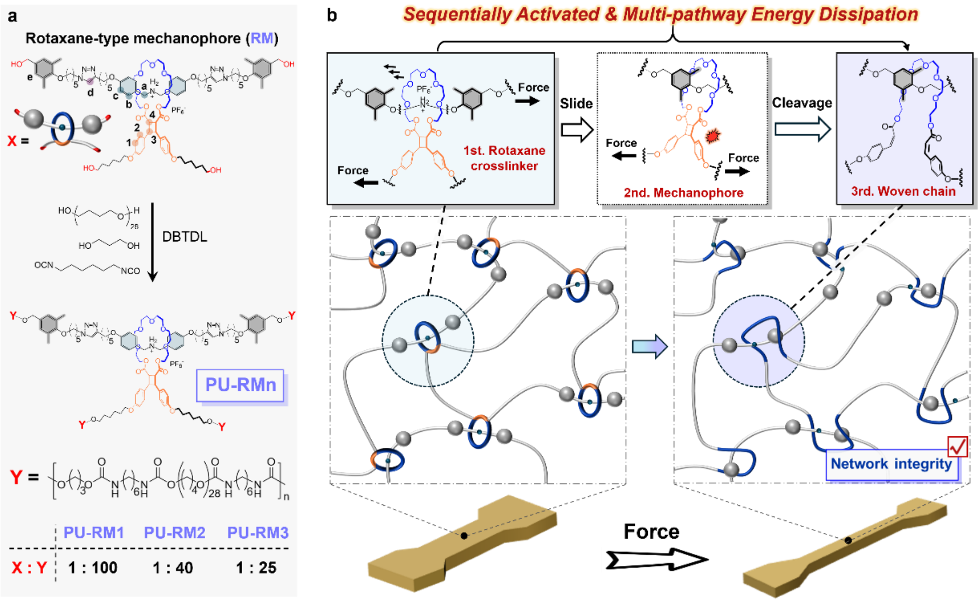

Comment 4 (#3 in Reviewer 2's comments).

The dynamic test is quite critical to understand the effects of rotaxane crosslinks. The testing results should be presented in the text rather than in the supplementary.

Response 4.

We thank the reviewer for the instructive suggestion. We have put the DMA result of **PU-RM2** to the main text, with comparison to the covalently crosslinked sample **PU-C** and mechanophore-free rotaxane-crosslinked sample **PU-R**.

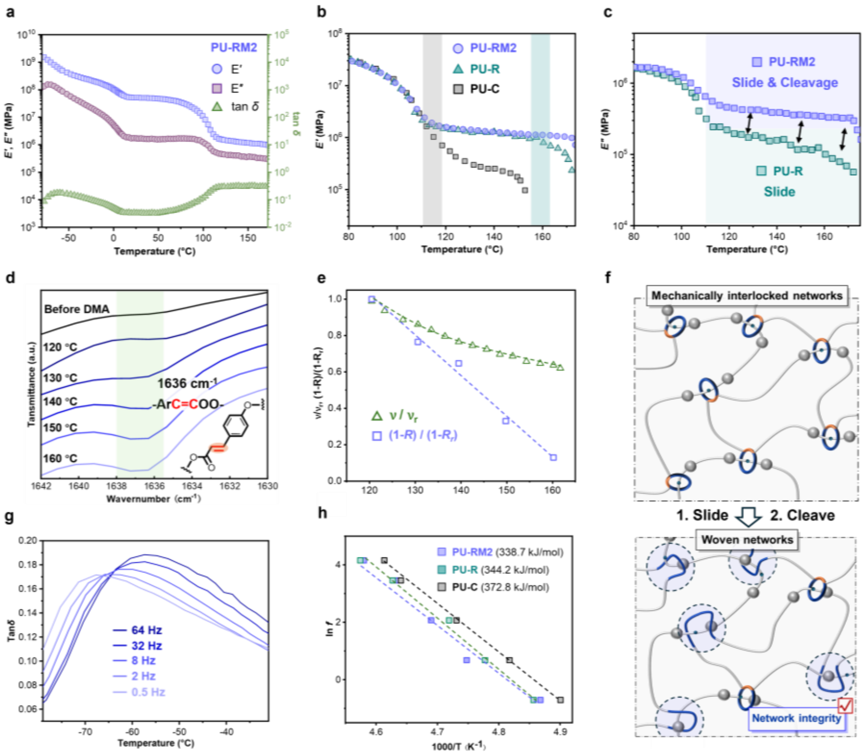

Figure 5. Preservation of network integrity by inherently formed woven networks. (a) Dynamic mechanical analysis of **PU-RM2** by temperature sweep. (b) Storage modulus (E') curves of **PU-RM2**, **PU-R**, and **PU-C** in the temperature range from 80 °C to 170 °C. (c) Loss modulus (E'') curves of **PU-RM2** and **PU-R** in the temperature range from 80 °C to 170 °C. (d) Partial FTIR spectra of **PU-RM2** measured at different temperature after DMA (120, 130, 140, 150, and 160 °C, respectively). (e) Plot of v/v_r or $(1\text{-}R)/(1\text{-}R_r)$ versus DMA temperature, where v refers to the network

density, v_r refers to network density at 120 °C in DMA, R refers to rupture ratio of cyclobutane (see Figures S31-35), R_r refers to rupture ratio of cyclobutane at 120 °C in DMA. (f) Schematic presentation of formation of woven networks along with the rupture of rotaxane crosslink. (g) Temperature sweeps of **PU-RM2** under different frequencies using DMA. The transition occurs at approximately -80 to -30 °C. (h) Arrhenius plots of transitions for **PU-RM2**, **PU-R** and **PU-C** based on the frequency-dependent shifts of the tan δ peaks, respectively.

The moduli of the rotaxane-crosslinked **PU-R** and **PU-RM2** enter an elastic plateau region, where the decrease becomes much more gradual as the temperature increases to 110 °C. In this region, the E′ values of **PU-R** and **PU-RM2** are comparable and remain higher than that of **PU-C**. This result indicates that the mobility of the rotaxane crosslinking nodes effectively dissipates energy, thereby preventing premature rupture in chemical structures. In addition, the decrease in E' at around 160 °C indicates the loss of network stability in **PU-R** due to the limited energy dissipation capacity of the MINs at such condition. In contrast, **PU-RM2** shows no obvious loss of network elasticity until 175 °C (Fig. 5b). The loss modulus E'' of **PU-RM2** is remarkably higher than that of **PU-R** between 110 °C and 175 °C (Fig. 5c). These results suggest that although both MIN-containing samples possess comparable network elasticity, **PU-RM2** exhibits more effective energy dissipation within the elastic plateau region. This enhanced dissipation and network stability may arise from the sacrificial mechanophore cleavage and the subsequently formed woven networks, as supported by FTIR measurements.

Response to Reviewer 3’s Comments

Comment 1 (Remarks to the Author).

In the manuscript titled “Toughening Elastomer via Sequentially Activated Multi-Pathway Energy Dissipation”, Li and his collaborators prepared an elastomer with a tertiary energy dissipation pathway by introducing cyclopentadiene crosslinkers featuring a truxinate structure into the polyurethane network. This strategy establishes a novel multi-path energy dissipation coupling mechanism, which effectively enhances the material’s toughness. The manuscript demonstrates excellent molecular-level design and provides effective characterization to elucidate the energy dissipation mechanism. Therefore, it is recommended that this manuscript be published after the following revisions:

Response 1.

We thank the reviewer for the positive comments. We have revised the manuscript accordingly.

Comment 2 (#1 in Reviewer 3’s comments).

In the introduction section, lines 63-67 state that “most studies have focused on a single energy dissipation mechanism”. However, systems such as dual-network hydrogels and sacrificial bond network have already established mature multi-dissipation strategies. The scope of innovation in this paper should be more precisely defined.

Response 2.

We thank the reviewer for the instructive suggestion. We have revised the sentence to more precisely define the scope of innovation:

Moreover, efforts to integrate multiple pathways, including MINs, mechanophores, and woven networks, remain scarce, and the toughening mechanisms when multiple (three) distinct types of energy dissipation pathways coexist remain inadequately elucidated.

Comment 3 (#2 in Reviewer 3’s comments).

Line 136 states that “PU-RM2 and PU-C exhibit similar original network density”, yet further data on effective crosslink density, obtained from swelling experiments or shear modulus measurements, is required for a more accurate assessment.

Response 3.

We thank the reviewer for the instructive suggestion. We have performed swelling experiments for all crosslinked samples (**PU-C**, **PU-R**, and **PU-RM2**). Results show a similar swelling ratio of three samples, indicating a comparable effective crosslink density among them (Please see Figure S12 in the supplementary).

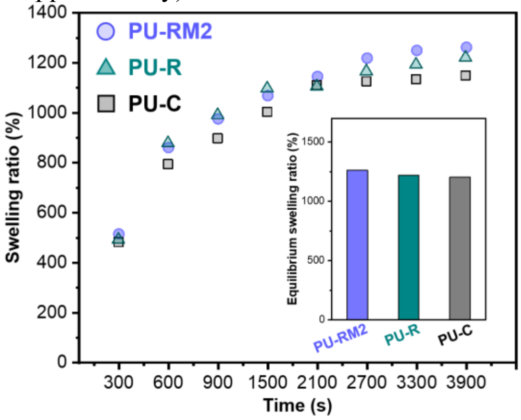

Swelling experiments of three crosslinked polyurethane in THF

Comment 4 (#3 in Reviewer 3's comments).

In the tensile test illustrated in Figure 3, it is recommended that a minimum of three specimens are tested, with error bars to be included in the analysis of Figures 3c and 3e to facilitate a more objective evaluation of the material's mechanical properties.

Response 4.

We thank the reviewer for the instructive suggestion. Figure 3 includes error bars calculated from experimental data using at least three test specimens.

Comment 5 (#4 in Reviewer 3's comments).

As demonstrated in Figure 4f and 4g, the stress-strain curves following a 10-hour recovery period indicate that the stress recovers to its original strength. However, why does the yield point not recover to its initial level? Could it be that irreversible structural changes have actually occurred within the polymer?

Response 5.

We thank the reviewer for this concern. According to the FTIR measurements shown in Figure 4, no cyclobutane cleavage was observed within the spectroscopically detectable range under low load. Therefore, we could exclude the irreversible cleavage of cyclobutane or other stronger covalent bonds under these conditions. On the other hand, the observed behavior does not necessarily indicate irreversible damage to the polymer backbone or permanent failure of the network. Instead, it is more reasonably attributed to microscopic network reorganization induced by deformation: ring sliding of the rotaxane crosslinkers along the polymer chains occurs concurrently with the dissociation of hydrogen networks. Upon unloading and recovery, hydrogen bonds can reform at new sites, leading to a lower yield point. Similar behavior has been reported in other mechanically interlocked network systems (*Adv. Mater.* **2020**, *32*, 2000345).

Comment 6 (#5 in Reviewer 3's comments).

At different strain rates, polymers exhibit varying energy dissipation patterns as the strain rate increases. At higher strain rates, energy dissipation begins to decrease again. What might be the reason for this, and how might it relate to changes in the polymer's internal structure?

Response 6.

We thank the reviewer for pointing this out. Due to the complex interplay among the three energy dissipation pathways, a detailed investigation of the correlation between the internal

structure of the polymer network and its mechanical performance at different strain rates is still ongoing and will be reported in future work. Given that, we have provided a qualitative discussion in the manuscript.

In contrast, the energy dissipation of **PU-RM2** increases as the deformation rate rises from 30 to 200 mm min^{-1}, and then decreases at higher deformation rates (Figs. S26 and S27). This non-monotonic trend originated from the coupling between rotaxane's dynamicity and activation of mechanophore: at low deformation rates, with sufficient relaxation, the rotaxane motifs undergo reversible rearrangements, which allows the rebind of macrocycle to the station on axle. Therefore, the activation of mechanophore was prevented, resulting in limited energy dissipation. As the deformation rate increases to an intermediate regime (200 mm min^{-1}), stress can no longer be fully released through rotaxane rebinding, and thereby effectively drives the sequential activation of rotaxane sliding followed by mechanophore scission, leading to a maximum energy dissipation. At high deformation rates, the rapid loading bypasses rotaxane sliding that should have been the first dissipation pathway, resulting in direct activation of mechanophore or even failure of local segments in a more quasi-brittle manner. This non-monotonic rate dependence highlights a time-scale matching window where the topological energy dissipation architecture of **PU-RM2** is most effectively utilized.

Comment 7 (#6 in Reviewer 3's comments).

Based on the DMA results in Figure 5b, the failure of covalent crosslinks in PU-C appears to precede the rupture of the truxinate structure. Why is this the case?

Response 7.

We thank the reviewer for pointing this out. With supplementing and comparing the DMA result of **PU-R** (new control with mechanophore-free MINs), we would like to explain the earlier decrease in E' of **PU-C** as rupture of shorter PU chains in **PU-C** compared with those in the PU containing MINs, which could be supported by the previous report (*Angew. Chem. Int. Ed.* **2025**, *64*, e202506559). Therefore, we have revised the related discussion in the manuscript:

Within this plateau, E' and E'' gradually decreased with increasing temperature, likely due to the earlier rupture of shorter PU chains in **PU-C** compared with those in the PU containing MINs [28]. In contrast, the moduli of the rotaxane-crosslinked **PU-R** and **PU-RM2** enter an elastic plateau region, where the decrease becomes much more gradual as the temperature increases to 110 °C. In this region, the E′ values of **PU-R** and **PU-RM2** are comparable and remain higher than that of **PU-C**. This result indicates that the mobility of the rotaxane crosslinking nodes effectively dissipates energy, thereby preventing premature rupture in chemical structures.

Comment 8 (#7 in Reviewer 3's comments).

I note that "woven network" is sometimes singular and sometimes plural; it is recommended to standardize the form.

Response 8.

We thank the reviewer for the careful examination of our manuscript. We have carefully checked the text and corrected this and other inaccuracies.

Response to Reviewer 1's Comments

Issues addressed

Comment 1.

The authors prepared the previously missing control and report its critical mechanical properties. The molecular and physical interpretation of the observed trend is not obvious and appears to contradict findings from a recent paper by Otsuka et al., while being seemingly consistent with broader themes previously outlined by Craig, Creton, and others: namely, that critical mechanical properties are often considerably more sensitive to covalent mechanochemical processes than to purely physical dissipative mechanisms. None of this relevant context is cited or discussed.

Response 1.

We note the reviewer's comment on the seemingly opposing trends reported in previous studies. A presentative work by Creton and co-workers has demonstrated that the selective scission of covalent mechanophores can effectively dissipate large amounts of energy (*Phys. Rev. X 10, 041045 (2020)*), which is consistent with our observation that the linear **PU-MCM** exhibits higher toughness than the rotaxane-crosslinked **PU-R**. However, this does not imply that our results contradict the conclusions reported by Otsuka and co-workers (*J. Am. Chem. Soc. 145, 23794–23801 (2023)*), namely, "sacrificial mechanical bonds can be as effective as sacrificial covalent bonds in enhancing the toughness of cross-linked polymers".

In our system, the macrocycle size in the rotaxane crosslinker (**RM**) is smaller than that of DB24C used in Otsuka's work (with identical stopper size), making force-induced dethreading less favorable. We thereby deduce that the mechanical bond in **RM** does not primarily function as a mechanochemical protecting group under force. Instead, rotaxane sliding serves as an energy dissipation mechanism prior to the sacrificial scission of the mechanophore.

Therefore, our results complement, to some extent, a limitation in the system reported by Otsuka and co-workers, where no clear synergistic dissipation effect between the rotaxane and the mechanophore was observed when the mechanophore serves as a bridging unit connecting two non-dethreadable rotaxanes. Our results show that incorporating the mechanophore into the macrocycle enables the synergistic toughening effect between rotaxane sliding and mechanophore scission

through the mechanism described above. We speculate that the different behavior between these two systems may arise from differences in force transmission pathways within the rotaxane-crosslinked structures, although further mechanistic investigation is needed to verify that.

Comment 3

The authors added several citations on the mechanochemistry of cyclobutane cores. However, citation is not the same as contextualization. The manuscript remains silent on how the authors' use of cyclobutane derivatives is informed by this prior work or how it advances the practical or conceptual exploitability of cyclobutane mechanochemistry.

Response 3.

We have now indicated that prior studies involving the incorporation of cyclobutane units into the backbone of crown ether derivatives have inspired our design of MINs based on such rotaxane type mechanophore. The relevant discussion has been revised accordingly in the Introduction part:

Specifically, inspired by the introduction of cyclobutane cores into crown ether backbones, we designed and synthesized a rotaxane crosslinker, of which macrocycle is cyclized by truxinate as the scissile mechanophore [22, 33, 41, 42].

Remaining concerns

Comment 1.

The authors continue to claim that "three distinct types of energy dissipation pathways coexist" in their materials without providing convincing evidence for the third mechanism.

Response 1.

We acknowledge the inability to design an appropriate control sample that would enable a direct comparison of the contribution of the woven networks to material toughness. Therefore, we have removed the attribution and related discussion regarding the role of the woven network in toughening in Figures 3 and 4 in the revised manuscript. This situation has been also explained in the discussion of Figure 3:

In addition, due to the absence of a control sample containing only the woven network, the specific contribution of the woven network converting from MINs, to the mechanical performance of **PU-RM2** remains to be elucidated. Although the role of entanglements in fracture properties is expected to be different from that in elasticity and rheology [xxx], we demonstrate that the woven network contributes to maintaining network integrity and dissipating energy in P**U-RM2** by rheological analysis of different materials (vide infra).

Nevertheless, we believe that we provide evidence for the existence of a woven network converted from the dissociation of rotaxane crosslinks. Specifically, as discussed in Figure 5 (see main text), the distinct rheological behaviors observed for **PU-C**, **PU-R**, and **PU-RM2** reflect differences in their network structures. In brief, we show that, even after the dissociation of rotaxane crosslinking points via cyclobutane cleavage in **PU-RM2**, a relatively intact network structure is retained. This is evidenced by the maintenance of a stable effective network density, as reflected in the loss modulus (E''), together with sustained energy dissipation, as indicated by the storage modulus (E'). Taken together with the structural design in **PU-RM2**, these observations suggest that the "relatively intact network structure" originates from the woven network proposed in our study.

Comment 2.

The manuscript repeatedly refers to the "woven" nature of the network without defining the term, explaining how this "wovenness" was quantified, or clarifying how such a structure forms and dissipates local stress. Statements such as "MINs gradually convert into the woven networks along with the rupture of rotaxane crosslinkers, preserving the network integrity and serving as the tertiary energy dissipation pathway" simply restate the claim without providing clarity or supporting evidence.

Response 2.

We thank the reviewer for this helpful comment. We acknowledge that we have not quantified the woven network, or provided direct evidence for its energy dissipation. Although the

effects of entanglements on polymer properties are commonly described as chain slippage and stress rearrangement (in fracture behavior), and as constrain in chain motion and retarding relaxation dynamics (in rheology), their quantitative characterization remain challenging and are still, to a large extent, treated as a “black box”. However, we succeed in proving its existence and contribution to **PU-RM2** in the rheologic level, rendering the reasonableness to deduce it works in the realistic fracture behaviors. In addition, to facilitate understanding of the concept of woven networks for a broad readership, we have provided an explanation of how the woven structure is formed in the main text, and a schematic illustration has been included in the Supporting Information to further clarify this concept:

Furthermore, woven polymer networks (WPNs), in which structurally well-defined chain entanglements are introduced through molecular design, dissipating energy by chain slippage and rearrangement of tension, are generally limited in both their stress resistance and the programmability of dissipation pathways [39, 40]……Under external stress, the system first dissipates energy through the sliding of the polymer-chain-tethered macrocycle along the axle of the rotaxane crosslinker. Once the slide reaches its limit, where the macrocycle is blocked by the axle stopper, the truxinate moiety on macrocycle undergoes selective scission with increased stress, acting as a secondary energy dissipation pathway. Subsequently, the linear chain produced by cleavage of the rotaxane's macrocycle inherently entangle with the axle chain (Scheme S7).

Response 3.
The authors continue to avoid even qualitative comparison with previously reported materials that incorporate multiple dissipative mechanisms, while presenting their work as a paradigm-shifting strategy rather than another implementation of a well-established idea.

Response 3.

We thank the reviewer for this insightful comment. As discussed in our response to comment 1 in issues addressed, we agree that the present work can be reasonably viewed as an extension of prior design strategies that combine rotaxane crosslinking with mechanophores, as well as a complement to conclusions drawn under different structural contexts.

However, we emphasize that our system achieves an effective integration of MINs and mechanophores that has not been realized in prior studies, enabling their synergistic toughening, even though direct evidence for the contribution of the woven network to mechanical performance remains limited. Therefore, we believe that our claim of advancing the paradigm of multi-pathway energy dissipation is not entirely overstated, namely, it provides insight into how such mechanisms can be synergistically combined to achieve better toughening. Accordingly, we have revised the relevant parts of the Introduction and Conclusion parts (see highlights in revised Introduction and Conclusion).

Comment 4.
The authors’ rebuttal to my objection and that of Reviewer 3 regarding the overclaimed novelty is internally inconsistent. For example, they justify the significance of their work by stating that “the toughening mechanisms when multiple (three) distinct types of energy dissipation pathways coexist remain inadequately elucidated.” The authors report another example of a material containing competing dissipative mechanisms, but do not offering new insights—at least none that I can infer—into the generalizable molecular requirements for multi-dissipative designs, nor into the molecular or physical mechanisms that control the resulting changes in bulk mechanical properties.

Response 4.

We thank the reviewer for this valuable comment. Through a comparative analysis with the work of Otsuka and co-workers, we have provided a more detailed analysis of our results, providing deeper insight into the molecular and physical picture underlying how the integration of rotaxanes and mechanophores in different combining manners within crosslinked structures influences the toughness of the material.

Comment 5 (Summary of reviewer's comments).
I do not expect the authors to answer every plausible question. Good research often raises more questions than it resolves. However, the manuscript should acknowledge the conceptual, mechanistic, and quantitative uncertainties and ambiguities, avoid overstating its novelty, and place its results faithfully within the existing literature.
Response 5.
We thank the reviewer for these constructive comments, which have helped improve the quality of our manuscript. In response, we have provided additional clarification and discussion on two main aspects: the coupling between the rotaxane structure and the mechanophore, and the concept of the woven network. We acknowledge that some questions remain to be answered in future studies, and we have made effort to clarify and discuss these points to the extent possible.
